# Structural flexibility of the human vault particle revealed by high-resolution cryo-EM and molecular dynamics simulations

**DOI:** 10.1038/s41467-026-72674-4

**Published:** 2026-05-02

**Authors:** Fabio Lapenta, Karen Palacio-Rodriguez, Sergio Cruz-León, Simone Marrancone, Jana Aupič, Nils Marechal, Alexandre Durand, Dihia Moussaoui, Sonia Covaceuszach, Bhavani Gangupam, Claudia D’Ercole, Cristian Parra, Davide Cotugno, Giulia Tomaino, Paolo Tortora, Ario de Marco, Alberto Cassetta, Alessandra Magistrato, Gerhard Hummer

**Affiliations:** 1 Laboratory for Environmental and Life Sciences, University of Nova Gorica, Vipavska cesta 13, Nova Gorica, Slovenia; 2 International Centre for Genetic Engineering and Biotechnology (ICGEB), Padriciano 99, Trieste, Italy; 3 Department of Theoretical Biophysics, Max Planck Institute of Biophysics, Max-von-Laue-Straße 3, Frankfurt am Main, Germany; 4 Istituto Officina dei Materiali, Consiglio Nazionale delle Ricerche - c/o International School for Advanced Studies, via Bonomea 265, Trieste, Italy; 5 Institut de Génétique et de Biologie Moléculaire et Cellulaire (IGBMC), Parc D’Innovation 1 Rue Laurent Fries, Illkirch Cedex, France; 6 BM29 BIOSAXS beamline, European Synchrotron Radiation Facility (ESRF), Grenoble, France; 7 Istituto di Cristallografia, Consiglio Nazionale delle Ricerche, Strada Statale 14 km 163.5, Trieste, Italy; 8 Max Planck Tandem Group Biophysics of Tropical Diseases, Faculty of Exact and Natural Sciences, University of Antioquia, Medellín, Colombia; 9 Department of Biotechnology and Biosciences, University of Milano-Bicocca, Milano, Italy; 10 Institute of Biophysics, Goethe University Frankfurt, Frankfurt am Main, Germany; 11 Present Address: EMBL Grenoble, 71 Avenue des Martyrs, 38042 Grenoble, France; 12 Present Address: IEO, European Institute of Oncology IRCCS, Department of Experimental Oncology, Milan, Italy

**Keywords:** Cryoelectron microscopy, Computational biophysics

## Abstract

Vaults are massive ribonucleoprotein complexes, highly conserved and abundant in eukaryotic cells, yet with unclear function. Their thin-walled barrel-shape architecture is composed of two symmetrical, antiparallel half-shells, each containing 39 copies of the major vault protein (MVP). The spacious lumen of the vault suggests a role in cellular transport. Although vaults are thought to undergo conformational changes to facilitate cargo exchange, the molecular basis for their inherent flexibility remains unknown. Here, we integrate cryogenic electron microscopy (cryo-EM) and multi-scale molecular dynamics (MD) simulations to reveal the structural determinants of the human vault particle’s flexibility. Cryo-EM identified two high-resolution alternative conformational states: a symmetric and an asymmetric structure, pointing to the vault shell’s structural plasticity. MD simulations of these conformations revealed that these structures are flexible and exhibit breathing-like motions, and porous solvent-exposed surfaces. Mutagenesis disrupting persistent MD-identified inter-half contacts reduced full MVP shell assembly, confirming the functional relevance of these flexibility determinants. Together, these findings establish the molecular basis for the human vault particle’s conformational plasticity.

## Abstract (web summary)

Vaults are widespread, large cellular particles with unknown function. Here, the authors reveal how vaults flex and change shape using imaging and simulations, identifying key interactions that control their structure and assembly.

## Introduction

**Fig. 1:**
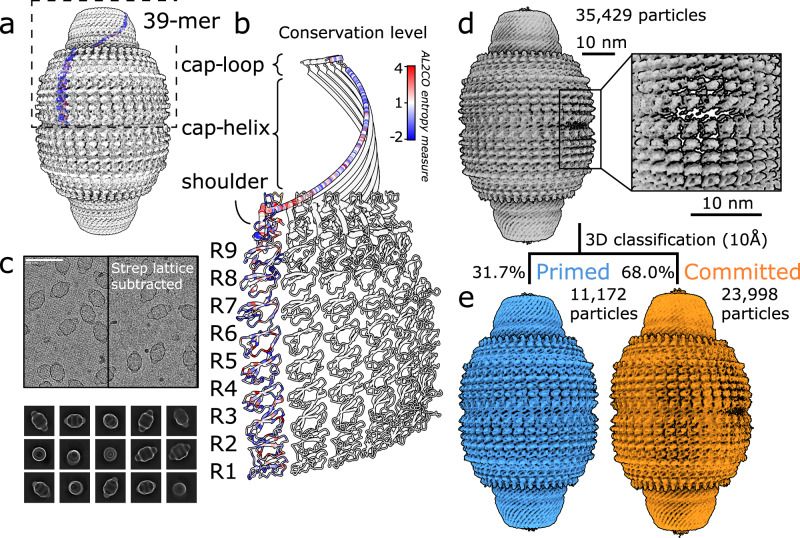
Major Vault Protein assembled into the 78-mer vault. **a** Structure of the vault composed of 78 MVP copies. MVP monomer is coloured according to residue positional entropy-based conservation scores. **b** Detailed view of MVP monomers, highlighting individual domains: repeat domains (R1 to R9), shoulder domain, cap-helix, and cap-loop (PDB:4HL8)^10^. **c** Top: representative micrograph of 6,603 accepted micrographs before and after subtraction of the streptavidin 2D crystal lattice by Fourier filtering of Bragg spots (scale bar =100 nm). Bottom: 2D class averages of the vault selected for further refinement. **d** Volume map of the initial density reconstruction obtained without imposing symmetry, based on 35,429 particles. The inset shows the symmetry-mismatched component of the map from panel d (60° rotated). **e** Cryo-EM density maps obtained from 3D classification of the particles with filter resolution of 10 Å, which shows the overall structural differences between the two conformations.

Vault particles are large ribonucleoprotein (RNP) complexes with a distinct barrel-shaped architecture and a wide lumen^1^. As the largest RNP yet described in eukaryotes, vaults are composed primarily of 78 copies of the 99 kDa major vault protein (MVP), assembled into a symmetric protein shell^2^. Pioneering structural studies on the vault from *Rattus norvegicus* revealed that two 39-mer halves of the vault particle establish an anti-parallel association in the central area of the barrel via their N-terminal domains. This assembly forms a particle with a large inner volume of around 35,000 nm^3^ and dimensions of 40 nm × 40 nm × 66 nm^3–6^ (Fig. 1a). The unique architecture of the vault is inherently tied to the structure of MVP, with each MVP subunit consisting of 12 distinct domains (Fig. 1b). Nine N-terminal antiparallel three-stranded β-sheet repeat domains (R1-R9) extend from the midsection and compose the central body of the vault^6^. The subsequent shoulder domain, with its globular α/β fold, is homologous to the Stomatin, Prohibitin, Flotillin, and HflK/C (SPFH) domain, characteristic of vault-like membrane-associated protein assemblies^7,8^, and forms a hinge between the central body and the C-terminal caps^9^. The latter are formed by long helical domains known as the cap-helices, which are engaged in an extensive coiled-coil helical bundle that stabilizes the vault^3^. Finally, at the two tips of the barrel, the C-terminal regions of MVP form the cap-ring, whose partially disordered structure folds inwards forming a β-barrel pore with a positively charged surface^6,10–12^.

With notable exceptions (including fungi, insects and likely plants), vault’s phylogenetic distribution is widespread across the major eukaryotic supergroups and possibly present already in the last eukaryote common ancestor (LECA)^13,14^. The high energetic cost of vault synthesis by the cell and its evolutionary conservation imply the vault plays a pivotal role in the cellular environment. Nevertheless, while vaults have been implicated in numerous cellular processes (i.e., intracellular transport^15,16^ and signalling^17^, innate immune response^18,19^, DNA damage repair^20^, apoptosis^21,22^, among others), their primary function remains unclear^23,24^. Vaults also have a long-debated role in multi-drug resistance^25–28^, neurodevelopmental disorders^29^ and recent findings have suggested their possible involvement in protein folding^30^. Protein partners associated with vault particles in vivo include the vault-associate protein telomerase TEP1^31^ and the poly-(ADP-ribose) polymerase vPARP^32^. Vaults are associated also with short non-coding vault RNAs (vtRNA), which are not restricted to vaults and are found in the cytosol^25,31^. Additionally, electron-tomographic imaging of eukaryotic cells revealed vault particles capable of accommodating ribosomes within their inner cavity^33^, demonstrating their ability to encapsulate also very large cellular components. The purpose of compartmentalising these molecular components within the vault is still obscure, compounding its elusive cellular role. Furthermore, there are conflicting reports whether in cells encapsulation of molecular cargo occurs concomitantly with the vaults initial assembly on the polyribosome or, instead, the vault particles can dynamically incorporate and release cargo during their lifecycle by undergoing partial disassembly and reassembly^32,34^.

Recent cryo‑ET studies of *D. discoideum* cells have failed to detect isolated 39‑mer half vault subunits^35^. In contrast, the dynamic exchange of half vaults was observed in vitro^36^. The equilibrium between the full vault and the half vaults was shown to depend on solution conditions, such as pH, salt concentration and temperature^37–39^. This coupled with their large inner cavity and low immunogenicity raised a significant interest in the vault particle for a variety of potential biotechnological applications^40–42^. Diverse novel uses of the vault have been explored and include encapsulation systems for host immunization of protein cargo^43–45^, antibody functionalization for targeted delivery^46,47^, genetically encoded devices to capture and store mRNA for the retrospective analysis of past transcriptomic states^48^, and innovative approaches currently under development, such as the encapsulation of whole adeno-associated viruses for gene theraphy^49^. Regardless of the extensive research into the vault particle as a potential delivery vehicle, the molecular details of the vault particle plasticity and its regulation remain undefined.

By performing 3D classification of cryogenic electron microscopy (cryo-EM) maps of the vault from *R. norvegicus* Guerra et al. recently distinguished regularly assembled, symmetric, vault particles from quasi-symmetric vaults with distortions at the waist, resulting in partial opening of the vault^50^. Based on their observations, the authors suggested that opening the vault into two halves is a multi-step process that occurs through a series of intermediate states, initiated by a prominent structural alteration at the N-terminal waist region. However, the low resolution prevented the construction of an atomistic model and a more detailed mechanistic analysis of the disassembly process.

In this work, we investigate the structure and dynamics of the human vault by cryo-EM and molecular dynamics (MD) simulations. Cryo-EM analysis captured vaults in two distinct conformations. While in the highest-resolution conformation the vault assumed a symmetric closed state, the dominant conformation exhibited asymmetries at the waist. Atomic-level structures were determined and used as starting points for MD simulations. These simulations confirmed the stability of the two conformations despite their large flexibility, revealed mechanisms for passive diffusion of small molecules in and out of the vaults, and shed light on the specific interactions responsible for the stability of the vault particle. Together, our results uncover the intrinsic flexibility of the human vault particle at atomic resolution.

## Results

### MVP exhibits substantial conformational plasticity

Assembly of MVP on the polyribosome has been observed^51^ and MVP alone is sufficient for vault particle formation^2^. In addition, recombinant vault particles have been expressed and purified from the yeast *Komagataella phaffii* (formerly *Pichia pastoris*)^52^. This organism lacks an endogenous vault gene in its genome and is characterized by rapid growth and stable gene expression^53^. For our work, we relied on a strain of *K. phaffii* bearing an integrated human *MVP* gene (hMVP) under the control of a constitutive promoter (pGAP)^54^, allowing intracellular expression of hMVP. The purification procedure, based on previously reported works^54,55^, consisted of an ultra-centrifugation step followed by RNase treatment prior to size-exclusion chromatography (SEC). The protein was analyzed by multi-angle-light scattering coupled to size exclusion chromatography (SEC-MALS) to assess the assembly of the complex (Supplementary Fig. 1). The molecular weight of the main peak (7.66 ± 0.6% MDa) confirmed the correct assembly of the MVP into a full 78-mer vault particle, with only a minimal amount of 39-mer half vaults detected ( < 5%). Batch small angle X-ray scattering (SAXS) analysis confirmed the assembled 78-mer assumes the expected shape in solution (Supplementary Figs. 2 and 3). Moreover, estimation of molecular weight from SAXS data confirmed a small amount of half vaults (Supplementary Table 1).

The protein was biotinylated and subsequently immobilized on streptavidin-coated affinity grids to increase local concentration and overcome issues with preferred orientation of the particles on the grid, then vitrified and imaged using a 300 kV cryogenic electron microscope (Supplementary Table 2). The majority of the vaults were fully assembled on the grid (Fig. 1c) and micrograph analysis showed only a small population of half vaults (9.6%). For single particle analysis (SPA), we considered only intact vault particles, whereas half-vault and collapsed vault particles were identified and excluded (Supplementary Fig. 4).

Initial 3D refinement without enforced symmetry revealed a density map exhibiting the characteristic barrel-shaped vault architecture, albeit with a visible distortion detected at the midsection of the vault resulting in deviations from the presumed 39-fold dihedral symmetry (Fig. 1d). The lower density observed in the section of the cryo-EM map corresponding to the vault waist suggested a higher degree of flexibility in this region (Supplementary Fig. 5). To evaluate the structural heterogeneity of the full vault particles, we performed three-dimensional variability analysis (3DVA) across the whole particle stack^56^. 3DVA revealed continuous flexibility within the vault, characterized by different modes of stretching and compression across the whole particle, which resulted in the formation of multiple symmetry-mismatch components, with the most prominent distortion found at the waist (Supplementary Movie 1 and Supplementary Fig. 5). Analogously, principal component analysis (PCA) performed on the entire particle set^57^ revealed a heterogenous conformational landscape, predominantly characterized by two major populations (Supplementary Fig. 6), discernible by the presence or absence of a rupture at the vault waist caused by the lower resolution of the antiparallel R1 domains.

Finally, to construct cryo-EM maps representative of the detected conformational states, we performed an unsupervised 3D classification of the particles^58^ (filter resolution 10 Å). We identified two major subpopulations, distinguished by presence or absence of the D39 point-group symmetry that is a characteristic feature of the vault. These subpopulations comprise 32% and 68% of the particles, respectively. In line with a previous cryo-EM analysis of the vault from *R. norvegicus*^50^, where a distorted conformation of the particle was detected, we named these subpopulations as primed and committed, for the symmetric and asymmetric conformation respectively (Fig. 1e). While these two conformations were distinguishable from the cryo-EM dataset, SAXS was not able to discriminate between them due to their similarity in terms of overall size and molecular shape (Supplementary Fig. 3). In summary, analysis of the cryo-EM dataset suggests that, contrary to the common view of the vault particle as a rigid fully symmetric protein shell composed of two tightly interacting half vaults, the vault is instead associated with substantial conformational heterogeneity.

### An extensive network of polar interactions stabilizes the symmetric primed vault

**Fig. 2:**
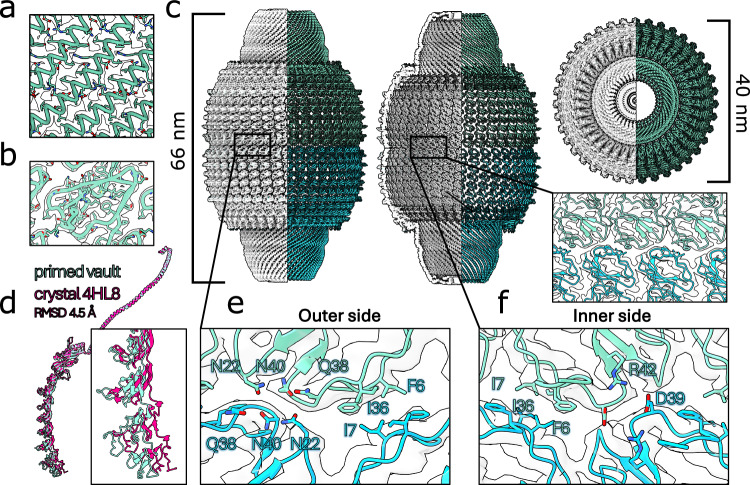
Structural details of the vault structure in primed conformation. **a, b** Close-up views of the fit between the atomic model and the map for the cap-helix (**a**) and the R5 domain (**b**). **c** Vault structure in primed conformation. The map is cropped in half longitudinally, with the corresponding atomic model showed side-by-side. The left panel shows the intact vault. The central panel shows the model clipped in the middle. The right panel shows the top view of the protein. The inset shows a detail of the fit between the map and the atomic model, represented as cartoon, at the midsection where the two 39-mer halves meet. **d** Structure alignment of hMVP (from this work) and rMVP structure obtained by X-ray crystallography (PBD: 4HL8)^10^, in green and pink, respectively. Overall root-mean-square deviation (RMSD) between the two structures shown. The detail in the panel shows the R1-R4 domains. **e** Close-up of the outer side of the vault waist in the primed conformation (local refinement map), showing the polar triad (Asn22, Gln38 and Asn40) and the hydrophobic patch (Phe6, Ile7 and Ile36). **f** Close-up of the inner side of the vault waist in the hMVP structure (local refinement map): the salt bridge between Asp39 and Arg42 and the hydrophobic patch (Phe6, Ile7 and Ile36). All density maps are displayed at contour level of 0.8.

Single particle reconstruction of the primed conformation was carried out imposing 39-fold dihedral symmetry, resulting in a high-resolution refinement of the vault at 3.09 Å resolution, as determined by gold-standard Fourier shell correlation (GS-FSC) at an FSC threshold of 0.143 (Supplementary Fig. 7). The lower density region at the N-terminal waist was improved by local refinement, yielding an additional map with GS-FSC resolution of 3.53 Å. This refinement provided better density for constructing the R1-R2 repeats in the atomic model (Supplementary Fig. 8). This approach enabled tracing of the carbon backbone from residue 3 to residue 814, with the notable exception of two flexible loops (residues 429-449 and 608-619), which were also missing in previously published structures of MVP from *R. norvegicus* (rMVP). The map showed clear density signals for the sidechains along the whole sequence of hMVP (Figs. 2a and 2b and Supplementary Fig. 9).

The reconstructed atomic model presented the characteristic vault architecture, with a shell composed of 78 repeated structural units of MVP, tightly interacting around a large inner cavity (Fig. 2c). As expected from the high sequence homology (91.06%) between rMVP and hMVP, the structure of hMVP bore high similarity to previously deposited structures of its murine counterpart. The root mean square deviation (RMSD) between primed hMVP and primed rMVP (PDB: 7PKR)^50^, also resolved by cryo-EM, corresponded to 1.07 Å (Supplementary Fig. 10). Conversely, RMSD (4.47 Å) was significantly higher when comparing to rMVP resolved by X-ray crystallography (PDB: 4HL8)^10^ (Fig. 2d). The largest structural divergence between hMVP and crystallized rMVP was observed in the curvature of the N-terminal R1-R5 domains, a difference likely due to the absence of crystal packing contacts, as suggested in a previous work^9^.

A detailed analysis of the human vault in the primed conformation enabled the identification of interfacial contacts crucial for the assembly of MVP chains into the vault particle. The N-terminal R1 domains converge at the interface of the two 39-mer halves, establishing a network of interactions that tie the antiparallel halves together at the midsection of the vault. Each R1 domain interacts with two opposing R1 domains, forming an interface characterized by a hydrophobic patch composed of residues Phe6, Ile7, and Ile36 from two interacting chains. These residues form a tightly packed hydrophobic cluster, surrounded by polar amino acids that further stabilize the interaction between the two antiparallel R1 (Fig. 2e). Additionally, a network of polar interactions is established along the outer side of the vault, involving amidic residues (Asn22, Gln38 and Asn40) from opposing R1 chains (Fig. 2e). Likewise, inter-chain salt bridges between Asp39 and Arg42 of opposing chains propagated along the inner waist of the vault (Fig. 2f). The lateral contacts between MVP monomers primarily reside in the helix-cap region, with 132 residues involved in neighbouring contacts between repeat domains and shoulder domain (residues 1-646), compared to 144 interacting residues in the helix-cap (residues 647-814) (Supplementary Figs. 11 and 12). Analysis of lateral contacts identified several ionizable residues involved in MVP:MVP interaction in three clusters along the repeat domains at R1 (Arg9:Gln21), R5-6 (Asn234:Glu257:Thr245, Gln298:Glu305) and R8-9 (Asp383:Arg474, Arg461:Glu492, Lys476:Glu485), at the shoulder domain (Asp530:His534:His592, Arg536:Glu644) and in the helix cap (Glu700:Ser713, Arg729:Glu736, Arg766:Glu769). Taken together with the antiparallel interactions between Asp39:Arg42 and the triad Asn22:Gln38:Asn40, protonation-induced changes in this network of polar and ionic interactions is a possible cause of the pH-dependent destabilization of the vault observed in other works^38,39^ (Supplementary Fig. 13).

The helix-cap stabilizes the half-vaults by establishing a wide 39-mer helix bundle. Analysis of the residue composition at the helix interfaces revealed an abundance of alanine and leucine residues densely packing the helices close together, in addition to charged residues on the outer position of the helix ridge (Supplementary Fig. 11).

### Transient interactions at the vault waist result in symmetry breaking and pore formation

Refinement of the volume corresponding to the committed conformation (without imposing symmetry) yielded a map with a dominant D39-point symmetry, similar to the primed conformation, yet with a clear symmetry-mismatched component, most prominent at the waist (Fig. 1e). To better characterized this more flexible region, we performed a further refinement employing symmetry relaxation, a method that only limits the pose search for each particle to the original symmetry group of the assembly for the purpose of an asymmetric refinement^59^. This approach yielded a map at 4.45 Å GS-FSC resolution with a prominent loss in local resolution in correspondence with the symmetry-mismatched component at the waist (Supplementary Fig. 14). The Sampling Compensation Factor (SCF*) of 0.906 suggested that Fourier space is well-sampled, ruling out insufficient angular coverage as the cause of the lower resolution^60,61^. In contrast, the conical FSC Area Ratio (cFAR), which can be used to probe the directional anisotropy^61,62^, revealed direction-dependent loss of signal. Taken together, this indicated that the anisotropy could have arisen from the heterogeneity of the sample or from poorly aligned particle (**Supplementary Discussion**).

**Fig. 3:**
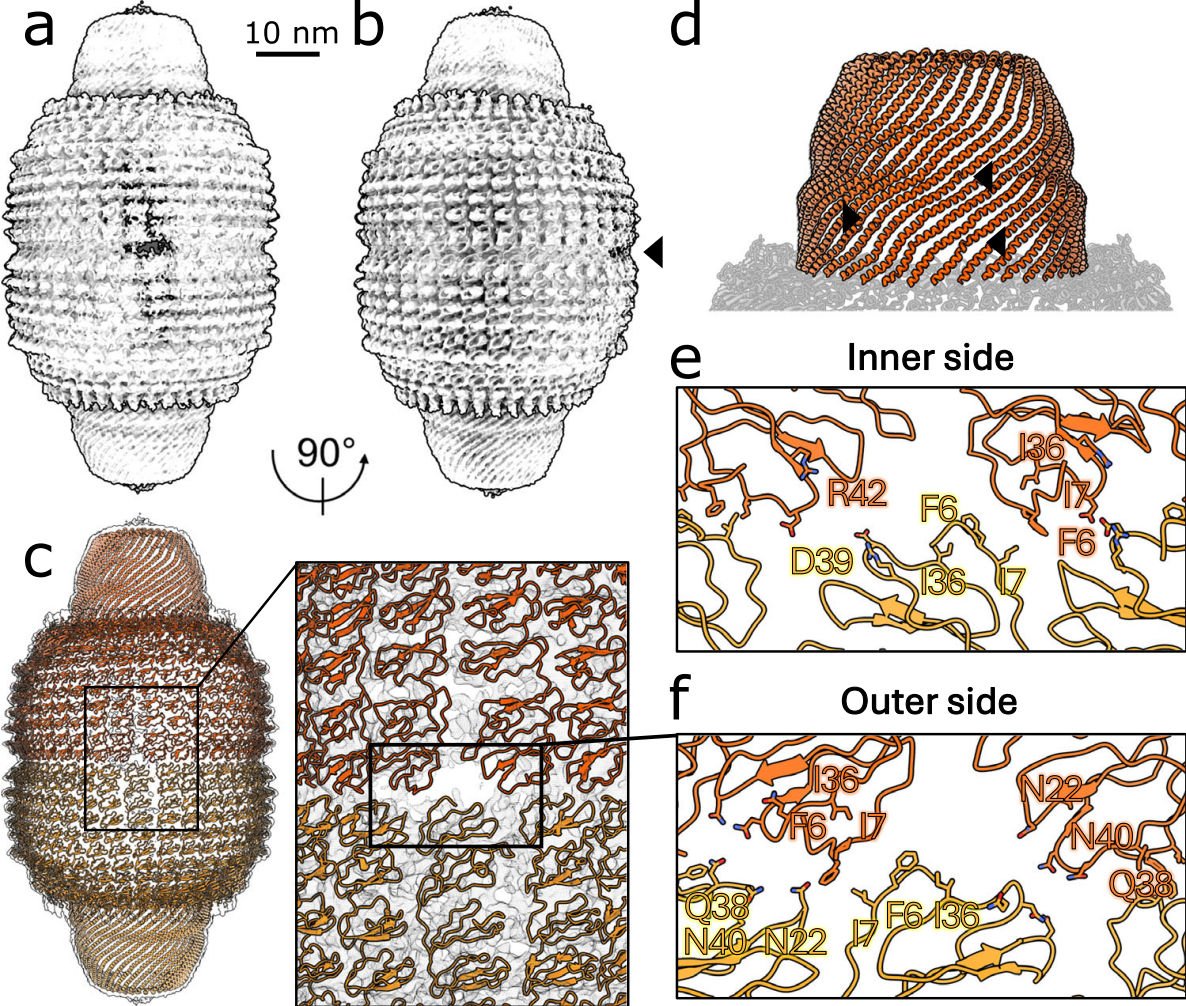
Volume and structure of the vault in committed conformation. **a, b** Cryo-EM density map of the vault in committed conformation (contour level 0.35). The black arrow indicates the region of distortion at the waist. **c** Atomic structure of the vault in committed conformation, superimposed onto the cryo-EM map. (Inset) A detail of the atomic structure of the vault in committed conformation, with the density map shown as black mesh (contour level 0.35). **d** Structure of the helix bundle in the cap-helix. The black arrows indicate the kinks in the helices induced by the conformational change. **e, f** Atomic model of the vault’s waist, shown from the inner side (e) and outer side (f). Cartoon representation of the vault in committed conformation, with residues at the interface of the two 39-mer MVP halves shown as sticks, illustrating the loss of contacts in the region of greater distortion. Hydrophobic patch residues (Phe6, Ile7 and Ile36), the polar residues of the polar triad (Asn22, Gln38 and Asn40), as well as the charged residues (Asp39 and Arg42), are shown as sticks.

The map showed the vault committed conformation being overall comparable to the primed counterpart, with major differences clearly noticeable at the waist, corresponding to the N-terminal R1-R6 domains of 8 chains in each of the two 39-mer halves (Figs. 3a and 3b). This deformation was more pronounced in one 39-mer half than in the other, reflecting the presence of a longitudinal compression between the primed and the committed conformations.

The atomic model of the committed vault was obtained through symmetry relaxation and molecular dynamics flexible fitting (MDFF)^63,64^ of the symmetric higher-resolution primed structure into the committed density map (Fig. 3c). Besides the extensive distortion of the waist observed in the committed conformation (Fig. 3b), we noted further structural deformations in the cap-helix, where several kinks in the α-helices were observed (Fig. 3d). Additionally, we performed a local refinement of the midsection of the vault aimed at improving the resolution of the R1-R2 domains at the waist, which resulted in a local refinement map at resolution of 6.06 Å (Supplementary Fig. 15).

The heterogeneity of the particles used in the reconstruction of the map resulted in a markedly lower resolution in the symmetry-mismatched component at the waist, leading to a low-confidence model for the distorted repeat domains. Nonetheless, the curvature of the vault in the region of greatest displacement visibly forced the two 39-mer halves apart and determined an overall loss of lateral contacts along R1-R6 domains and at the level of opposing R1 domains. Specifically, the interaction between two opposing antiparallel R1 domains was lost due the disruption of inter-chain salt bridges and the hydrophobic patch (Fig. 3e and f).

**Fig. 4:**
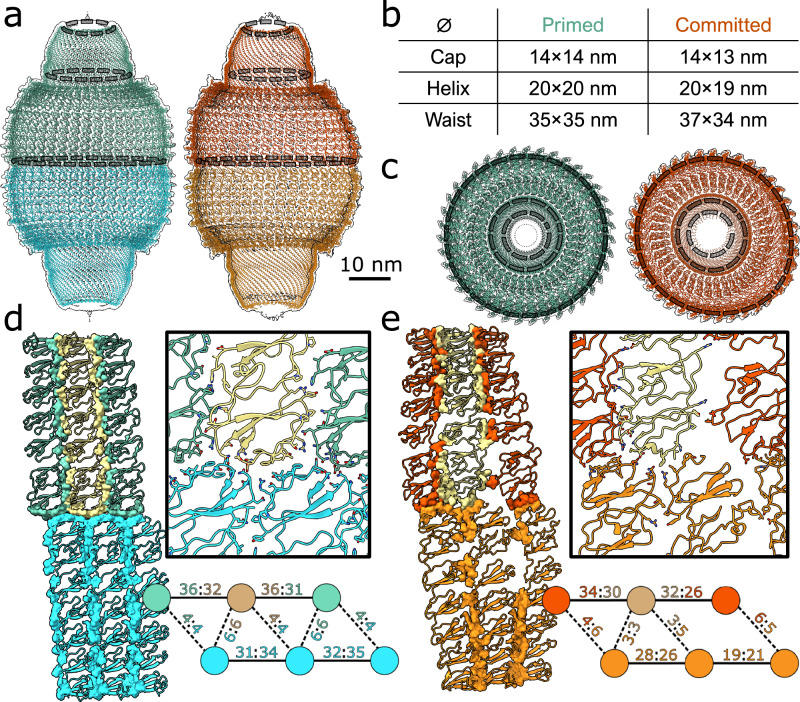
Structural comparison between vault in primed and committed conformations. **a** Clipped representation of vault in primed and committed conformations (left and right, respectively). Cartoon representation of the backbone and map shown as a surface. The dashed circle indicates the cross sections used to calculate the diameter. **b** Table comparing the diameter of the two structures (see Methods) at different cross-sections (shown in a and c as dashed circles). **c** Top view of the upper 39-mer half of the vault in primed and committed conformations (left and right, respectively). In (a, c), contour levels are set at 0.9 and 0.4 for primed and committed, respectively. **d, e** Left panel, lateral view of six MVP chains (residues 1-379) in the primed (d) and committed (e) vault structures, respectively. Cartoon representation of the model with visible surface of the residues burying at least 11Å^2^ of area at the interface of different MVP chains. The top-right panels show a detailed view of the six chains with contacting residues as sticks. The bottom-right panels show a schematic representation quantifying the number of contacting residues among the six chains (between residues 1-379), illustrating the quaternary organization of the vault protein. Each polypeptide chain is shown as a coloured circle. Black solid lines connect chains within the same 39-mer half of the vault, whereas dotted lines indicate inter-subunit contacts that bridge the two 39-mer halves. The numbers on each connection (separated by a colon) identify the number of residues from each chain that participate in these intermolecular contacts.

Next, the structure of the vault in the committed conformation was closely compared to that of the primed conformation. Most noticeably, the symmetry-mismatched component in the committed conformation stretched the vault’s waist. Compared to the diameter in the primed conformation (35 nm × 35 nm), the committed conformation assumed a more elliptical shape, with a diameter of 37 nm × 34 nm. (Fig. 4a and b). Moreover, the diameter of the helix bundle in the cap helix is smaller than in the primed conformation by up to about 1 nm (Fig. 4b and c). This distortion was markedly more prominent in one 39-mer half than the other, indicating that relaxation at the waist, associated with the loss of N-terminal contacts between the two halves, propagated longitudinally and culminated in changes in the cap-helix (Fig. 4a). This transition was also captured by the 3DVA performed on the whole particle set (Supplementary Movie 1 and Supplementary Fig. 5).

Analysis of the contacts between R1-R7 domains in the primed conformation showed tightly packed repeated interfaces between chains. Each MVP monomer interacted with four other chains, two along the waist of the vault and two opposing on either side (Fig. 4d). The waist interactions are stabilized by hydrophobic patches, whereas a network of polar interactions zips up the opposing chains. In the committed conformation, by contrast, MVP chains located in the most distorted region of the waist showed an overall loss in the number of lateral contacts and a complete lack of interactions between two opposing chains (Fig. 4e). This ultimately resulted in the formation of a gap between the two antiparallel 39-mer halves and of longitudinal pores along the interface of parallel MVP subunits (Figs. 3c and 4e).

### The half vault

The presence of a small population of 39-mer half vaults on the micrographs allowed us to reconstruct a low-resolution map of the disassembled half particle at resolution of 9.89 Å (Supplementary Fig. 16). The reconstructed volume indicated a clear deviation from the regular conformation assumed by the halves in the whole vault. The waist appears distorted, and the heterogeneity of the particles resulted in a lower-density region in the map, corresponding to the R1-R6 domains. This suggests a higher degree of flexibility in this region, in line with the fewer lateral contacts present. The loss of structural constraints at the waist clearly affects the curvature of the six initial repeat domains in the half vaults, which closely resemble the 39-mer halves of the vault in committed conformation.

### The vault particle is a flexible and stable protein cage

To obtain a detailed view of the conformational dynamics of the vault complex, we performed classical all-atom molecular dynamics (AA-MD) and coarse-grained molecular dynamics (CG-MD) simulations, focusing on the primed, committed, and half-vault conformations. Starting from the distinct cryo-EM structures, we built atomistic models to characterize the dynamic interactions of MVP subunits.

**Fig. 5:**
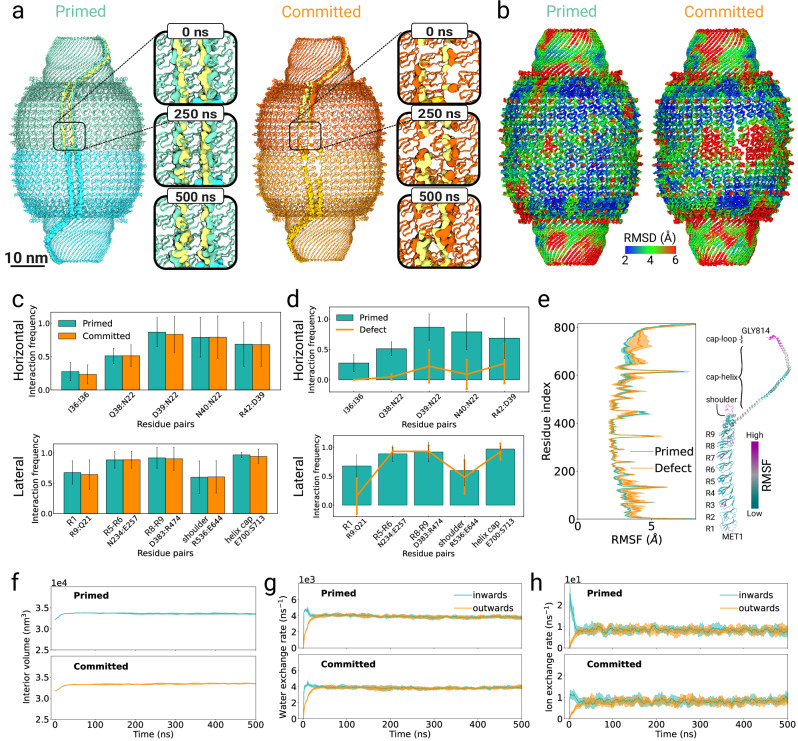
All‑atom MD simulations of the vault in primed and committed conformations. **a** Initial atomic models (cartoon) of the vault in the primed (left) and committed (right) conformations. Surface renderings highlight inter‑MVP contacts; inset panels show representative snapshots of MD trajectories. **b** RMSD over the last 450 ns of simulation, mapped onto the initial atomic structures of the vault in primed (left) and committed (right) conformations. Structures are aligned to the Cα atoms of residues with RMSF < 3.5 Å. **c** Persistent inter‑MVP contacts identified in the simulations. Top: Waist region – most frequent contacts per interaction type (hydrophobic I36:I36, polar Q38:N22, D39:N22, N40:N22, and salt‑bridge R42:D39). Bottom: Lateral interfaces – dominant contacts in R1 (R9:Q21), R5‑R6 (N234:E257), R8‑R9 (D383:R474), shoulder (R536:E644), and helix‑cap (E700:S713). Bars correspond to van der Waals (vdW) contacts frequency in the primed (blue) and the committed (orange) conformation. Data are presented as mean values  ±  SD (*n*  =   7,956 for horizontal and *n*  =   3,978 for lateral interaction frequencies). **d** Effect of defect on vdW interactions. Top panel: Waist contacts – blue bars (vdW persistence in primed vault); orange line (vdW frequency between chains bordering the opening in committed vault). Bottom panel: Lateral contacts (*n*  =  306 and *n*  =  408 for horizonal and lateral interaction frequencies surrounding the defect area, respectively). **e** RMSF per residue for the full trajectory. Left: RMSF vs. residue index. Average over all chains in two primed replicates (blue) and chains adjacent to the rupture/defect in the committed conformation (orange). Right: RMSF values colour‑mapped onto a single MVP monomer. **f** Interior volume of the vault particle as a function of time over the MD trajectory in primed (top) and committed conformation (bottom). **g** Water and **h** ions exchange across the vault. The exchange rates inwards (blue) and outwards (orange) were quantified by counting the events in which water/ions crossed from the exterior to the interior volume of the vault in the primed (top) and committed (bottom) conformations. Panels **e,** **f,** **g**, and **h** display the mean values (solid line) ± SD (shaded regions). Source data are provided in Source Data file.

In our simulations, we included all MVP subunits (residues 1–814), fully solvated in water with Na⁺ and Cl⁻ ions. We performed two replicates of AA-MD simulations for each initial conformation, each running for 500 ns. These AA-MD simulations involved systems of ~18.4 million atoms (Supplementary Table 3), highlighting the feasibility of studying such a large biological assembly, thanks to advances in supercomputing resources^65^. The AA-MD simulations revealed that both conformations largely maintained their overall structural integrity throughout the simulation, as illustrated by representative snapshots from the MD trajectories (Fig. 5a). Nevertheless, the interfaces at the level of the symmetry-mismatched component in the committed conformation displayed greater variability, with the prominent rupture at the waist showing attempts to close but ultimately forming fewer stable contacts compared to the primed conformation.

Both conformations exhibited comparable flexibility during AA-MD simulations, with the committed conformation displaying more pronounced fluctuations specifically at the symmetry-breaking interface. To visualize the dynamic regions of both conformations, we mapped the RMSD from the starting model after the first 50 ns of AA-MD onto their initial atomic structures, aligning them to the Cα atoms of residues with root-mean-square fluctuation (RMSF) < 3.5 Å (Fig. 5b). Localized patches with high RMSD were observed across the waist, cap, and shoulder regions in both conformations. Notably, a high flexibility region in the committed state is found near the symmetry-breaking interface, coinciding with the low-resolution region in the cryo-EM map. However, high-resolution regions (e.g., small parts of the primed conformation waist and both conformations around the shoulder and cap regions) also exhibited significant flexibility, confirming that vault dynamics are intrinsic and not merely an artifact of cryo-EM resolution. These observations collectively indicate that the vault particle maintains inherent structural flexibility in both conformations.

Analysis of persistent residue-residue contacts during MD simulations (Fig. 5c) confirmed the stability of key interaction networks in both conformations. Long-lived contacts were conserved at the horizontal waist (inter-half contacts) and lateral MVP interfaces (intra-half contacts), validating the cryo-EM-derived atomic models. At the waist, the inter-half interface maintained stable polar interactions (N22:Q38, N40:Q38, D39:R42 salt bridge) and a dominant hydrophobic interaction (I36:I36). In lateral regions, MVP:MVP polar contacts predominated (e.g., R9:Q21, N234:E257, D383:R474), with additional contacts summarized in Supplementary Figs. 22 (lateral) and 23 (horizontal).

At the symmetry-breaking interface of the committed conformation (“defect” in Fig. 5d), horizontal waist contacts were significantly disrupted, particularly the hydrophobic patch, while lateral interactions at higher MVP domains remained largely intact. Notably, some polar contacts at the rupture site are found but with reduced frequency. This differential loss of horizontal contacts without major disruption to lateral interactions highlights how localized flexibility at the waist enables conformational plasticity, consistent with the observed dynamic equilibrium between primed and committed states.

We further quantified flexibility by calculating average RMSF across all MVP chains for both conformations (Fig. 5e, Supplementary Fig. 19). RMSF confirmed the waist and cap regions as primary flexible sites. Mapping RMSF onto a single MVP chain (Fig. 5e, right panel) revealed high flexibility in waist loops, R domain loops, shoulder, and the cap loop. We note that the flexibility at the cap could be the result of not including the full cap domain in the simulations, the structure of which has only recently been reported^11,12^. Interestingly, comparison of primed conformation RMSF with committed conformation RMSF at the symmetry-breaking interface (Fig. 5e, left panel) shows increased flexibility at the rupture mostly at the waist and cap regions. This increased flexibility, particularly at the waist was consistent with the lower resolution of the cryo-EM model and the stretching-compression motions observed in the 3DVA.

Additionally, we observed breathing-like motion in the trajectory. These motions were captured with PCA and projected onto the top 3 principal components (PCs), which account for about 70% of the variance (Supplementary Fig. 20). The collective motions show asymmetric deformation of the vault particle and torsional motions with global fluctuations of the entire particle (see Supplementary Movie 3). Despite these fluctuations, the AA-MD simulations accurately captured the general shape of the vault particle with remarkable agreement with the experimental structures, as shown by particle diameter measurements at the waist, helix, and cap regions (Supplementary Fig. 17).

We checked if the breathing-like movements were related to the particle’s ability to exchange solvent with the environment. We measured the internal volume of the vault particle during the simulation and the water and ion exchange rates over time. Water exchange reached equilibrium at ~400 molecules/ns (Fig. 5g), a value comparable to that reported for a bacteriophage particle^66^. Sodium and chloride ions achieved equilibrium exchange rates of ~10 ns⁻¹ after 50 ns (Fig. 5h), further confirming vault permeability to water and ions. Interestingly, sodium ions localized to specific hotspots, e.g., D39:R42 salt bridges, R3–R4/R6–R7 interfaces, shoulder, and helix-cap regions (Supplementary Fig. 21). Exchange rates stabilized after ~50 ns, indicating simulation equilibration.

**Fig. 6:**
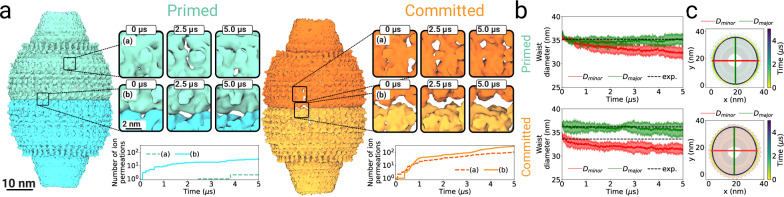
Coarse-grained MD simulations of the vault in primed and committed conformations. **a** Representative coarse‑grained structures of the vault in the primed (left) and committed (right) conformations. Surface renderings show the overall particle; inset panels focus on (**a**) MVP–MVP interfaces and (**b**) the waist region in the interface between vault halves. Cumulative number of ion permeations through zoomed-in regions (**a**) and (**b**) in the vault shell (bottom), measured by tracking ions entering and exiting the vault through pores in each region. **b** Time series of the vault’s waist diameter in the primed (top) and committed (bottom) conformations. Diameters were obtained by fitting an ellipse to the backbone bead positions of residues D39, with major and minor diameters shown in green and red, respectively. Mean values (solid lines) ± SD (shaded regions) from three simulation replicates are plotted. Dashed lines indicate diameters from the atomic model. **c** 2D representation of the fitted ellipses for the vault’s waist diameter over time in the primed (top) and committed (bottom) conformations. Half-vaults are shown in transparency for reference. Each ellipse is constructed using the major and minor diameters fitted to the backbone bead positions of residues D39. The colour scale represents the time point at which each ellipse was fitted, with one ellipse plotted every 150 ns. Source data are provided as a Source Data file.

Although AA-MD simulations provide atomic-level detail, their accessible timescales remain relatively short compared to those needed to capture large conformational changes. CG-MD simulations address this limitation by enabling the exploration of larger-scale dynamics over extended timescales. For the human vault particle, the system size was reduced to ~1.6 million beads, allowing us to perform 5 μs of simulation for three replicates per initial configuration to further assess the vault’s flexibility. Representative coarse-grained structures of the primed and committed conformations revealed the formation of dynamic horizontal pores at the waist and vertical pores between MVP subunits (Fig. 6a and Supplementary Movie 2). Quantification of cumulative ion permeation through specific regions at the vault shell shows that regions with visually larger pores allow greater ion permeation (see bottom panels in Fig. 6a and Methods).

The vault particle exhibited remarkable flexibility in CG-MD simulations, particularly at the waist. To quantify this dynamic behaviour, we tracked changes in waist diameter over the course of the simulation by fitting an ellipse to the backbone bead positions of residues Asp39, with the major and minor diameters shown in green and red, respectively (Fig. 6b). The time series data revealed substantial flexibility at the waist, with good agreement between the major diameter of the fitted ellipses and the cryo-EM atomic model. These fluctuations suggested the presence of a general breathing motion, further confirmed with PCA (Supplementary Movie 3).

A two-dimensional representation of the fitted ellipses over time illustrated how the vault gradually adopted a more ellipsoidal shape as the CG-MD simulations progressed (Fig. 6c). This ellipsoidal shape in both the primed and committed conformations associated with the expansion of the waist pores. We also measured the diameters of the cap and helix regions for both vault configurations (Supplementary Fig. 18a). Our CG-MD simulations systematically underestimated the cap diameter, likely due to the absence of the C-terminal regions of the MVP. By contrast, the helix diameter closely matched the cryo-EM models.

Finally, to further characterize vault shell flexibility, we analysed individual half-vaults starting from both the committed (Supplementary Fig. 25) and primed (Supplementary Fig. 26) conformations, comparing AA-MD and CG-MD simulations with experimental cryo-EM data. AA-MD simulations of the half-vault system revealed greater structural changes, particularly at the waist, compared to the full vault particle. This is reflected in the RMSD from the last 450 ns of AA-MD simulations (Supplementary Fig. 25b-left), where higher RMSD values indicate increased flexibility and more pronounced conformational changes. Interestingly, R1-R6 regions show large variations in RMSD, consistent with the large distortions in the experimental cryo-EM map of the 39-mer halves (Supplementary Fig. 25e-bottom). RMSF measurements for the half vault in AA-MD also confirms increased flexibility between domains R1-R6 compared to the full vault particle (Supplementary Fig. 19-bottom). The RMSD of CG-MD simulations over the last 4.5 μs showed not only large instability at the waist but also increased flexibility at the cap region (Supplementary Fig. 25b-right). Tracking the waist diameter revealed greater fluctuations and larger amplitude breathing movements compared to the full vault (Supplementary Fig. 25c), underscoring the enhanced flexibility of the half-vault.

Representative snapshots from the CG-MD trajectory, viewed laterally and from an internal bottom-up perspective, revealed prominent structural deformations, including the formation of large vertical pores and inward movement of the MVP N-terminal regions at the waist (Supplementary Fig. 25d). The low-resolution cryo-EM map (Supplementary Fig. 25e) further suggested significant conformational flexibility, with the waist shape closely resembling the deformations observed in the CG-MD simulations. In contrast, simulations starting from the primed half-vault showed smaller variations in waist diameter and overall lower RMSD values (Supplementary Fig. 26).

### Frontal interactions between R1 domains regulate the equilibrium between full and half vaults

To dissect the individual contributions of the interfacial residues at the vault’s waist, we performed targeted mutagenesis and generated four MVP variants with substitutions of amino acids that stabilize the tight packing between the two 39-mer halves in the primed conformation, but are ruptured in the committed conformation. Specifically, we produced the following mutants: MVP(Q38A, N40A) and MVP(D39A, R42A) designed to disrupt the polar network and salt bridging at the interface between the antiparallel halves; and MVP(I36D) containing a single charged amino acid substitution intended to disrupt the hydrophobic patch at the residue that presented a more persistent contact during MD simulations. These variants were analyzed using SEC-MALS and negative-stain TEM. Our analysis revealed that all tested mutations increased the population of half vaults with respect to fully assembled vault particles, albeit to a different extent (Supplementary Fig. 27). Among them, the MVP(I36D) variant caused the strongest destabilization, characterized by a pronounced predominance of half-vault species. Contrarily, variants containing substitutions in polar residue pairs showed only a slighter decrease in the ratio of full vaults over half vaults, indicating the importance of the Isoleucine 36 in the hydrophobic patch. Taken together these results showed a preponderant shift towards half vaults of one specific mutant in the hydrophobic patch. We note that all vault variants exhibited a larger percentage of half vaults than estimated from SEC-MALS analysis, including the wild-type protein. This is likely due to the lower pH used for staining in these experiments (2% (w/v) uranyl acetate pH ~ 4), which was previously shown to induce disassembly into half vaults^38^.

## Discussion

Recent methodological developments in cryo-EM analysis facilitate the study of continuous flexibility and discrete heterogeneity of large molecular assemblies^67^. In this work, we couple these advances to MD simulations to address the dynamic behaviour of the human vault particle at atomic resolution. Through cryo-EM variability analysis we reveal the full vault shell displays heterogeneous conformational behaviour, predominantly characterized by fluctuations in the waist diameter and swing-like opening of the half vaults. These movements result in deviation from the D39-point symmetry, long thought as the defining characteristic of the vault. By aggressive particle classification we were able to reconstruct the structure of the symmetric and a representative asymmetric conformation. Comparison of the two vault structures in combination with contact frequency analysis in MD simulations identified two distinct sets of polar interactions and a hydrophobic patch between R1 domains as the main source of the vault particle conformational plasticity.

The committed conformation displayed greater conformational heterogeneity, as reflected by its lower overall resolution. This is further supported by principal component analysis (PCA) of the particle ensemble (Supplementary Figs. 5 and 6) that showed that the modes of stretching and decrease in density at the waist are both recapitulated in the map of the committed. Crucially, the stability of both conformations during the MD simulations aligns with their coexistence in the experimental ensemble. Both states exhibited intrinsic flexibility. PCA from MD simulations captured the above-mentioned breathing motions (Supplementary Fig. 20). The committed conformation displayed higher root-mean-square deviation (RMSD) and greater residue-level flexibility (RMSF) in the waist region at the rupture, suggesting that the committed state is a continuum of flexible conformations rather than a discrete endpoint. Taken together, these observations highlight that the primed and committed states coexist in dynamic equilibrium, with the committed ensemble capturing the structural flexibility of the vault particle.

The similarity of the conformations solved in our study to those initially observed in the murine vault, albeit at lower resolution, point to the generality of the observed conformational behaviour. Furthermore, the formation of indentations, particularly at the waist region, has also been directly imaged in cells^68^, suggesting that symmetry breaking as observed here carries physiological relevance. However, it is important to note that while MVP alone constitutes the core molecular architecture of the vault particle, the vault’s additional components (TEP1, vPARP, and vault RNAs) may significantly affect its conformational landscape in vivo. Accordingly, the conformational heterogeneity and dynamics quantified here should be interpreted as an MVP-intrinsic baseline rather than a complete description of native vault behaviour. Future work reconstituting the complete complex will be critical to fully capture vault dynamics under physiological conditions with bound cargo.

The proportion of primed and committed conformations may be influenced by the particle’s intrinsic dynamics and environmental factors. The vault shell undergoes continuous breathing motions that enable sampling of multiple conformations and likely favour the more flexible committed state. The committed conformation was previously proposed to represent an on-path intermediate in the disassembly of the full vault into half vaults due to prominent structural alterations in some R1-R1 interactions. However, our mutational analysis indicated that disrupting specific hydrophobic interactions at the waist was required to destabilize the full vault and dramatically drive the formation of half vaults. Further studies will be required to fully elucidate the factors controlling the conformational space of vaults. In particular, it may be interesting to explore how the ratio of the primed and committed states is influenced by environmental factors such as temperature, salt concentration and pH, which were previously noted to have a profound effect on the stability of the vault^37,39^.

Alternatively, the committed form might not be consigned for disassembly but simply represents an ensemble of heterogeneous conformations that the vault assumes in solution. Both cryo-EM and MD simulations demonstrated that the vault is highly dynamic as a result of a large network of weak interactions than can break and form due to thermal fluctuations, resulting in the opening of further pores on the interfaces. While the physiological relevance of such transient openings remains to be established, they suggest a plausible route for limited exchange of solvent and small molecules between the lumen and the surrounding solution. Interestingly, a recent structure of the human vault in complex with vPARP has shown the presence of NAD + , a substrate of vPARP, bound within the vault lumen, consistent with the idea that vault-associated components may require solvent access for activity^69^.

The conformation behaviour and disassembly process of human capsid protein assemblies involved in cellular and inter-cellular transport, such as ferritin^70^ or the activity-regulated cytoskeleton-associated (arc) protein^71^, were shown to have profound implications for their physiological role as carriers. Interestingly, vault particles in *D. discoideum* have been observed to carry 80S ribosomes in specific orientations while interacting with ER membranes via their shoulder regions^35^. The observation of full vaults at these sites, rather than half vault structures typical of other SPFH proteins, points toward a distinct in vivo encapsulation mechanism. Therefore, we believe our insights into the vault’s inherent structural plasticity and molecular determinants underlying its assembly will be crucial for deciphering its function. Finally, our results provide guidelines for designing vault particles with optimum characteristics for encapsulation and delivery applications.

## Methods

### MVP expression and purification

The human MVP gene (GenBank: BC015623.2) was cloned in the expression vector pGAPZB (Invitrogen) as already described in^55^. Briefly, the plasmid was amplified in *E. coli* strain DH5α growth in low salt LB media (10 g/L Bacto-tryptone, 5 g/L yeast extract and 5 g/L NaCl, pH 7.0) supplemented with Zeocin (50 µg/mL), purified with silica spin-columns (Qiagen) and linearized with EcoRI and KpnII (NEB). The PEP4 protease deficient strain of *K. phaffii* SMD1168 (his4, ura3, pep4:URA3), growth for 24 hours in YPD media (20 g/L Bacto-peptone, 10 g/L yeast extract, pH 7.0) was electroporated using a Gene Pulser II electroporator (Bio-Rad) with a single pulse at 1.5 kV, 400 Ω and 25 μF and plated on YPD agar plates containing 1 M sorbitol and 100 µg/mL zeocin. Single colonies were further screened by colony-PCR amplification with Taq polymerase (ThermoFisher Scientific) and by western blot, developed with anti-MVP antibody produced in rabbit (SAB5700906 – Sigma-Aldrich).

Selected clones were inoculated in YPD supplemented with zeocin (100 µg/mL), growth at 30 °C for 24 hours and stored at -80 °C, in presence of 25% v/v glycerol. The stocks were thawed and grown in YPD at 30 °C for 72 hours in agitation, harvested, washed twice with 50 mM Tris-HCl, 150 mM NaCl, pH 8.0 in presence of 3 mM of dithiothreitol (DTT) and then lysed. Previous addition of 1 mL of lysis buffer per gram of cellular pellet (50 mM Tris-HCl, 150 mM NaCl, 1 mM TCEP, pH 8.0), plus 1 U/mL of DNase I (VWR), 1 U/mL RNase T1 (ThermoFisher Scientific), 1 U/mL RNase A (Sigma), 1 mM ATP, 1 mM PMSF, protein inhibitor cocktail (Roche), 1 mM EDTA and 5 v/v glycerol, the lysis was performed on ice by alternate cycles of vigorous shaking in presence of 25 % w/v of glass beads and centrifugation at 10,000 x *g*. After removal of the insoluble fraction by centrifugation at 20,000 x *g* for 1 hour at 10 °C, the lysate was loaded onto a cushioned discontinuous density gradient of sucrose composed of three levels (20% w/v, 45% w/v and 60% w/v) and applied to ultracentrifugation at 100,000 x *g* for 2 hours at 10 °C. The sample was recovered from the 45% level, diluted to a final concentration of sucrose below 10% and incubated with 1 U/mL RNase T, 1 U/mL RNase A, 1 mM ATP and 1 mM CaCl_2_ for 30 minutes at room temperature and centrifuged again at 16,000 x *g* to remove aggregates.

The sample was then loaded into a Sephacryl S-500 HR column 26/60 (Cytiva) previously equilibrated with SEC buffer (25 mM HEPES, 150 mM NaCl, 1 mM TCEP, pH 7.5), and eluted at 2.4 mL/min (elution peak between 140 mL and 180 mL). The fractions were analysed by SDS-PAGE stained with colloidal Coomassie InstantBlue (abcam) and by transferring the sample on a PVDF membrane, incubated with anti-MVP diluted 1:500 (SAB5700906 – Sigma-Aldrich), and developed with mouse anti-rabbit IgG-HRP (Abcam – ab6721) diluted 1:2000, followed by chemiluminescence imaging (UVITEC). Finally, the sample was concentrated with ultrafiltration centrifugal devices with 100 kDa cut-off membrane (Millipore); the protein concentration was estimated spectrophotometrically using the theoretical extinction coefficient at 280 nm of 5×10^6^ × M^−1^ × cm^−1^ obtained from Expasy servers^72^.

Prior integration in the yeast, the mutants of hMVP were prepared by PCR amplification of the original hMVP gene using the repliQa polymerase (Quantabio) and the following primers:

R_MVP_Q38A-N40A: ACCCTCTCAgcGTCCgcCCGGATGTAGGTCTTTGGCCCGACCTCC

F_MVP_Q38A-N40A: ACATCCGGgcGGACgcTGAGAGGGTACTGTTTGCCCCCATGCGC

R_MVP_D39A-R42A: ACCgcCTCATTGgCCTGCCGGATGTAGGTCTTTGGCCCGACCTCC

F_MVP_D39A-R42A: ACATCCGGCAGGcCAATGAGgcGGTACTGTTTGCCCCCATGCGC

F_MVP_I36D:ACgaCCGGCAGGACAATGAGAGGGTACTGTTTGCCCCCATGCGC

R_MVP_I36D:ACCCTCTCATTGTCCTGCCGGtcGTAGGTCTTTGGCCCGACCTCC

In order to assess the presence of alternative assembly states of the vault for negative-stain TEM and retain all assembly states of the particles, the purification of hMVP wild type and mutants subjected to negative stain TEM was performed with an ultracentrifugation passage at 100,000 x *g* for 2 hours at 10 °C without sucrose gradient, the pellet was then resuspended in SEC buffer and subjected to SEC as described above.

### Protein characterization

#### Size-exclusion chromatography coupled to multi-angle light scattering (SEC-MALS)

SEC-MALS was used to calculate the molecular mass of the sample. The Dawn Heleos II MALS detector (Wyatt), coupled to a HPLC system Alliance e2695 (Waters) was used to read the intensity of the scattered light using 8 angular detectors. The sample was filtered through 0.2 μm centrifugal filter units (Millipore), injected into a Shodex KW405-4F column (Shodex) and eluted at 0.33 mL/min in SEC buffer. The molecular weight of the eluted peak was calculated integrating the signal of refractive index detector RI-501 (Shodex) and the MALS detector with the software ASTRA (version 7.0) (Wyatt).

#### Small-angle X-ray scattering (SAXS)

SAXS data were collected at the European Synchrotron Radiation Facility (ESRF, Grenoble, France) on the BM29 BioSAXS beamline and at the P12 beamline^73^ at the PETRA-III synchrotron (DESY, Hamburg, Germany). On the BM29 beamline, scattered X-rays at a wavelength of 0.992 Å (E = 12.5 keV) were recorded using a PILATUS3 2 M (Dectris) detector in the SAXS region (q = 0.025–6 nm⁻¹). Two samples at a concentration of 0.5 mg/mL and 0.3 mg/mL were measured in a buffer containing 25 mM HEPES, 150 mM NaCl, and 1 mM TCEP (pH 7.5). For each sample measurement, 10 X-ray scattering measurements with 1 s exposure times were collected. On the P12 beamline, X-ray wavelength was 1.2397 Å (E = 10.0 keV) and Pilatus 6 M detector was employed for acquisitions in the scattering vector region (q = 0.020–4.5 nm⁻¹). Here, to evaluate concentration effects a concentration series of sample at 2.6 mg/ml, 1.3 mg/ml and 0.65 mg/ml was measured. For each sample measurement, 20 scattering frames with 0.1 s exposure were collected. At both beamlines, samples were centrifuged at 20,000 × *g* for 10 minutes at 4 °C prior to analysis. Diluted samples were injected directly using the sample changer at 20 °C. Signal frames were averaged, and buffer scattering was subtracted from the sample data.

Pair distance distribution functions were calculated with the GNOM program that is part of the ATSAS program suite^74^ for small-angle scattering data analysis (version 3.0.4). Fitting of single atomic models to the SAXS scattering curve was performed with PepsiSAXS^75^ (version 3.0), which employs an adaptive multipole expansion approach to efficiently and accurately compute scattering intensities.

### Cryo-EM sample preparation and imaging

The sample was concentrated to 250 nM and biotinylated with 10 molar excess of biotinylation reagent (NHS-PEG_12_-biotin) for 30 minutes at room temperature. The reaction was quenched with 1 mM Tris HCl pH 7.5 and excess of unbound reagent was discarded by 200-fold dilution with sample buffer (25 mM HEPES, 150 mM NaCl, 5 mM CaCl_2_, 1 mM TCEP, pH 7.5) followed by ultra-filtration, final sample concentration was estimated to ~ 50 nM. Gold film grids functionalized with 2D streptavidin crystals (SAG), prepared and stored according to the procedure already described in^76,77^ were rehydrated, equilibrated with sample buffer and incubated in sealed plate at 4 °C with 5 µL droplets of biotinylated samples. After 15 min, wells were unsealed, and samples were homogenized by pipetting in-and-out 2 µL. After further 15 min, Grids were lifted up from plate pedestals with 100 µL sample buffer, recovered, further washed on 100 µL sample buffer, plunged frozen using a Vitrobot Mark IV (ThermoFisher Scientific) with 5 seconds blot time and kept in liquid nitrogen until imaging.

Data collection was performed on a Titan Krios microscope (ThermoFisher Scientific) at 300 keV, equipped with a CS-corrector, Gatan K3 electron detector and a GIF bioquantum energy filter, with calibrated pixel size of 0.8416 Å × pix^−1^, spherical aberration of 0.01 mm and electron dose rate of 50.15 e^−^/Å^2^.

### Cryo-EM data analysis

A dataset of 6,895 movies was recorded and initially processed in RELION^78^ (version 3.1). The streptavidin crystal lattice of the SAG was subtracted from the micrographs in Fourier space with a python script already described in^77^ and available at https://github.com/NilsMarechal/SAGsub. Subtracted micrographs were then imported and further processed into cryoSPARC^58^ (version 4.5.3) (Structura Biotechnology). Contrast transfer function (CTF) was estimated for all the micrographs and used to exclude the ones with CTF > 7 Å. A selection of manually picked particles (~250 particles) was used to generate 2D classes for template-based picking. To remove junk particles, contamination and artifacts, 63,814 particles (box size of 1024 × 1024 pixels at pixel size of 0.8416 Å) were selected for 2D classification followed by ab initio 3D reconstruction. This allowed to differentiate picked particles between vaults and half vaults. Rounds of 2D classification, heterogeneous refinement, 3D classification and homogeneous refinement were employed to further curate the stack of particles and remove partially disassembled vaults artifacts using down-sampled particles in Fourier space to 550 × 550 pixels box size. An initial homogeneous refinement at GS-FSC resolution of 4.28 Å was used to perform 3D classification of the whole vault. Filter resolution at 10 Å was applied to classify the particle stack into the two conformations discussed in our work: primed and committed. Non-uniform refinement was performed after re-extracting the particles with 1024 × 1024 pixels box size for both stacks with CTF corrections (beam tilt and trefoil), minimizing per-particle defocus and scale, with 5 extra passages after GS-FSC resolution stopped improving. The final map for the primed conformation at GS-FSC resolution of 3.09 Å was obtained imposing dihedral 39-fold symmetry (D39). Applying no symmetry restrain to the refinement (C1) resulted in a similar map, lacking symmetry-mismatched components, albeit with an overall lower resolution of 4.78 Å. D39 symmetry relaxation without pose marginalization was used to refine the map of the committed conformation at GS-FSC resolution of 4.45 Å; a comparable resolution of 4.51 Å was obtained by refining the map in the absence of any imposed symmetry (C1). As the former map resulted in more legible density at the waist this was selected for the atomic reconstruction. To improve the resolution of the map in the regions of lower density, local refinements were performed with masks generated around the waist. The maps were sharpened with the negative B-factor (Å^2^) estimated after the refinement and all GS-FSC resolutions were determined using the gold-standard FSC at 0.143, with FSC curves adjusted for a tight mask in cryoSPARC^58^ (version 4.5.3). All the diagnostics plots were generated in cryoSPARC^58^ (version 4.5.3) (Structura Biotechnology).

Principal Component Analysis (PCA) of the main dataset of 35,429 particles (prior to 3D classification) was performed with CryoDRGN^57^ (version 3.4.1), which generated a projection of the latent space obtained after training an 8-dimensional latent variable model. Training was performed using an image size of 256 ×256 pixels with original box size of 550 pixels and size of 0.8416 Å/pixel, after filtering for z-score >6.

### Negative-stain transmission electron microscopy

The samples at 0.5 mg/ml were diluted 1:3 in (25 mM HEPES, 150 mM NaCl, 5 mM CaCl2, 1 mM TCEP, pH 7.5) immediately prior to grid preparation. Continuous carbon support films on 300-mesh copper grids were used. Grids were glow-discharged immediately before use. Aliquots (3 µL) of the diluted sample were applied to glow-discharged continuous-carbon grids and incubated for 1.0 min at room temperature. Excess liquid was removed by blotting with filter paper. Grids were then stained by placing them on 30 µL drops of 2% (w/v) uranyl acetate for 10 s, blotting, and repeating the 30 µL/10 s stain once more. After the second blot, grids were air-dried for 5–10 min before imaging. Imaging was performed on an Tecnai F20 transmission electron microscope (FEI). Images were acquired at nominal magnification 50,000×, corresponding to a sampling of 2.036 Å per pixel on the detector. Montaged acquisitions were collected as 5 × 5 tiles (25 micrographs per file). For each sample, ~30 montage files were recorded (~750 micrographs per sample). Imaging was performed under standard low-dose conditions at room temperature; microscope alignment and focus were optimized prior to data collection. Micrographs were imported on cryoSPARC (version 4.5.3), CTF corrected, and particles were picked in base of manually selected references. Particle alignment and 2D class averages was performed prior quantification.

### Model building

The molecular model of the vault in primed conformation was built starting from the structure of a MVP monomer (residues 1-814) generated by homology to the structure of crystallized MVP (PDB: 4HL8)^10^ using MODELLER^79^ (10.5). Coot^80^ (0.8.9) and ChimeraX^81^ (version 1.8) were employed to rebuild missing loops and to perform initial fitting to the main map taking in consideration favoured Ramachandran dihedral angles, rotamers and clashes. The map resulting from the local refinement of the waist was used to build the N-terminal domains R1-R2. Non-crystallographic symmetry of the whole map was identified and applied to reconstruct an initial model of the full 78-mer assembly using PHENIX^82^ (1.21). Cycles of improvement of the fit between the map and the atomic model of a pentameric unit of MVP were performed using ISOLDE^64^ in ChimeraX^81^ (version 1.8). Symmetry expansion, according to the D39 point group symmetry of the central MVP monomer of the pentamer, and subsequent real-space refinement of the 78-mer MVP, was performed using PHENIX^82^ (version 1.21).

The model of the vault in committed conformation was obtained through cycles of improvement of the fit between the atomic structure of the vault in primed conformation with the maps of the vault in committed conformation using PHENIX real-space refinement and Molecular Dynamics Flexible Fitting (MDFF) in ISOLDE^64^. For MDFF, the weighting between the map and the model parameters was carefully adjusted to avoid overfitting. Initial energy minimization was followed by a two-step molecular dynamics procedure; a first short step at low weighting (0.1) followed by a second step at higher weighting (0.3), for the whole all-atom model and subsequently only for the chains in the symmetry-mismatched component (using the map resulting from the local refinement of the waist). The full-length models were used to perform the molecular dynamics simulations. Afterwards, the following segments (residues 1-2, 429-449, 608-619), where the low resolution of the map prevented an accurate modelling, were trimmed from both models, which were thereby subjected to 20 macro-cycles of global real-space refinement^83^ in PHENIX (version 1.21).

### Molecular Dynamics

We performed all-atom and coarse-grained MD simulations of vault particles and half vault particles in primed and committed conformations. In all cases, we started from the atomistic models of the corresponding high-resolution cryo-EM structures solved in this work. All the MD simulations reported in this work were performed in GROMACS 2024^84^ and are listed in Supplementary Table 3. The MD simulation setup, MD trajectory analysis and visualization are described below.

#### All-atom MD simulations

The atomistic structures of vault and half-vault particles were used as input for all-atom MD simulations. The AMBER99SB-ILDN^85^ force field was applied, and the systems were solvated with TIP3P water and 150 mM NaCl. The proteins were protonated at pH 7.0. Energy minimization was performed using the steepest-descent algorithm until the maximum force was <1000 kJ·mol⁻¹·nm⁻¹. Equilibration proceeded in multiple stages with position restraints (force constant: 1000 kJ·mol⁻¹·nm⁻²) applied to protein heavy atoms. All simulations employed the Verlet neighbour search algorithm (neighbour list cutoff: 1.1 nm, update frequency: 20 timesteps). Lennard-Jones interactions were truncated at 1.1 nm. Long range electrostatics were treated with Particle Mesh Ewald and truncated at 1.1 nm. (i) NVT equilibration for 375 ps with a 1 fs timestep, using the Berendsen thermostat^86^ (T = 310 K, τ = 0.01 ps). (ii) NPT equilibration for 1 ns with a 2 fs timestep, using isotropic pressure coupling via the Berendsen barostat^86^ (P = 1 bar, τ = 2 ps) and switching to the v-rescale thermostat^87^ (T = 310 K, τ = 0.1 ps). (iii) NPT equilibration for 100 ns with a 2 fs timestep, using the v-rescale thermostat (T = 310 K, τ = 0.1 ps) and the c-rescale barostat^88^ (P = 1 bar, τ = 2 ps). (iv) Final NPT equilibration for 20 ns under the same conditions as (iii) but with the position restraint force constant reduced to 10 kJ·mol⁻¹·nm⁻². Due to the restrictive computational cost of large-scale systems ( > 18 million atoms), two production runs of 0.50 μs per initial structure were performed following the protocol in (iii), without position restraints on protein heavy atoms.

#### Coarse-grained MD simulations

The atomistic structures of vault and half-vault particles were used as input to generate coarse-grained (CG) Martini protein models. Each MVP chain was coarse-grained individually using the martinize.py script^89^, with default protonation states and secondary structure restraints assigned via DSSP^90^. All CG simulations employed the Martini 2.2 force field^91^ alongside the ElNeDyn2.2 protein force field^92^. Given the known overestimation of nonbonded interactions^93^ in Martini 2.2, we used an α parameter of 0.85 to scale protein-protein interactions relative to protein-solvent interactions^94^. To maintain protein stiffness, an elastic network with bond cutoffs of 0.5 and 0.9 nm was applied to each MVP chain with elastic bond force constant of 1000 kJ·mol⁻¹·nm⁻².

The systems were solvated with coarse-grained water containing 10% anti-freeze WF particles and 150 mM NaCl, with excess ions ensuring charge neutrality. Energy minimization was performed using the steepest-descent algorithm and a maximum force <1000 kJ·mol⁻¹·nm⁻^1^. Equilibration proceeded in three stages with position restraints (force constant: 1000 kJ·mol⁻¹·nm⁻²) applied to protein backbone beads: (i) NVT equilibration for 1.0 ns with a 5 fs timestep, using the Berendsen thermostat^86^ (T = 310 K, τ = 1 ps). (ii) NPT equilibration for 2.5 ns with a 5 fs timestep, applying isotropic pressure coupling via the Berendsen barostat^86^ (P = 1 bar, τ = 12 ps) and switching to the v-rescale thermostat^87^ (T = 310 K, τ = 1 ps). (iii) Final NPT equilibration for 300 ns with a 15 fs timestep, using the v-rescale thermostat (T = 310 K, τ = 1 ps) and the c-rescale barostat^88^ (P = 1 bar, τ = 12 ps). Three production runs per initial structure of 5 μs each were conducted using the same protocol as (iii) but without position restraints on backbone beads. All simulations employed the Verlet neighbour search algorithm (neighbour list cutoff: 1.4 nm, update frequency: 20 timesteps). Lennard-Jones and Coulomb interactions were truncated at 1.2 nm using the Verlet-shift potential modifier and reaction-field electrostatics. All MD simulations were performed at the AMD-based supercomputer VIPER of the Max Planck Society, operated at the Max Planck Computing and Data Facility in Garching.

Visual Molecular Dynamics (VMD)^95^ 1.9.4 was used for trajectory visualization, while MDAnalysis^96^ 2.1.0 was employed for trajectory analysis, RMSD, RMSF and diameter measurements. Frequency of residue-residue interactions between MVP chains during MD simulations was calculated with the python package prolif.

#### Internal‑volume and water/ion exchange analysis

The cavity volume of each vault model was measured with VMD’s measure volinterior command^97^. A closed surface was generated using a grid spacing of 1.8 Å, a radius‑scaling factor of 3.2, 32 rays, and an isovalue of 0.9. To seal the vault caps, a lattice of dummy particles was placed at the residues defining the opening (resid 814). Water‑oxygen, Na⁺ and Cl⁻ atoms crossing this surface were tracked to quantify exchange. An exchange event was recorded when a water molecule (or ion) moved from the exterior to the interior, or vice versa, between successive analysis frames (sampled every 1 ns).

#### Principal Component Analysis from MD simulations

PCA of the atomistic trajectories was carried out after converting the full‑atom coordinates to a coarse‑grained representation in which every five consecutive residues were merged into a single bead using MDAnalysis. The resulting bead trajectories were first aligned to the coarse‑grained reference topology with a least‑squares fit (gmx confrms) to eliminate overall translation and rotation. A covariance matrix of the bead positions was then constructed and diagonalized with gmx covar, yielding eigenvectors (the principal components) and their associated eigenvalues. Finally, the trajectories were projected onto the leading eigenvectors using gmx anaeig to visualize the dominant large-scale conformational fluctuations of the vault particle.

#### Ion permeation analysis from CG-MD simulations

To quantify the cumulative crossing of ions through specific regions in the vault shell, we analysed the coarse-grained MD trajectories of the vault particle using MDAnalysis and custom Python scripts. A region was defined by the convex hull of six neighbouring R domains. A vector **A** between the centre of mass (CoM) of the full vault shell and the CoM of the convex hull defined the main axis of a cylindrical volume. This volume, with a radius =3 nm, was used to spatially constrain the analysis to ions passing directly through the selected R domains. At each frame, we determined which ions (Na⁺ or Cl⁻) are within the cylindrical volume.

Furthermore, we define the vector **B** between the ion position and the CoM of the convex hull. For the cases where, | **A** • **B** | > 1 nm the ion was considered to be either *inside* if **A** • **B** < 0 or *outside* if **A** • **B** > 0. The condition | **A** • **B** | > 1 nm was used to account for the thickness of the vault shell and to filter out ions stuck at the vault surface. A crossing event was counted when an ion moved from the *outside* to the *inside* or vice versa between frame *n–2* and frame *n*, provided that the ion remained within the cylindrical region in both frames. Cumulative ion permeations were obtained by summing the number of inward and outward crossing events across the trajectory. The ion permeation data were recorded at each frame, with frames being recorded at 1.5 ns intervals.

### Structural analysis

Analysis of the inter-chains contacts was performed in ChimeraX^81^ (version 1.8) between residues 1-379 of 6 chains imposing a search of contact residues with at least 11 Å^2^ of buried area with a default probe radius of 1.4 Å. Atomic distances of alpha carbons between couples of residues Ala801, Gln678 and Asp39, between chains EB and YB as well as chains OB and UA were calculated in ChimeraX^81^ (1.8) and used to describe the diameter of the waist and cap-helix of the vault. Residue positional entropy-based conservation scores, calculated by AL2CO^98^.

Final validation of the structure was performed in PHENIX^82^ (version 1.21). Model resolution and Model resolution range (Å) were calculated with the average value and range of values for the local resolution at FSC threshold 0.5 at atom positions using ChimeraX^81^ (version 1.8).

### Reporting summary

Further information on research design is available in the Nature Portfolio Reporting Summary linked to this article.

## Supplementary information

Supplementary Information

Description of Additional Supplementary Files

Supplementary Movie 1

Supplementary Movie 2

Supplementary Movie 3

Reporting Summary

Transparent Peer Review file

## Source data

Source Data

## Data Availability

Cryo-EM maps are deposited in the Electron Microscopy Data Bank (EMDB) with the following accession codes EMD-53415, EMD-53423 and EMD-53440, for the vault in primed conformation, committed conformation and for the 39-mer half vault, respectively. Local refinement cryo-EM maps of the vault’s waist are deposited on EMDB with the following accession codes EMD-53438 and EMD-53439 for the vault in primed and committed conformation, respectively. Atomic structures are deposited in the Protein Data Bank (PDB) with the following accession codes 9QW9 and 9QWQ for the human vault in primed and in committed conformation, respectively. SAXS results are deposited in the Small Angle Scattering Biological Data Bank (SASDB) with accession code SASDXJ3. All-atom and coarse-grained MD trajectories for each replicate and initial configuration have been deposited in the Zenodo entry 19145360^99^. Source data are provided with this paper.
