# Supplementary material for: Structural flexibility of the human vault particle revealed by high-resolution cryo-EM and molecular dynamics simulations
Source: Nat Commun. 2026 May 2;17:6033. doi: 10.1038/s41467-026-72674-4 (PMC13347060; doi:10.1038/s41467-026-72674-4)
Supplement: Supplementary file 1 — Supplementary Information [file 41467_2026_72674_MOESM1_ESM.pdf]

Supplementary Information

Structural flexibility of the human vault particle revealed by high-resolution cryo-EM and molecular dynamics simulations

Fabio Lapenta[1,2,†,*], Karen Palacio-Rodriguez[3,†], Sergio Cruz-León[3], Simone Marrancone[1], Jana Aupič[4], Nils Marechal[5], Alexandre Durand[5], Dihia Moussaoui[6], Sonia Covaceuszach[7], Bhavani Gangupam[1], Claudia D'Ercole[1], Cristian Parra[8], Davide Cotugno[9,10], Giulia Tomaino[9], Paolo Tortora[9], Ario de Marco[1], Alberto Cassetta[7], Alessandra Magistrato[4], Gerhard Hummer[3,11]

[1] Laboratory for Environmental and Life Sciences, University of Nova Gorica, Vipavska cesta 13, 5000 Nova Gorica, Slovenia
[2] International Centre for Genetic Engineering and Biotechnology (ICGEB), Padriciano 99, 34149, Trieste, Italy
[3] Department of Theoretical Biophysics, Max Planck Institute of Biophysics, Max-von-Laue-Straße 3, 60438 Frankfurt am Main, Germany
[4] Consiglio Nazionale delle Ricerche - Istituto Officina dei Materiali c/o International School for Advanced Studies, via Bonomea 265, 34136 Trieste, Italy
[5] Institut de Génétique et de Biologie Moléculaire et Cellulaire (IGBMC), Parc D'Innovation 1 Rue Laurent Fries, 67404 Illkirch Cedex, France
[6] BM29 BIOSAXS beamline, European Synchrotron Radiation Facility (ESRF), Grenoble, France
[7] Istituto di Cristallografia, Consiglio Nazionale delle Ricerche, Strada Statale 14 km 163.5, 34149 Trieste, Italy
[8] Max Planck Tandem Group Biophysics of Tropical Diseases, Faculty of Exact and Natural Sciences, University of Antioquia, 050010 Medellín, Colombia
[9] Department of Biotechnology and Biosciences, University of Milano-Bicocca, 20126 Milano, Italy
[10] Current address: IEO, European Institute of Oncology IRCCS, Department of Experimental Oncology, 20139 Milan, Italy
[11] Institute of Biophysics, Goethe University Frankfurt, 60438 Frankfurt am Main, Germany
[†] These authors contributed equally to this work
[*] Author to whom correspondence should be addressed (e-mail: fabio.lapenta@ung.si)

Table of Contents

Supplementary Figures 1 to 27
Supplementary Tables 1 to 3
Supplementary Discussion

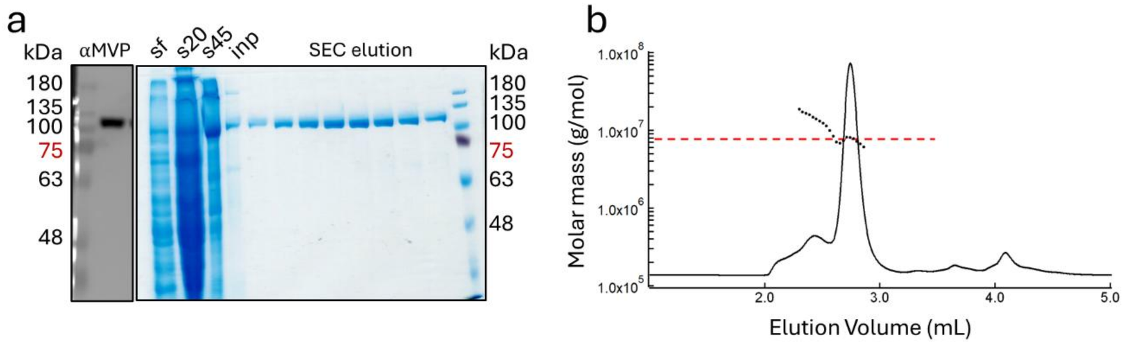

Supplementary Figure 1. **Protein characterization. a** Left: representative western blot (of three technical replica) of the protein sample, developed using anti-MVP antibody. Right: SDS-PAGE of the sample. Lanes from left to right: soluble fraction of the lysate (sf); supernatant after 20,000 x *g* centrifugation (s20); sucrose layer 45% (s45); SEC input after RNase incubation (inp); SEC fractions containing fully assembled vault. **b** SEC coupled with Multi Angle Light scattering (SEC-MALS) analysis of the sample. The main peak corresponds to 7.66 ± 0.6% MDa. The UV signal at 280 nm is shown as black line and the molecular mass, calculated with 8 angular detectors, is represented by scattered points near the main peak (red dashed line at 8.0 MDa). Source data are provided as a Source Data file.

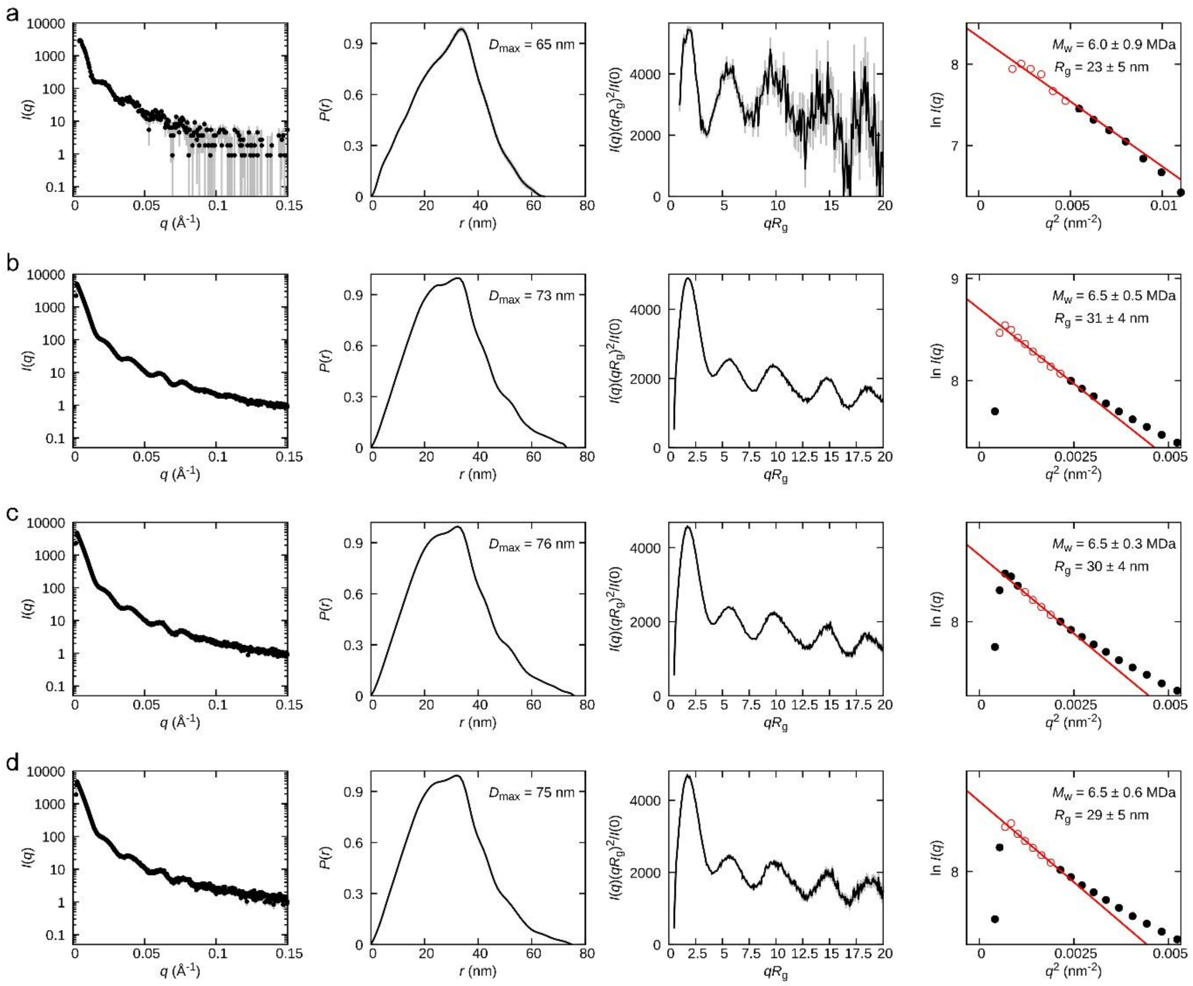

Supplementary Figure 2. **SAXS analysis of MVP particles in solution.** From left to right scattering curves, pair-distance distribution functions, Kratky plots and Guinier plots are shown for two independent SAXS samples. **a** Plots generated from a scattering curve obtained by merging two measurements carried out at concentration of 0.5 and 0.3 mg/ml at the BM29 BioSAXS beamline at ESRF, EMBL Grenoble. **b-d** Plots obtained by analyzing a concentration series ((**b**) 2.6 mg/ml, (c) 1.3 mg/ml and (**d**) 0.65 mg/ml) measured at the P12 beamline at DESY, EMBL Hamburg. Grey error bars show the S.D. for each data point. Source data are provided as a Source Data file.

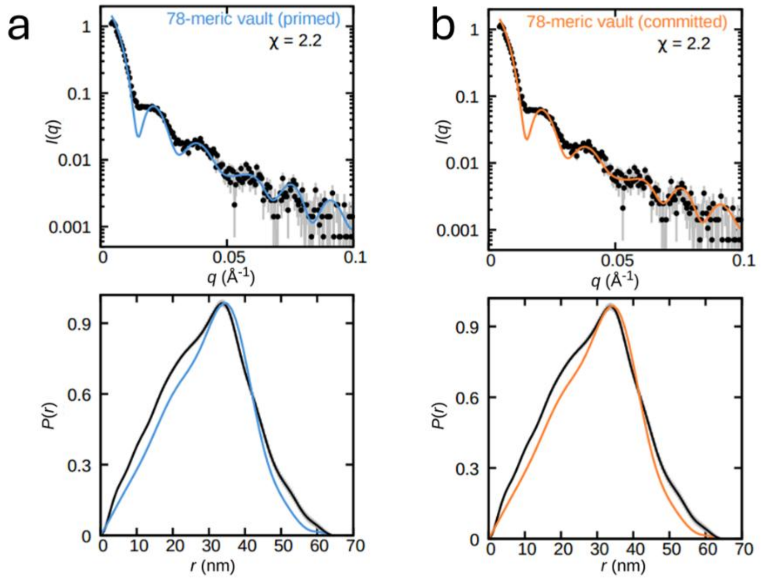

Supplementary Figure 3. **SAXS fit**. **a** Experimental SAXS scattering curve and derived distance-distribution plot in black with fits to the cryo-EM structure of the 78-mer vault in primed conformation (in blue). **b** Experimental SAXS scattering curve and derived distance-distribution plot in black with fits to the cryo-EM structure of the 78-mer vault in the committed conformation (in orange). Since the χ value, which represents the fit quality, is equal for both conformational states, SAXS cannot be used to assess the relative populations of the primed and committed conformation of the vault particle in solution. Grey error bars show the S.D. for each data point. Source data are provided as a Source Data file.

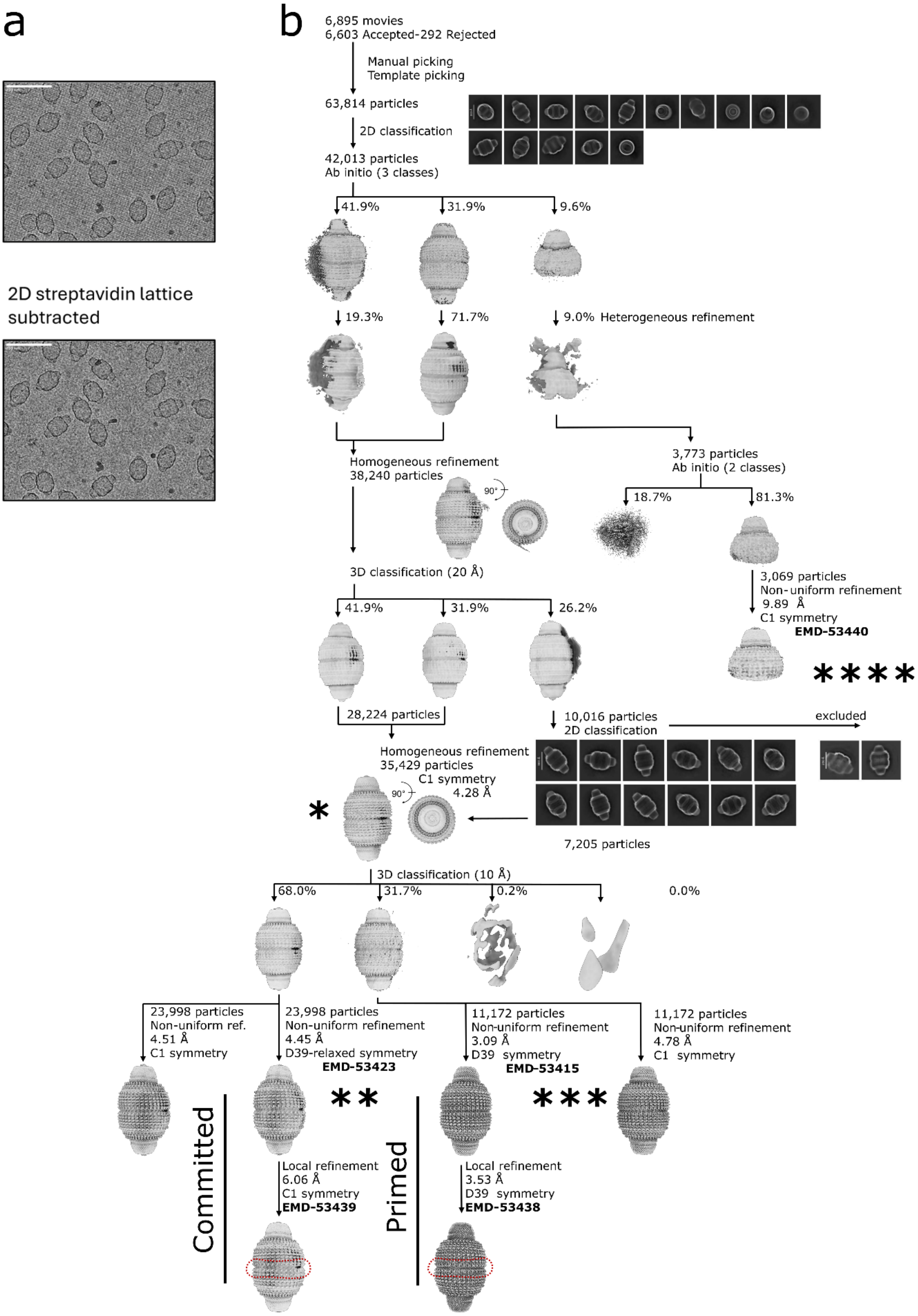
a
2D streptavidin lattice subtracted
b
6,895 movies
6,603 Accepted-292 Rejected
Manual picking
Template picking
63,814 particles
2D classification
42,013 particles
Ab initio (3 classes)
41.9%
31.9%
9.6%
19.3%
71.7%
9.0% Heterogeneous refinement
Homogeneous refinement
38,240 particles
90°
3,773 particles
Ab initio (2 classes)
18.7%
81.3%
3D classification (20 Å)
3,069 particles
Non-uniform refinement
9.89 Å
C1 symmetry
EMD-53440
41.9%
31.9%
26.2%
****
28,224 particles
10,016 particles
2D classification
excluded
Homogeneous refinement
35,429 particles
C1 symmetry
4.28 Å
*
90°
7,205 particles
3D classification (10 Å)
68.0%
31.7%
0.2%
0.0%
23,998 particles
Non-uniform ref.
4.51 Å
C1 symmetry
23,998 particles
Non-uniform refinement
4.45 Å
D39-relaxed symmetry
EMD-53423
11,172 particles
Non-uniform refinement
3.09 Å
D39 symmetry
EMD-53415
11,172 particles
Non-uniform refinement
4.78 Å
C1 symmetry
**
***
Committed
Primed
Local refinement
6.06 Å
C1 symmetry
EMD-53439
Local refinement
3.53 Å
D39 symmetry
EMD-53438

Supplementary Figure 4. **Cryo-EM data processing**. **a** Top: representative micrograph of 6,603 accepted micrographs before and after subtraction of the streptavidin 2D crystal lattice by Fourier filtering of Bragg spots (scale bar =100 nm). **b** Flowchart of data processing performed in cryoSPARC. The procedure used, particle numbers, enforced symmetry, final resolution and eventual region of the mask used for local refinement (in red dotted line) are indicated at each step in the flowchart. Unsharpened maps from the 3D refinement passages and representative 2D classes from reference-free 2D classification of particles either kept or excluded shown. Scale bars for 2D class averages = 360 Å. * initial refinement of the vault, then classified into the two conformational states. ** Committed conformation of the vault obtained with relaxed D39 symmetry. *** primed conformation of the vault obtained with D39 symmetry. **** 39-mer half-vault.

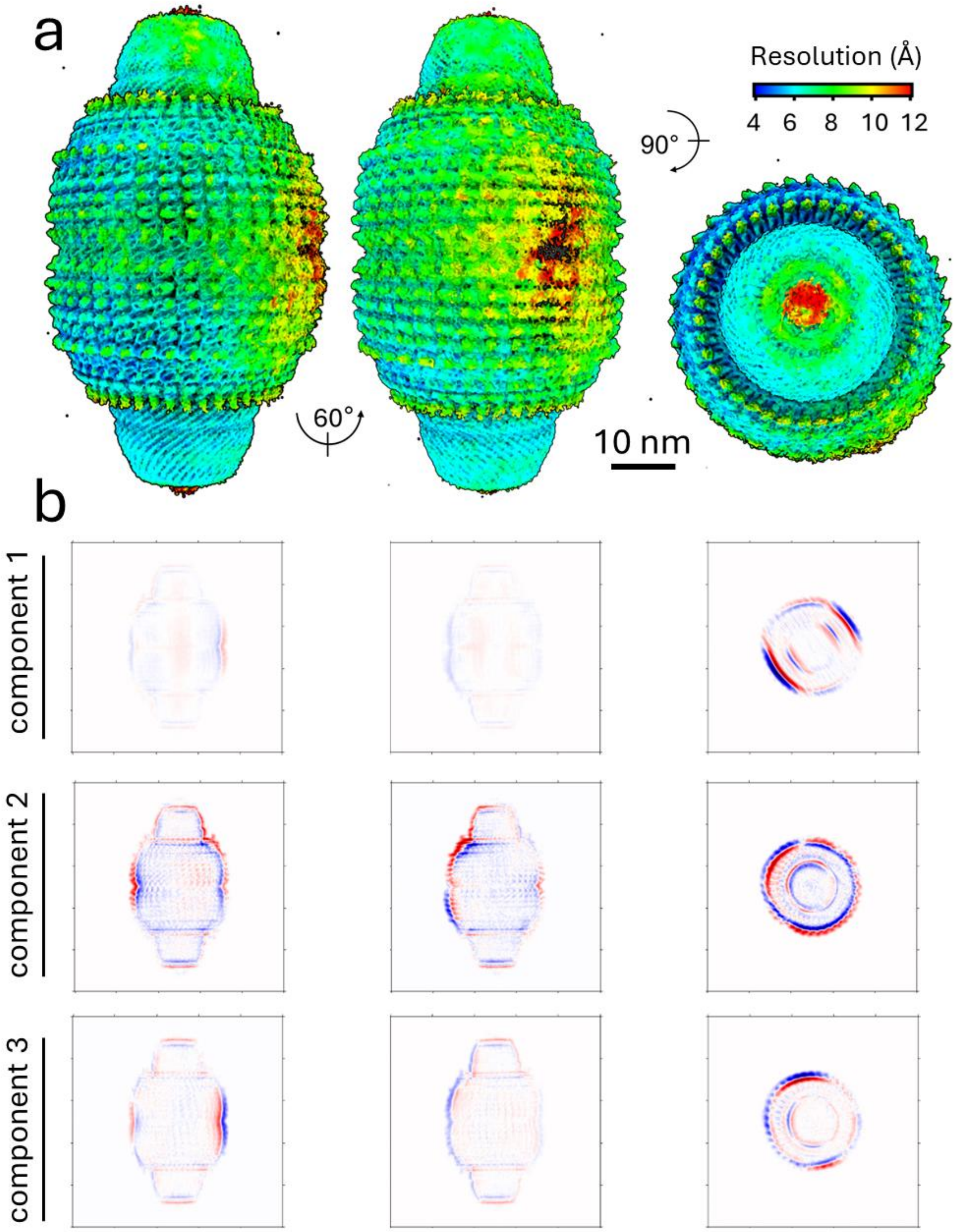

Supplementary Figure 5. **Initial reconstruction of the vault particle. a** Volume resulting from homogeneous refinement of the 35,429 particles identified as intact vaults. The local resolution of the map is shown in rainbow color scale. **b** Three orthogonal slices through each variability component of the 3D volume resulting from 3D variability analysis (3DVA) applied to the same 35,429 particles (see also Supplementary Movie 1). To explain heterogeneity within the particle stack, positive and negative values in the 3D volume at each voxel are shown in a color scale from blue to red, respectively.

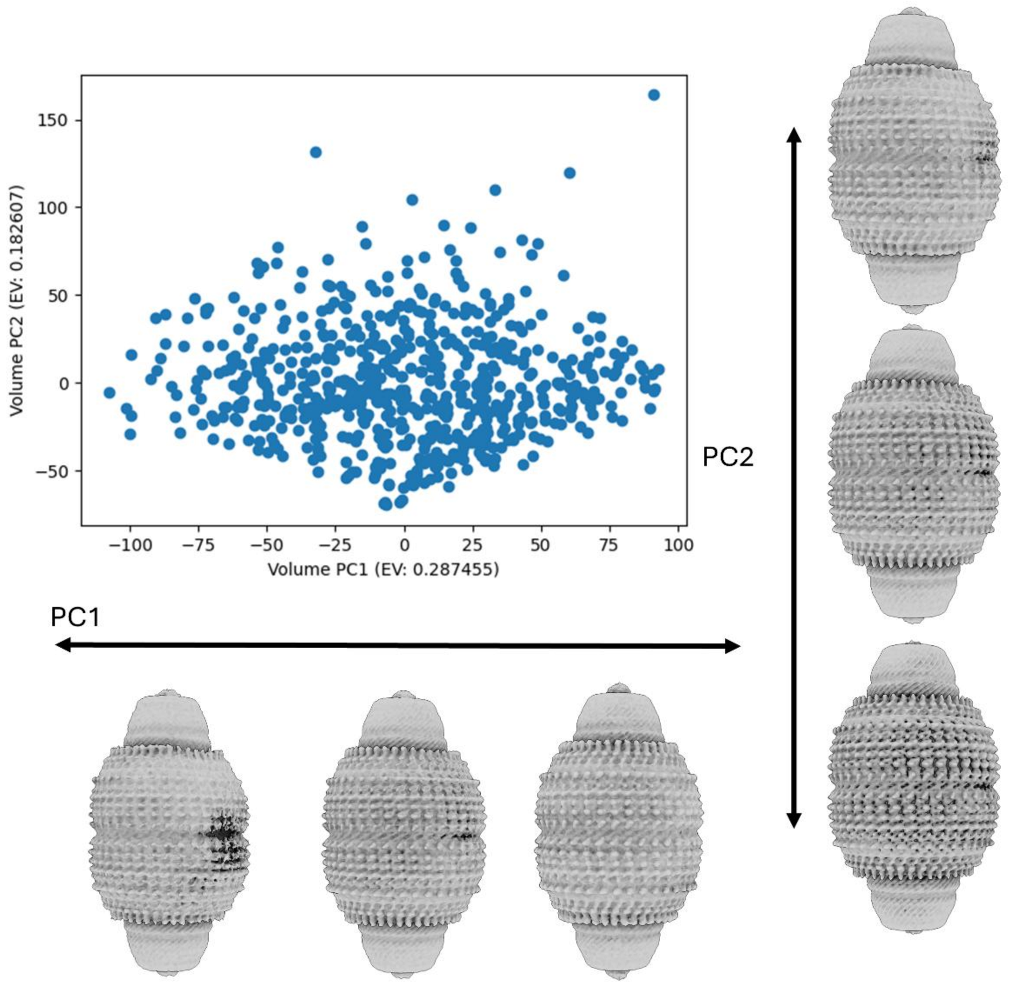

Supplementary Figure 6. **Principal Component Analysis (PCA) of the whole vault particle cryo-EM data set.** Principal Component Analysis (PCA) obtained with CryoDRGN after training an 8-dimensional latent model on the main dataset of 35,429 particles (prior to 3D classification). Representative 3D density maps are shown along the principal components (PC) of the latent space. The landscape represents the ensemble of conformations reconstructed from single-particle images. PCA on this space identified two dominant modes of conformational variability: PC1 and PC2, with explained variance (EV) ratio of 0.29 and 0.18, respectively, which indicates how much of the total conformational heterogeneity is described by each PC (or mode). Source data are provided as a Source Data file.

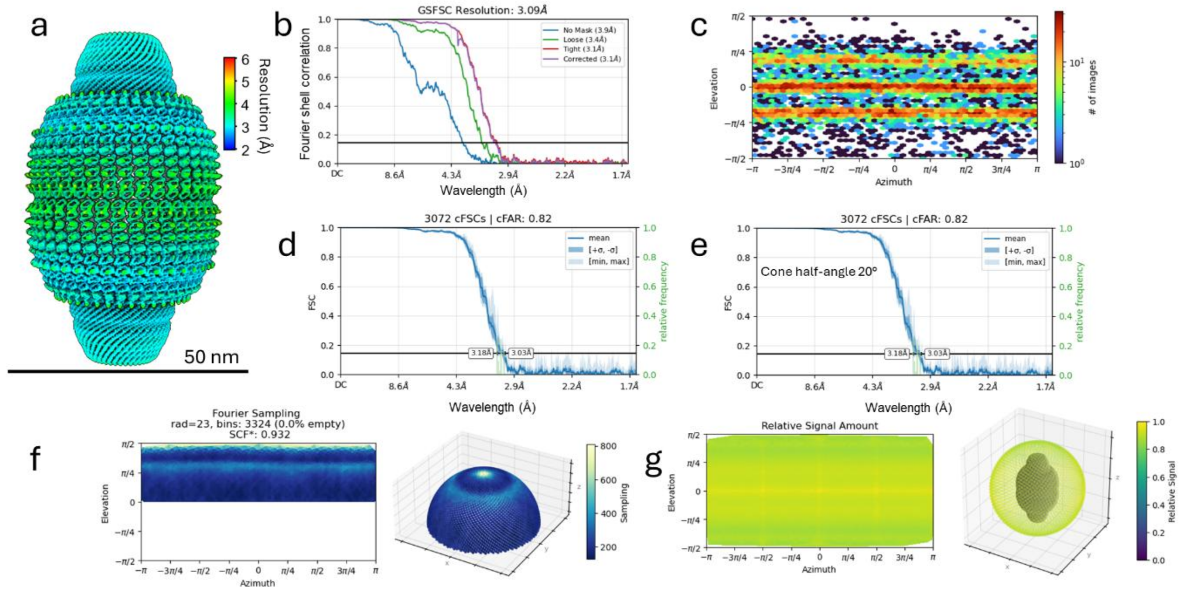

Supplementary Figure 7. **Reconstructed maps of the vault in primed conformation. a** Local resolution of the (unsharpened) map resulting from the cryo-EM reconstruction of the vault in the primed conformation, shown in rainbow color scale. **b** Corresponding gold standard Fourier shell correlation (GS-FSC) curves with crossing at 0.143 and GS-FSC resolution reported on top. **c** 2D orientation plot where cartesian coordinates of the map corresponds to two of the Euler angles and color represents the orientation distribution. **d, e** Conical Fourier shell correlation area ratio (cFAR) plots for 3072 conical masks with half-angle of either 20° (d) or 40° (e) uniformly distributed across the viewing directions (Fibonacci sampling). **f** Sampling Compensation Factor (SCF*) plot measuring the Fourier representation of the alignments of the particle set on cartesian azimuth-elevation chart and on a hemisphere. **g** Relative signal amount plots, showing how FSC curves computed within a toroidal section of the half-maps correlate on an azimuth-elevation chart, and a 3D viewing sphere encircling a low-passed (chiral) map of the vault in committed conformation. Source data are provided as a Source Data file.

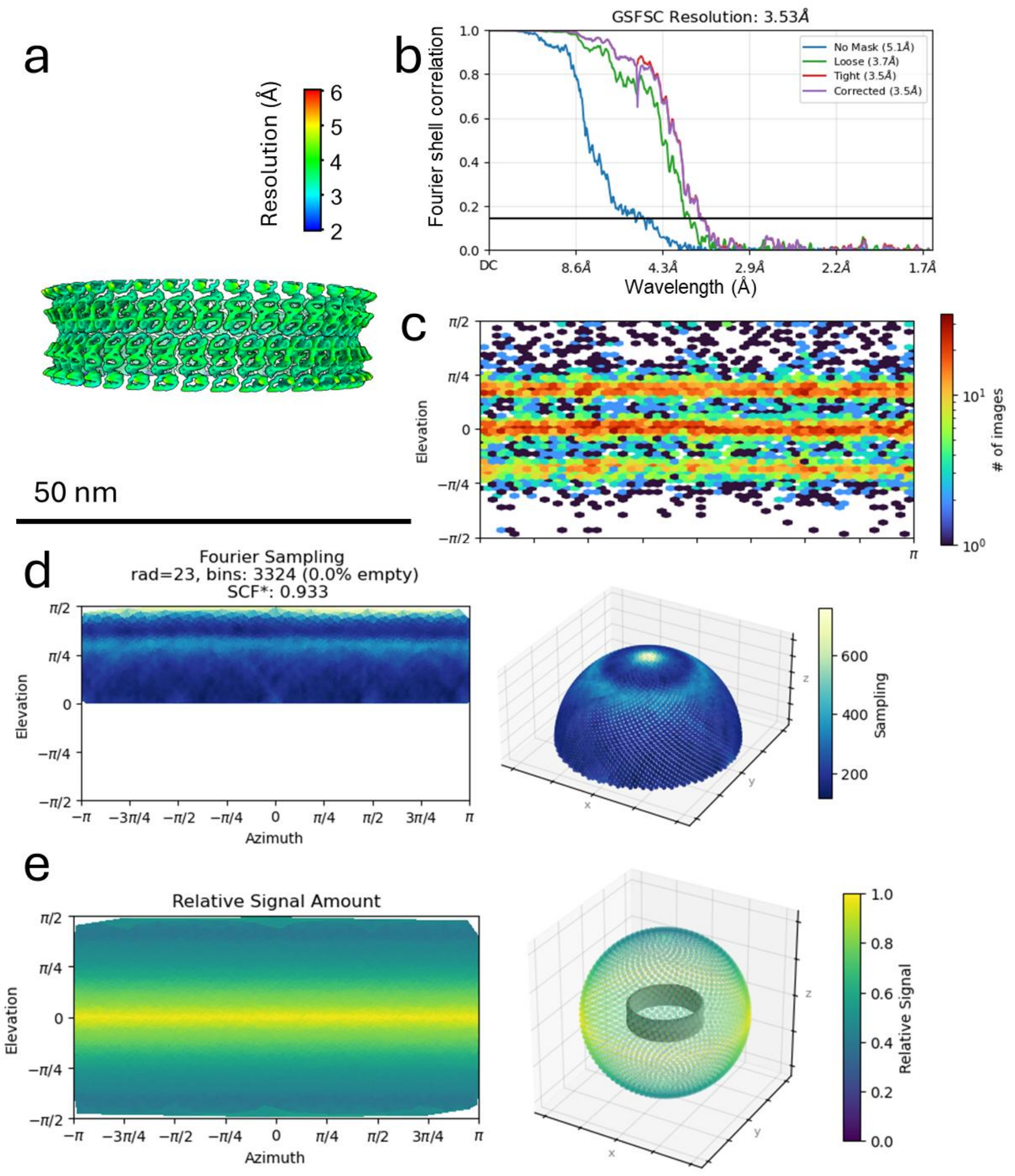

Supplementary Figure 8. **Reconstructed local maps of the vault's waist in primed conformation. a** Local resolution of the (unsharpened) map resulting from the cryo-EM reconstruction of the vault's waist in the primed conformation, shown in rainbow color scale. **b** Corresponding gold standard Fourier shell correlation (GS-FSC) curves with crossing at 0.143 and GS-FSC resolution reported on top. **c** 2D orientation plot where cartesian coordinates of the map corresponds to two of the Euler angles and color represents the orientation distribution. **d** Sampling Compensation Factor (SCF*) plot measuring the Fourier representation of the alignments of the particle set on cartesian azimuth-elevation chart and on a hemisphere. **e** Relative signal amount plots, showing how FSC curves computed within a toroidal section of the half-maps correlate on an azimuth-elevation chart, and a 3D viewing sphere encircling a low-passed (chiral) local map of the vault's waist in primed conformation. Source data are provided as a Source Data file.

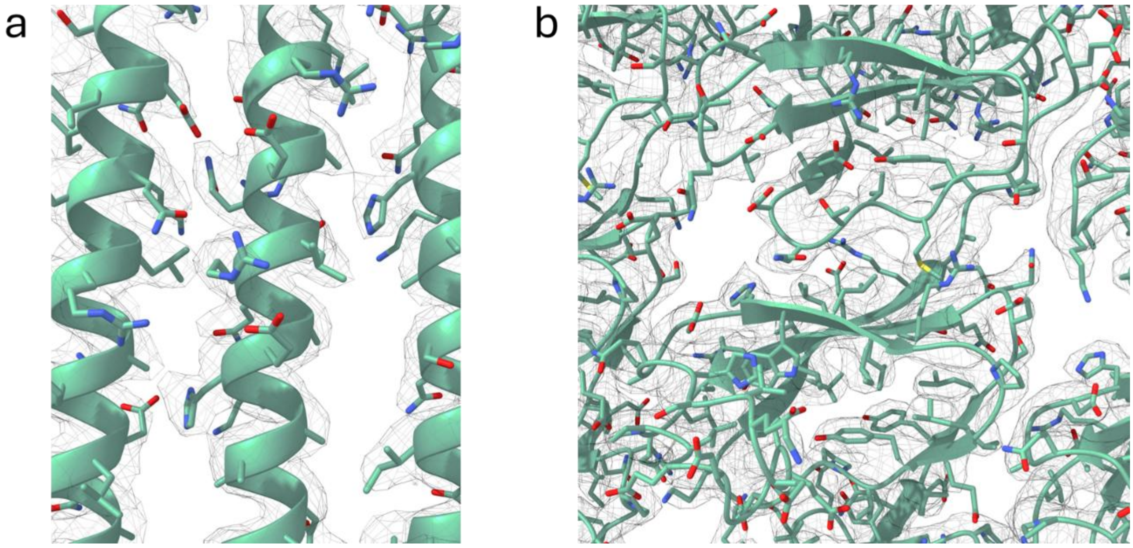

Supplementary Figure 9. **Side chain resolution of the primed vault. a** Detail of sharpened map of the cap-helix of the vault in the primed conformation (contour level 0.8). **b** Detail of the sharpened map of the repeat domains of the vault in the primed conformation (contour level 0.8).

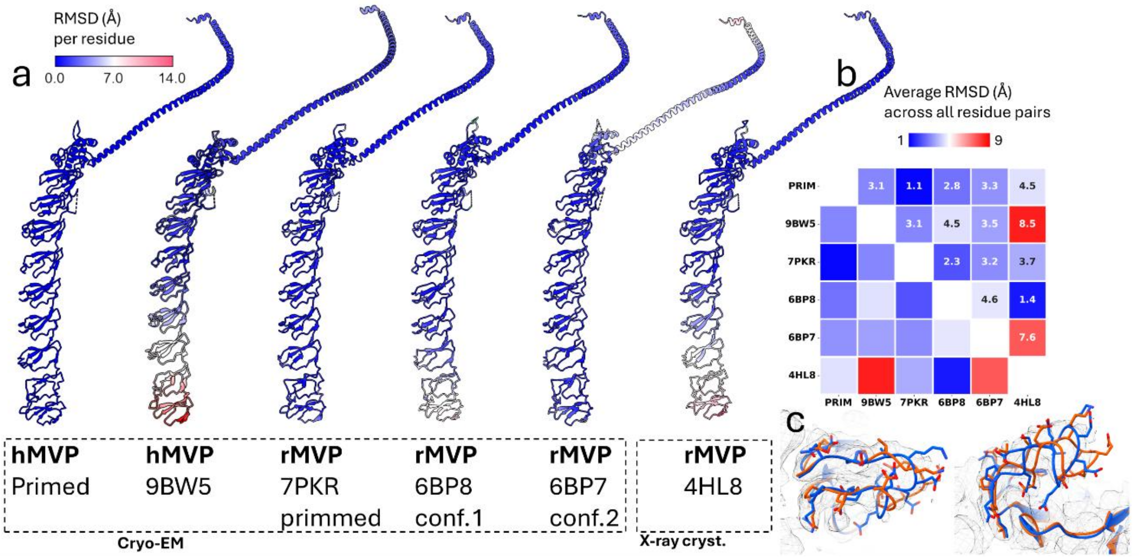

Supplementary Figure 10. **Structural comparison of the human vault to murine structures. a** root-mean-square deviation (RMSD) between atomic coordinates of the primed human vault (hMVP) and *Rattus norvegicus* vault (rMVP) structures. **b** RMSD between all atom pairs in the hMVP from this study and all published MVP structures (with respective PDB code). **c** Detailed structural comparison of the loop 342-347, showing the hMVP primed conformation (this work) in blue, overlaid with the crystal structure (PDB: 4HL8) in red. The meshed cryo-EM density map (contour level 0.8) from this work is also shown.

Supplementary Fig. 9

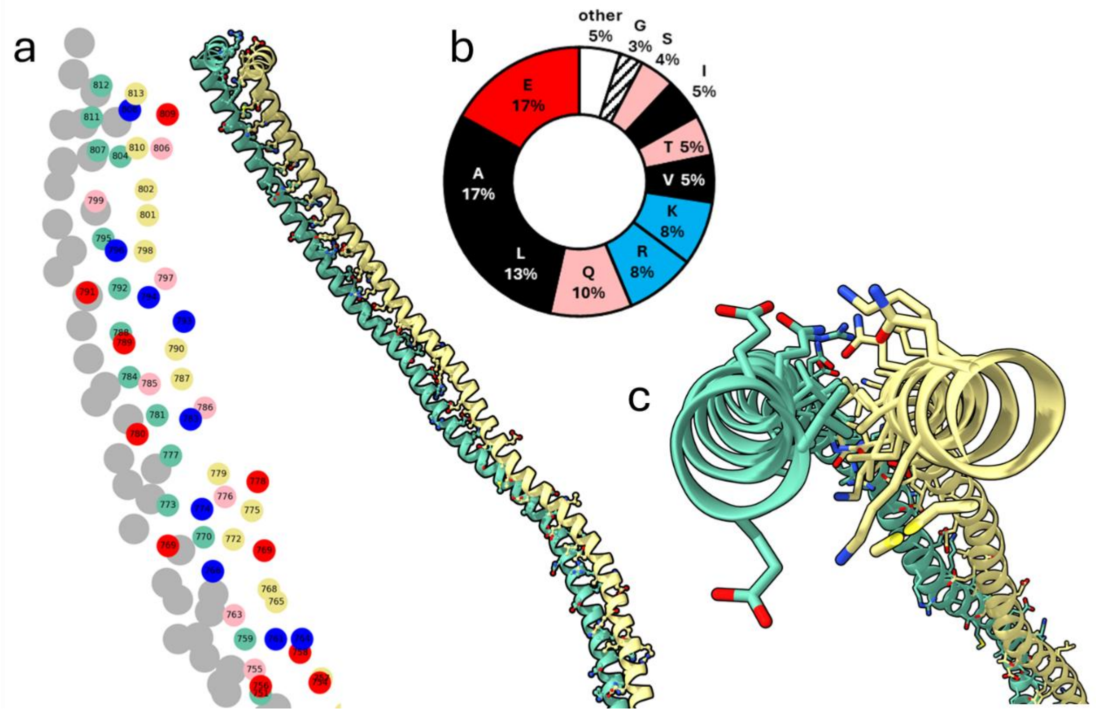

Supplementary Figure 11. **Lateral contacts in the helix-cap domain of hMVP. a** The residues' number of amino acids involved in lateral contacts between two MVP chains in circles, with blue and red indicating positively and negatively charged residues, respectively, yellow and green for other residues. The plot represents a side view of the atomic model, shown on the right. **b** Pie chart, showing the distribution of residues found in later chains MVP-MVP contacts. **c** Top view of two helices in the cap-helix, showing knobs-into-holes contacts established by the inner cleft residues between the two helices and the polar residues surrounding the hydrophobic cleft.

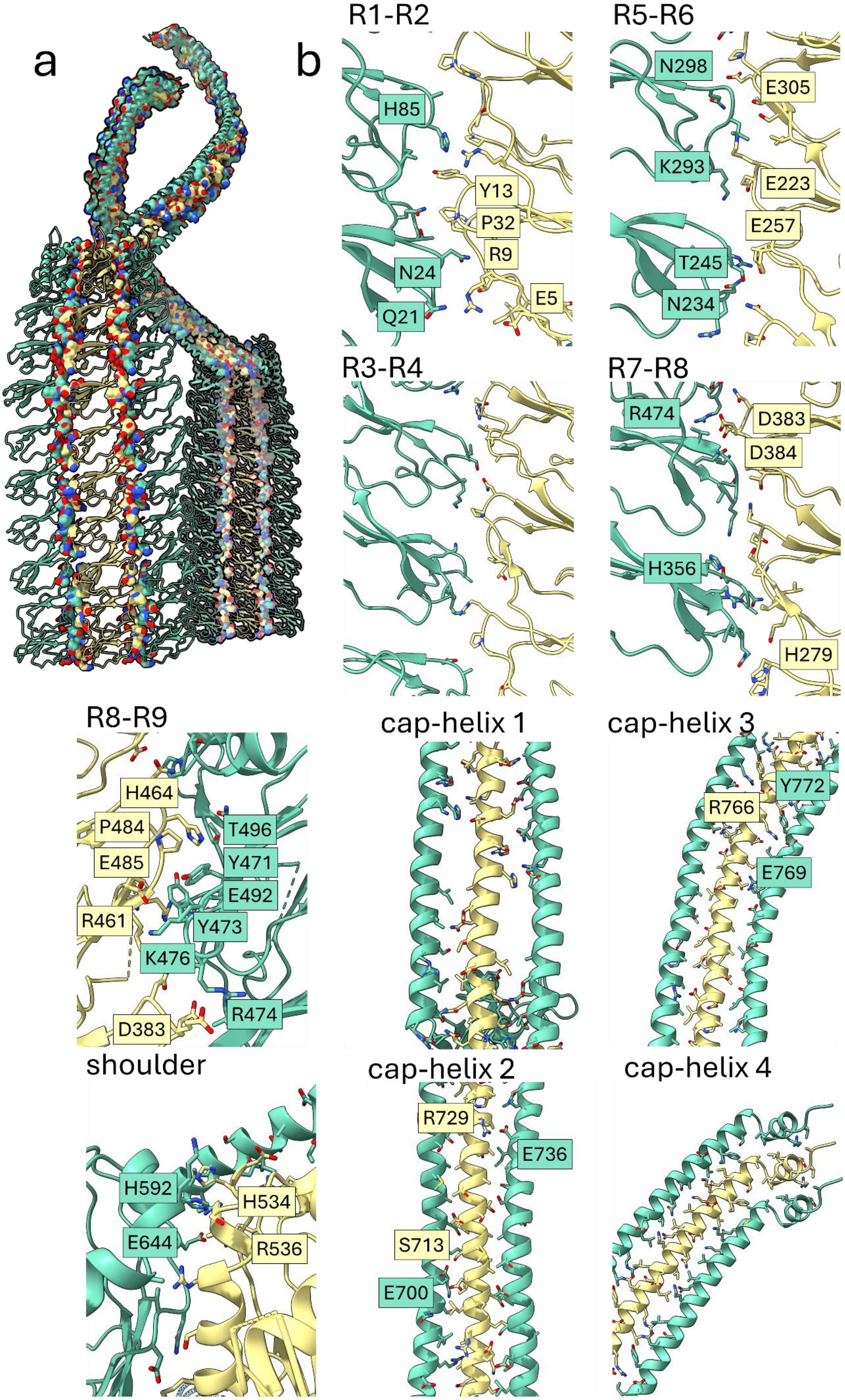

a
b
R1-R2
H85
Y13
P32
R9
N24
E5
Q21
R5-R6
N298
E305
K293
E223
E257
T245
N234
R3-R4
R7-R8
R474
D383
D384
H356
H279
R8-R9
H464
P484
T496
E485
Y471
E492
R461
Y473
K476
R474
D383
cap-helix 1
cap-helix 3
Y772
R766
E769
shoulder
H592
H534
E644
R536
cap-helix 2
R729
E736
S713
E700
cap-helix 4

Supplementary Figure 12. **Neighboring lateral contacts between MVP monomers in the primed vault. a** Cartoon representation of three MVP monomers, with surface view of residues involved in neighboring contacts between laterally interacting subunits. **b** Detailed views of different regions of the atomic model of the vault in the primed conformation, represented as cartoon. Side chains of residues involved in lateral contacts are shown as sticks. Ionizable residues involved in polar interactions are labelled.

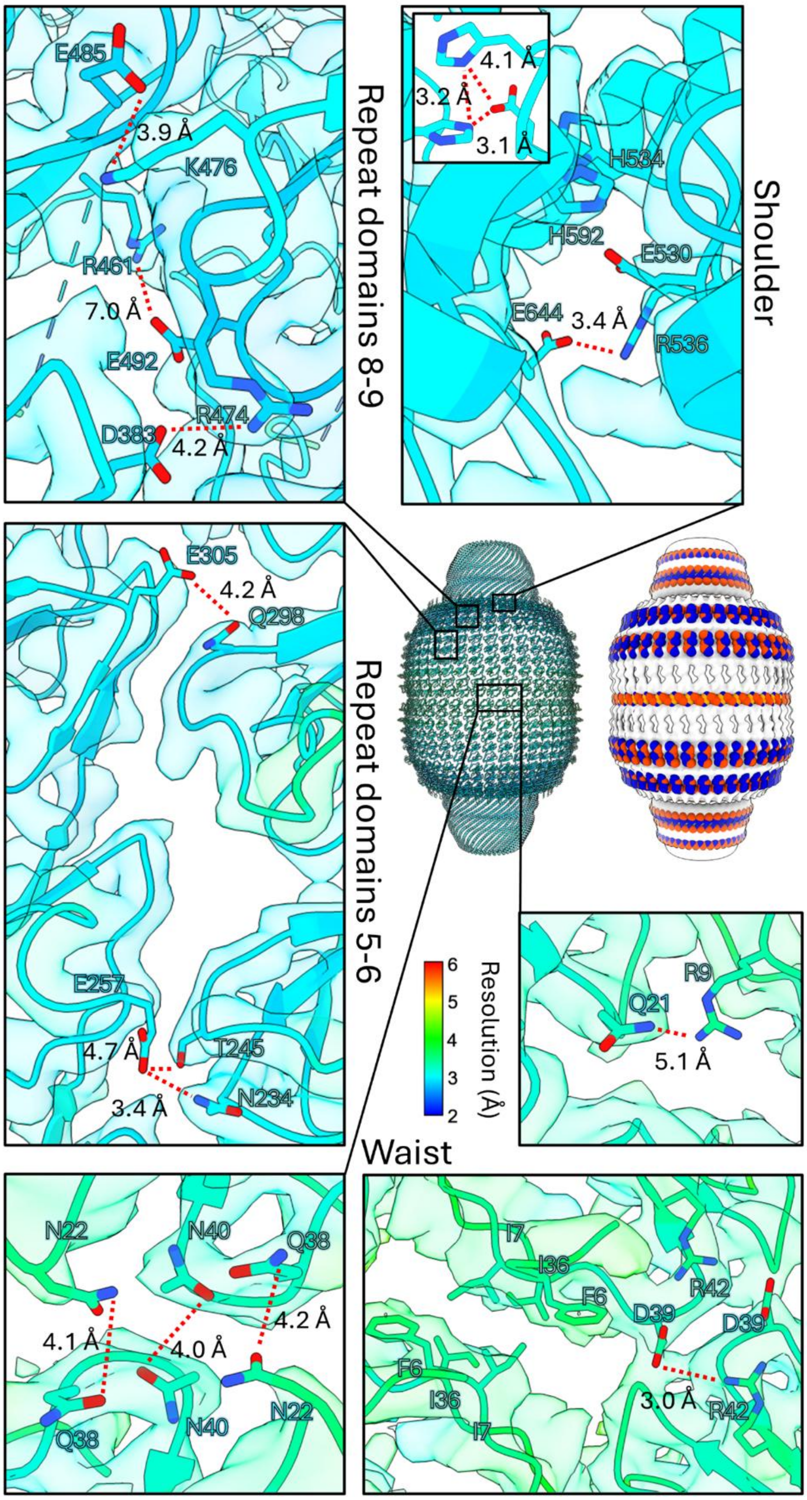

Supplementary Figure 13. **MVP:MVP contacts.** Regions of contacts between MVP monomers are displayed in the figure with whole atomic model next to the cryo-EM density map (downscaled) as reference. The positions of the amino acids involved in the main MVP:MVP contacts are displayed on the density map as spheres colored either in red and blue or orange for polar interactions and hydrophobic, respectively. Inserts display the atomic model of the vault in primed conformation superimposed to the cryo-EM sharpened map (contour level between 0.7 and 1.2) with resolution shown in color (rainbow scale), with labels for the main residues taken into consideration and lines that measure the interatomic distance.

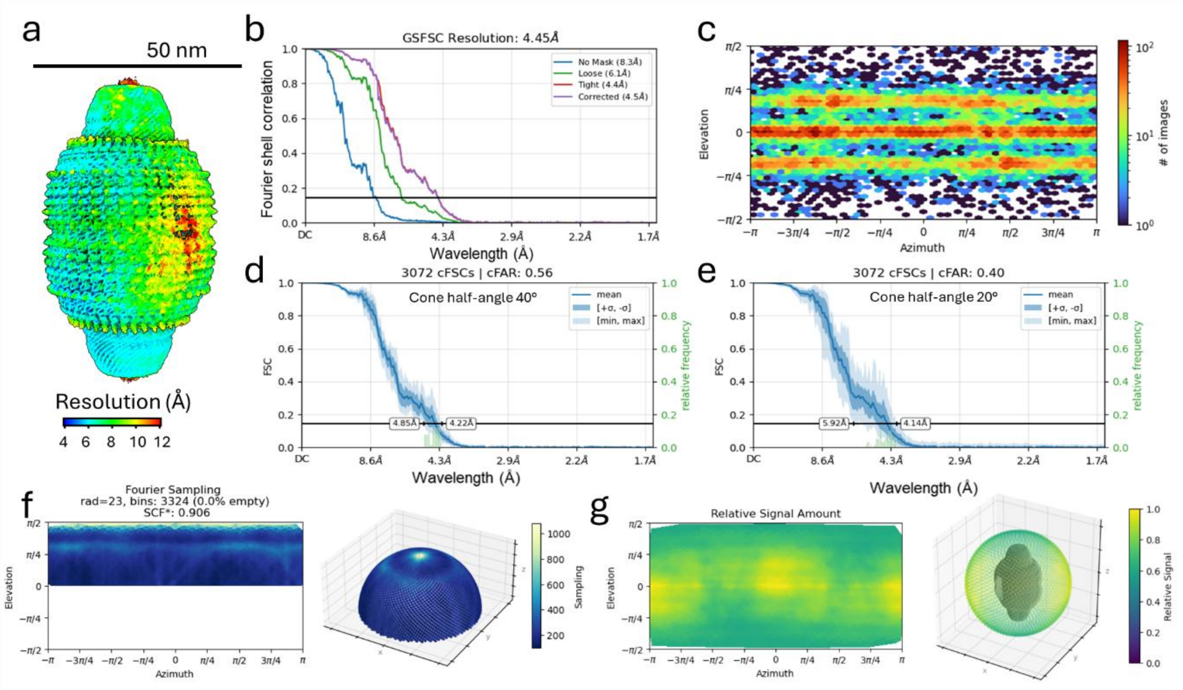

Supplementary Figure 14. **Reconstructed maps of the vault in committed conformation. a** Local resolution of the (unsharpened) map resulting from the cryo-EM reconstruction of the vault in the committed conformation, shown in rainbow color scale. **b** Corresponding gold standard Fourier shell correlation (GS-FSC) curves with crossing at 0.143 and GS-FSC resolution reported on top. **c** 2D orientation plot where cartesian coordinates of the map corresponds to two of the Euler angles and color represents the orientation distribution. **d, e** Conical Fourier shell correlation area ratio (cFAR) plots for 3072 conical masks with half-angle of either 20° (d) or 40° (e) uniformly distributed across the viewing directions (Fibonacci sampling). **f** Sampling Compensation Factor (SCF*) plot measuring the Fourier representation of the alignments of the particle set on cartesian azimuth-elevation chart and on a hemisphere. **g** Relative signal amount plots, showing how FSC curves computed within a toroidal section of the half-maps correlate on an azimuth-elevation chart, and a 3D viewing sphere encircling a low-passed (chiral) map of the vault in committed conformation. Source data are provided as a Source Data file.

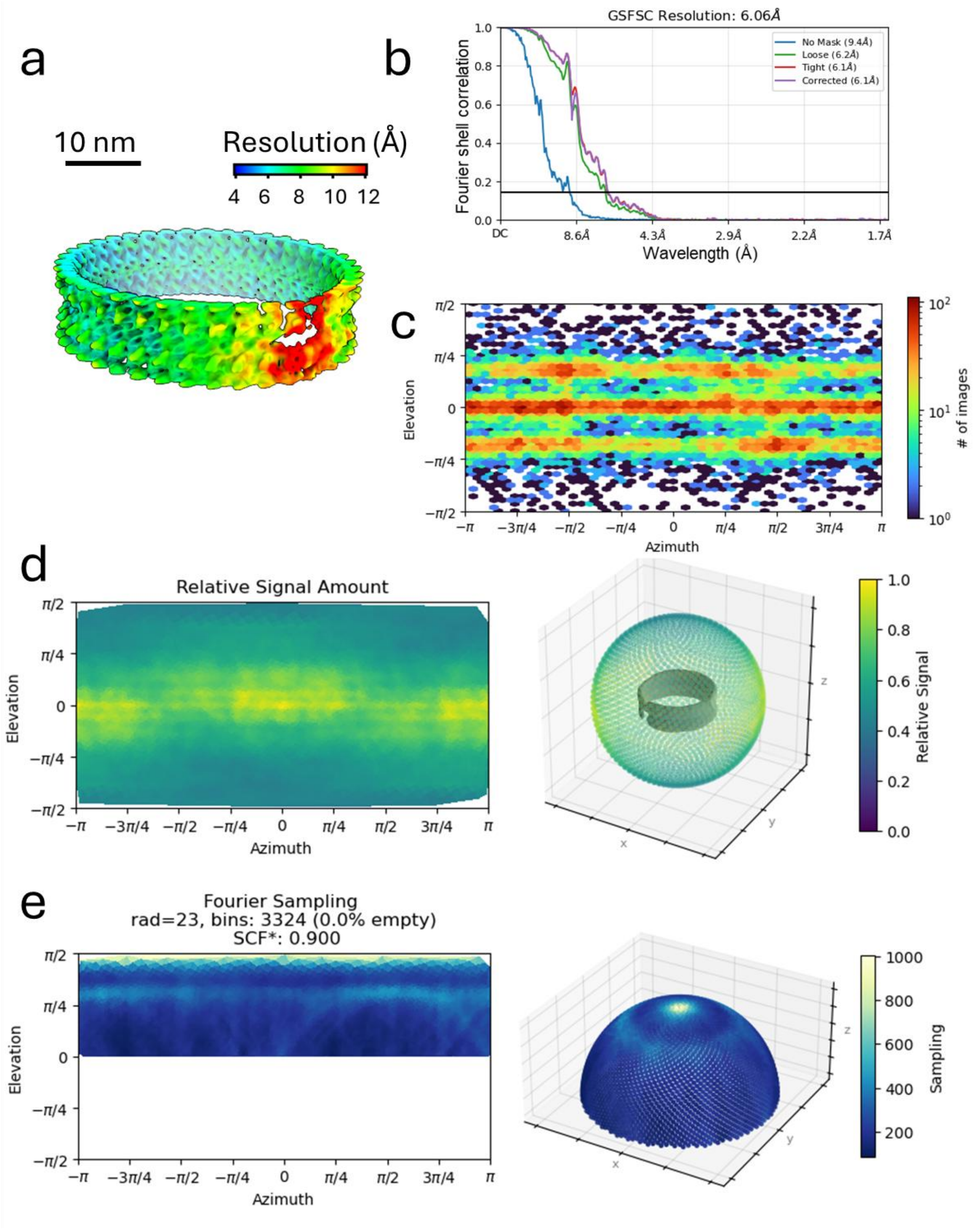

Supplementary Figure 15. **Reconstructed local maps of the vault's waist in committed conformation. a** Local resolution of the (unsharpened) map resulting from the cryo-EM reconstruction of the vault's waist in the committed conformation, shown in rainbow color scale. **b** Corresponding gold standard Fourier shell correlation (GS-FSC) curves with crossing at 0.143 and GS-FSC resolution reported on top. **c** 2D orientation plot where cartesian coordinates of the map corresponds to two of the Euler angles and color represents the orientation distribution. **d** Relative signal amount plots, showing how FSC curves computed within a toroidal section of the half-maps correlate on an azimuth-elevation chart, and a 3D viewing sphere encircling a low-passed (chiral) local map of the vault's waist in committed conformation. **e** Sampling Compensation Factor (SCF*) plot measuring the Fourier representation of the alignments of the particle set on cartesian azimuth-elevation chart and on a hemisphere. Source data are provided as a Source Data file.

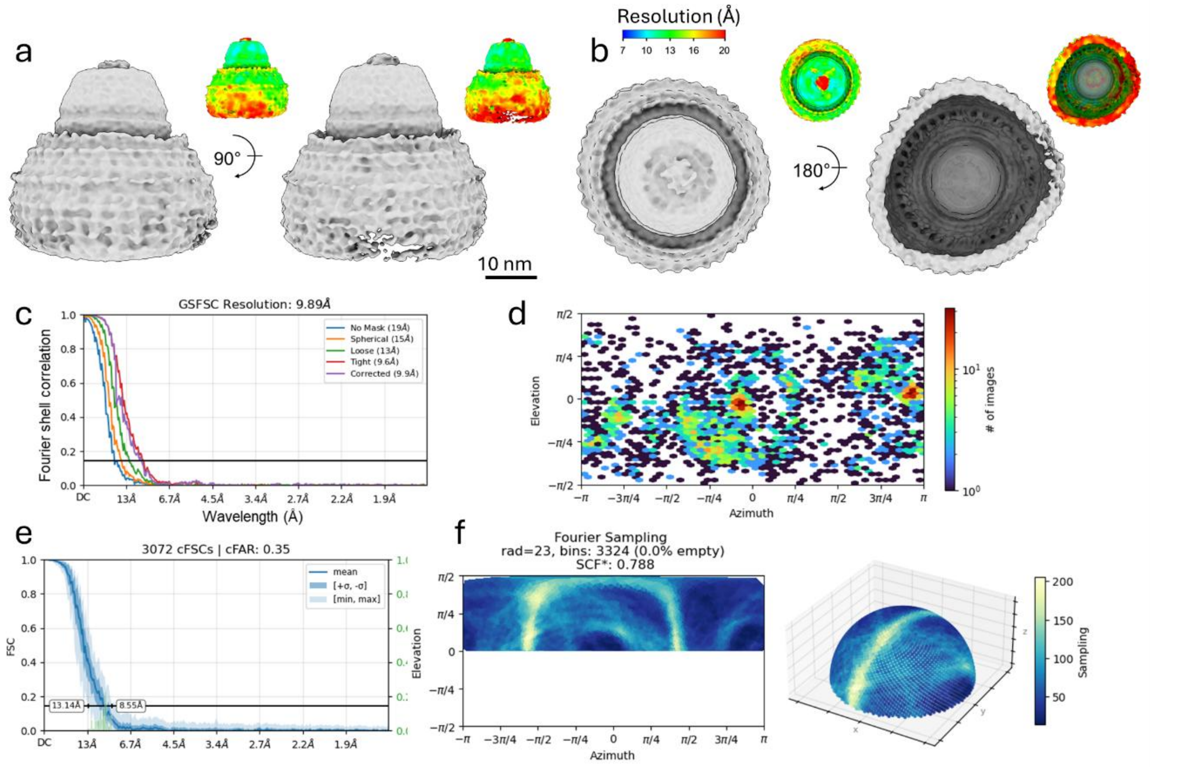

Supplementary Figure 16. **Refinement of the 39-mer half vault**. **a** Lateral views of the non-uniform refinement of the 39-mer half vault, obtained from 3,069 particles (contour level 0.3). Local resolution shown in rainbow color scale. **b** Top and bottom views of the non-uniform refinement of the 39-mer half vault. **c** Corresponding GS-FSC curves with crossing at 0.143, calculated between two independent half-maps using cryoSPARC, with resolution values reported at 0.143 FSC. **d** 2D orientation plot where cartesian coordinates of the map corresponds to two of the Euler angles and color represents the orientation distribution. **e** Conical Fourier shell correlation area ratio (cFAR) plots for 3072 conical masks with half-angle of 20° uniformly distributed across the viewing directions (Fibonacci sampling). **f** Sampling Compensation Factor (SCF*) plot measuring the Fourier representation of the alignments of the particle set on cartesian azimuth-elevation chart and on a hemisphere. Source data are provided as a Source Data file.

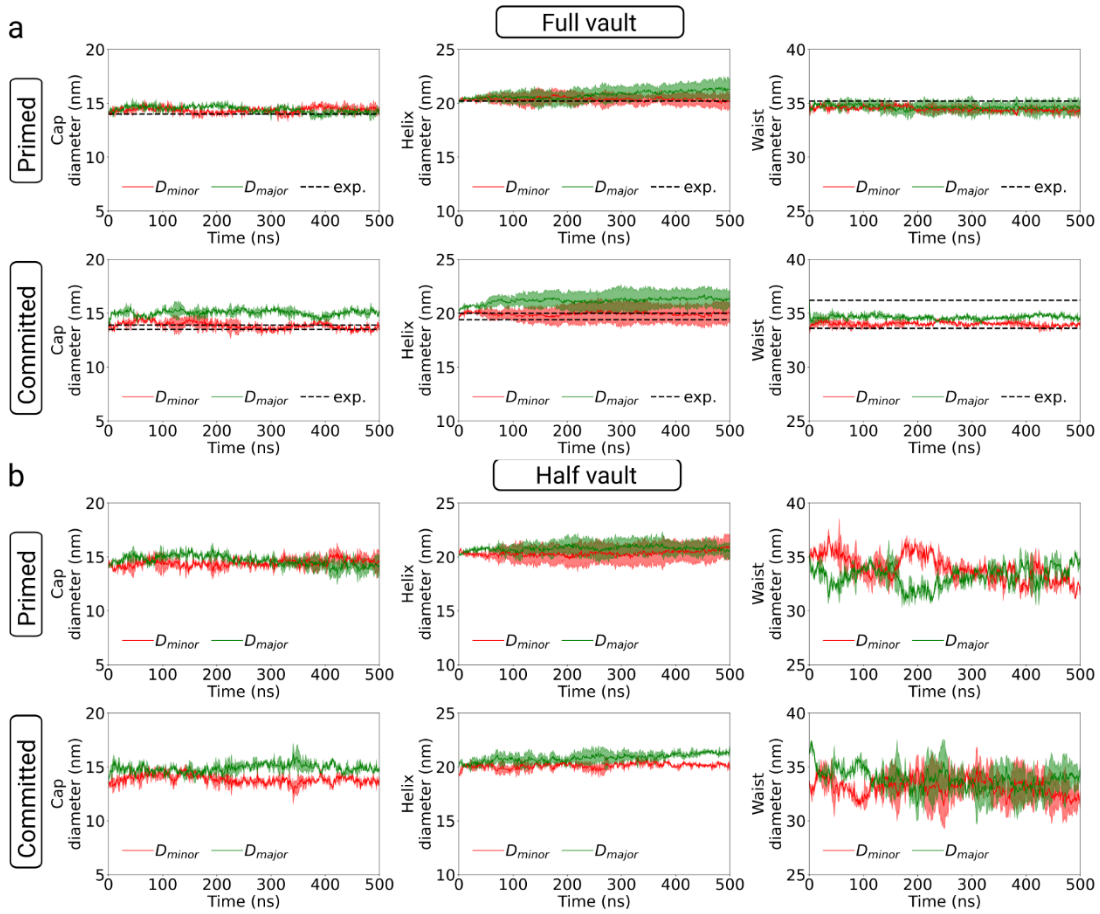

Supplementary Figure 17. **Diameter of cap, helix and waist regions in AA-MD simulations across vault conformations. a** Time series of the vault's cap (left), helix (center), and waist (right) diameters in the primed (top) and committed (bottom) conformations for the full vault. Diameters are obtained by fitting an ellipse to the backbone bead positions of residues A801 (cap), Q678 (helix), and D39 (waist), with major and minor diameters shown in green and red, respectively. Mean values (solid lines) and two standard deviations (shaded regions) from two simulation replicates are plotted. Dashed lines indicate diameters from the atomic model. **b** Time series of the vault's cap (left) and helix (right) diameters in the primed (top) and committed (bottom) conformations for the half vault, estimated in the same way as in panel a. Source data are provided as a Source Data file.

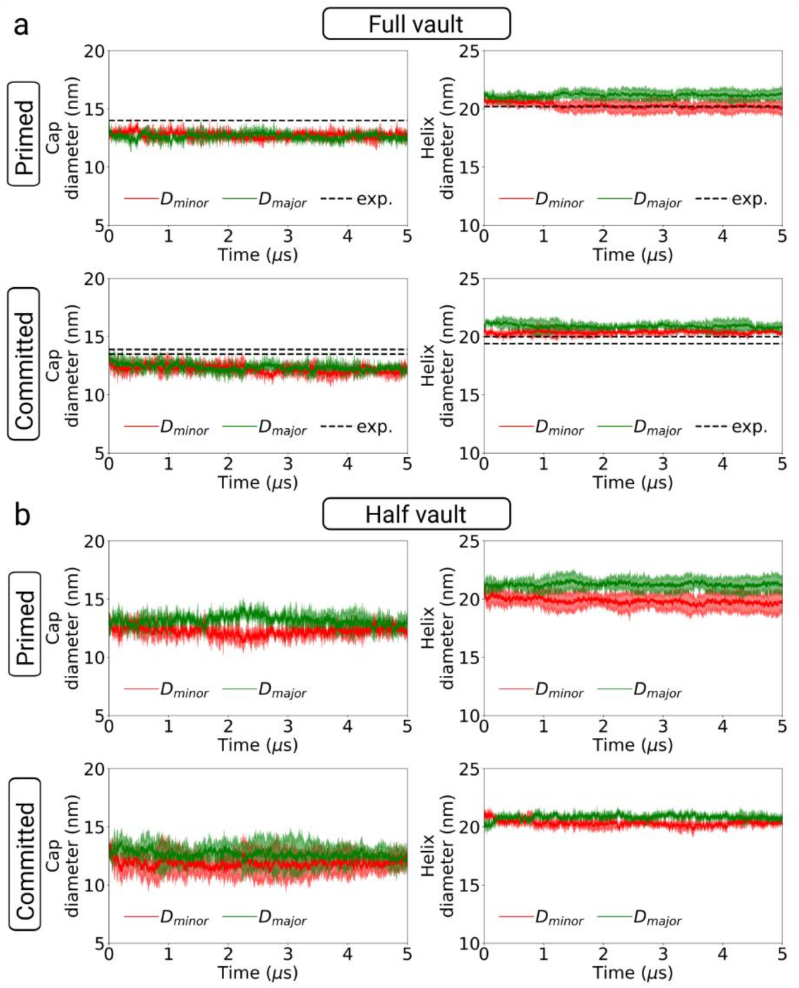

Supplementary Figure 18**. Diameter of cap and helix regions in CG-MD simulations across vault conformations. a** Time series of the vault's cap (left) and helix (right) diameters in the primed (top) and committed (bottom) conformations for the full vault. Diameters are obtained by fitting an ellipse to the backbone bead positions of residues A801 (cap) and Q678 (helix), with major and minor diameters shown in green and red, respectively. Mean values (solid lines) and two standard deviations (shaded regions) from three simulation replicates are plotted. Dashed lines indicate diameters from the atomic model. **b** Time series of the vault's cap (left) and helix (right) diameters in the primed (top) and committed (bottom) conformations for the half vault, estimated in the same way as in panel a. Source data are provided as a Source Data file.

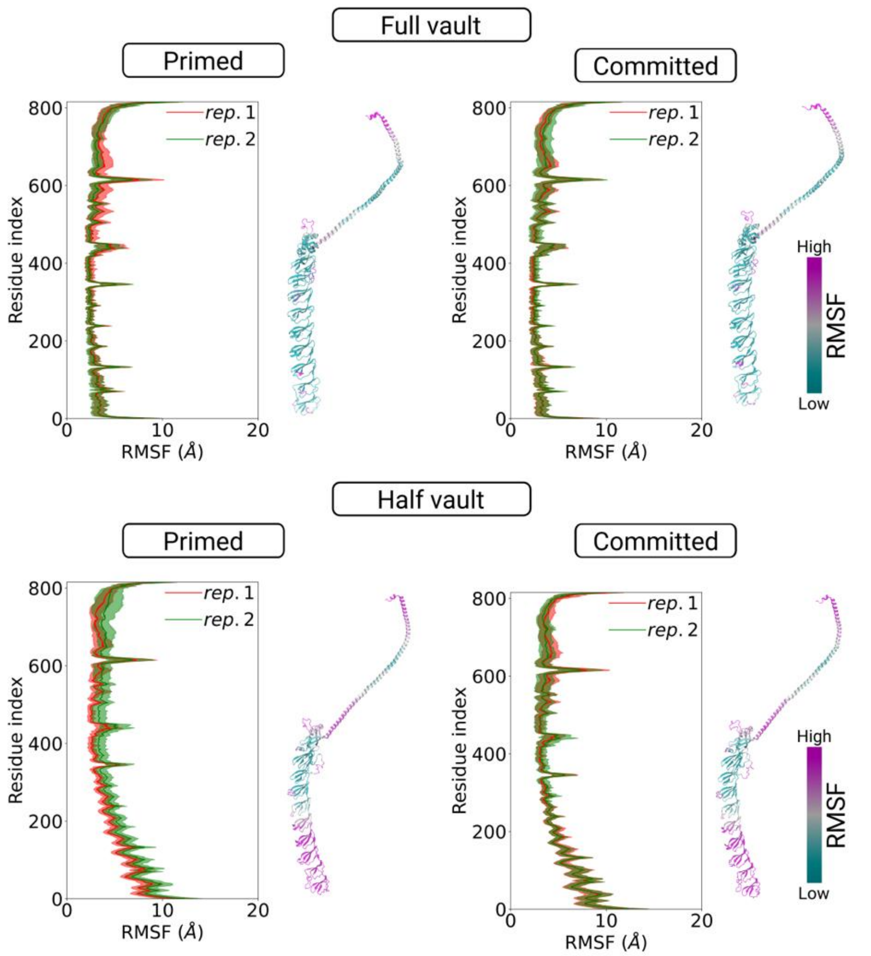

Supplementary Figure 19. **Per residue RMSF from all-atom MD simulations of the full and half-vault particle**. The relative mean square fluctuation (RMSF) per residue as observed during MD simulations of the full vault (top) and half vault (bottom) in primed (left) and committed (right) conformations. For each simulation the RMSF was computed for every MVP chain and then averaged over all chains. The central line corresponds to the average RMSF, and the transparent area shows the standard deviation. The RMSF values were mapped onto a single chain in each conformation using a colour gradient (light blue= rigid, dark pink= flexible) to highlight regions of greatest motion. Source data are provided as a Source Data file.

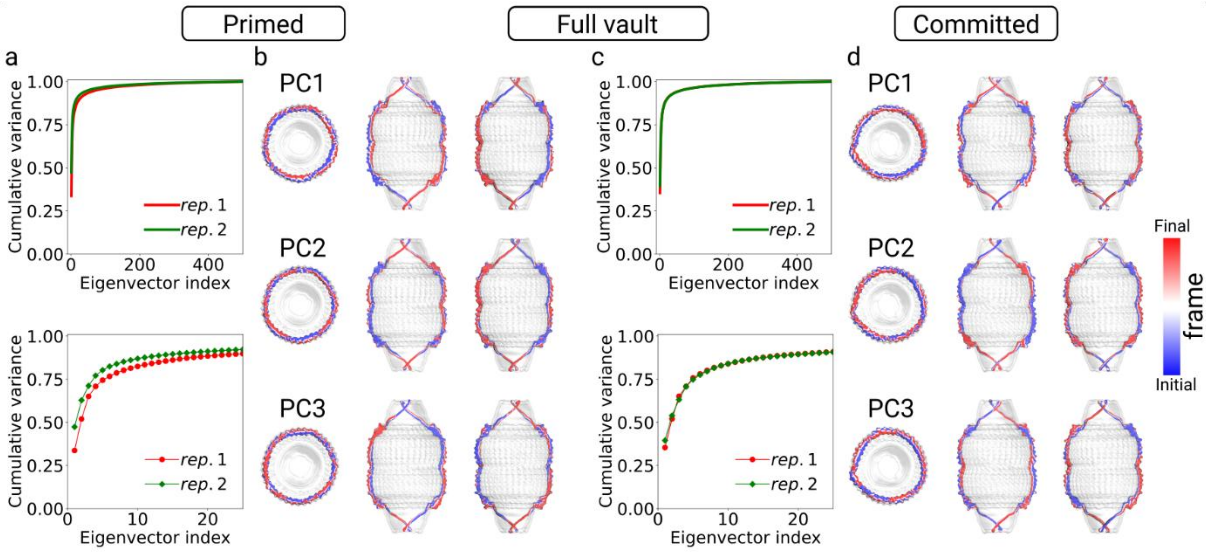

Supplementary Figure 20. **Principal-component analysis (PCA) of all-atom MD trajectories for the full vault particle. a, c** Cumulative variance. PCA was performed on the centre-of-mass (CoM) coordinates of every fifth residue along each MVP chain over the entire simulation. Top: Fraction of total variance captured as a function of the number of PCs (ordered by decreasing eigenvalue). Bottom: Same plot restricted to the first 25 PCs for the primed (a) and committed (c) conformations. **b, d** Structural projections onto the three leading PCs. For each conformation, the first (PC1), second (PC2) and third (PC3) eigenvectors are visualised as colour-mapped deformations of single MVP chains; the average structure is shown as a transparent surface. Three orthogonal views are displayed for each PC (left, centre, right). Left: view from the bottom of a half-vault. Centre: view of two chains located on opposite sides of the particle. Right: view of two chains positioned 90° around the vault relative to the centre view. The colour scale runs from blue (minimum projection) to red (maximum projection), highlighting the regions that contribute most to each principal motion. Source data are provided as a Source Data file.

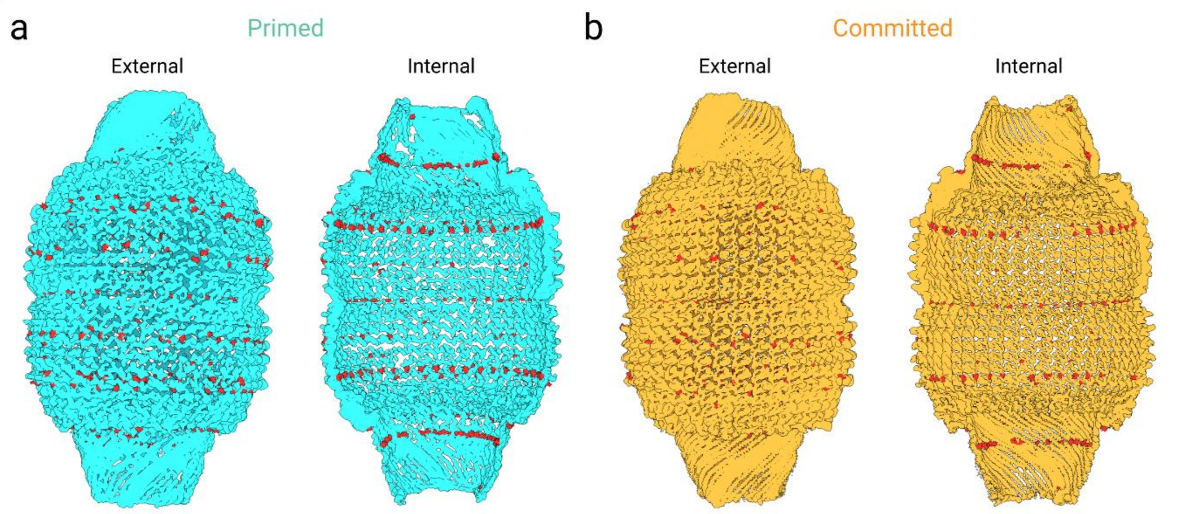

Supplementary Figure 21. **Sodium-ion density around the full vault in the primed and committed conformation from all-atom MD simulations.** Na^+ density was computed by averaging ion positions over the entire trajectory and is shown as a red surface. The time-averaged vault particle structure of the **a** primed and **b** committed vault. Left: External (outside-in) perspective of the vault, illustrating the overall distribution of Na^+ around the particle surface. Right: Internal perspective (cut-away) that reveals ion accumulation inside the vault cavity.

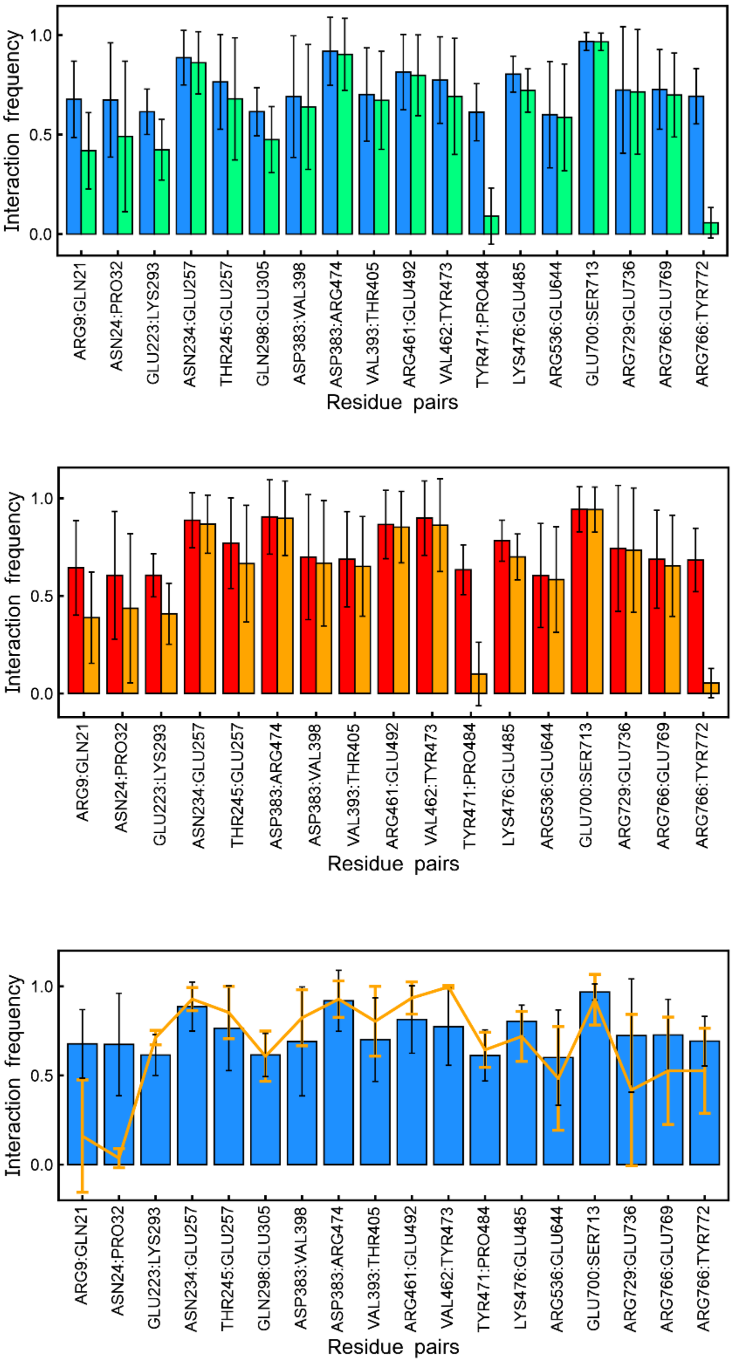

Supplementary Figure 22. **Analysis of lateral interactions between MVP chains in the full vault as observed from the MD simulations.** The top panel depicts the most frequent interactions observed in the full vault in the primed conformation. Blue bars depict the frequency of van der Waals interactions, while the green bars show the persistence of hydrogen bonds. The middle panel depicts the most frequent interactions observed in the full vault in the committed conformation. Red bars depict the frequency of van der Waals interactions, while the orange bars show the persistence of hydrogen bonds. The bottom panel depicts how van der Waals interactions are affected by the rupturing of the full vault. Blue bars report van der Waals interaction persistence in the primed full vault (blue bars), while the orange line depicts the frequency of these interactions between chains lining the opening. The largest decrease in the interaction frequency was observed at the N-terminus, located at the waist region. Data are presented as mean values ± SD ($n = 3{,}978$ for lateral interaction frequencies and $n = 408$ for lateral interaction frequencies surrounding the defect area). Source data are provided as a Source Data file.

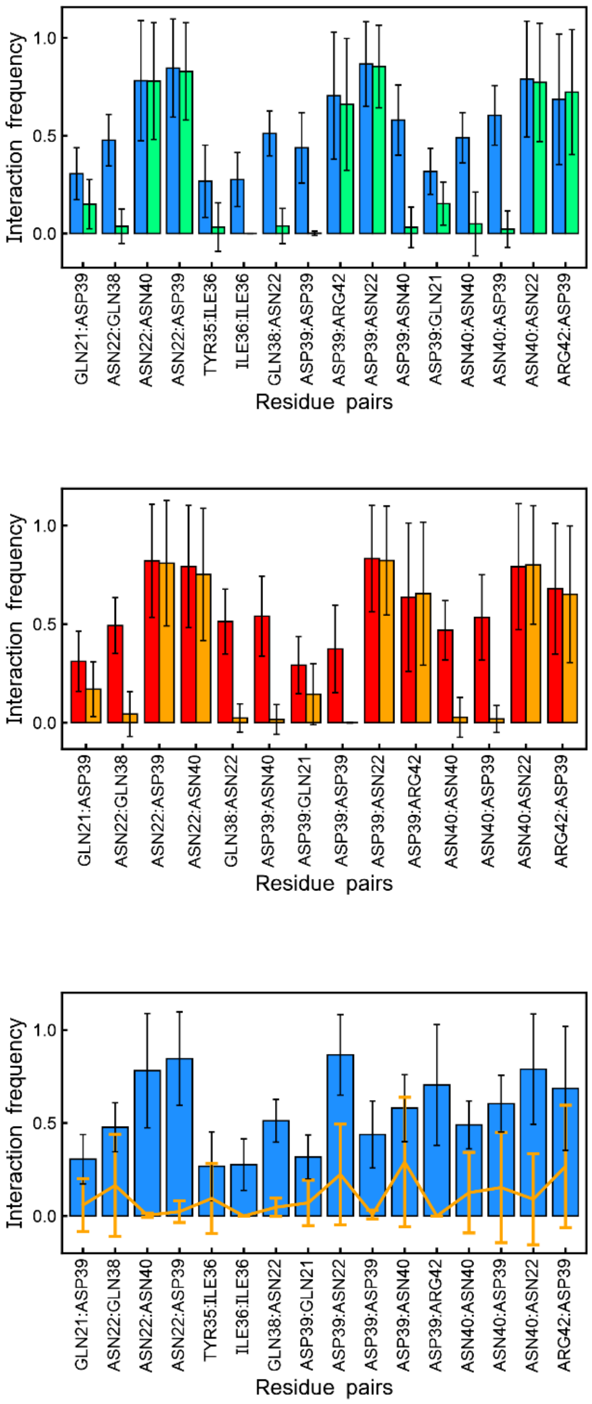

Supplementary Figure 23. **Analysis of frontal interactions between MVP chains present at the waist of the full vault as observed from the MD simulations.** The top panel depicts the most frequent interactions observed in the full vault in the primed conformation. Blue bars depict the frequency of van der Waals interactions, while the green bars show the persistence of hydrogen bonds. The middle panel depicts the most frequent interactions observed in the full vault in the committed conformation. Red bars depict the frequency of van der Waals interactions, while the orange bars show the persistence of hydrogen bonds. The bottom panel depicts how van der Waals interactions at the waist are affected by the rupturing of the full vault. Blue bars report van der Waals interaction persistence in the primed full vault (blue bars), while the orange line depicts the frequency of these interactions between chains lining the opening. Data are presented as mean values ± SD ($n = 7{,}956$ for horizontal interaction frequencies and $n = 306$ for horizonal interaction frequencies surrounding the defect area). Source data are provided as a Source Data file.

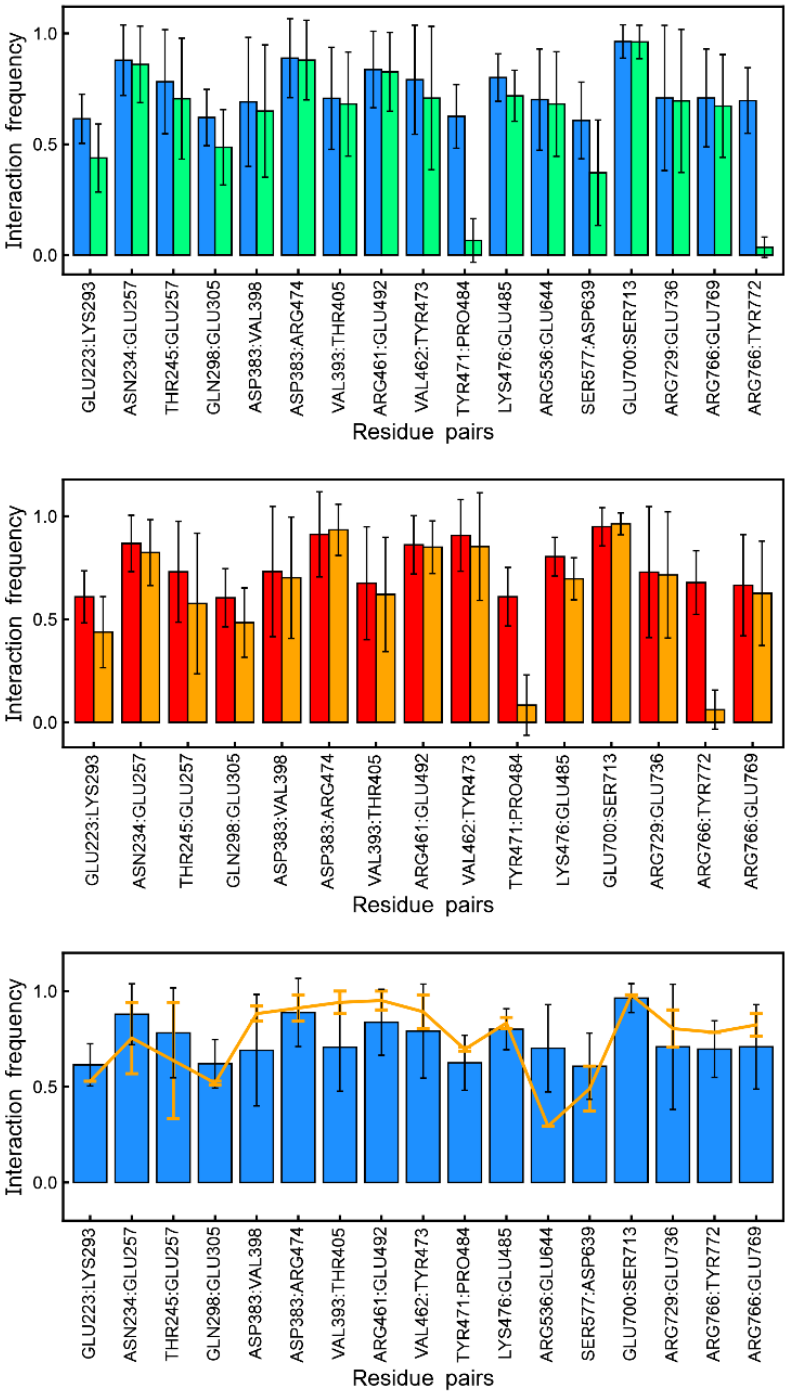

Supplementary Figure 24. **Analysis of lateral interactions between MVP chains in the half vault as observed from the MD simulations.** The top panel depicts the most frequent interactions observed in the half vault in the primed conformation. Blue bars depict the frequency of van der Waals interactions, while the green bars show the persistence of hydrogen bonds. The middle panel depicts the most frequent interactions observed in the half vault in the committed conformation. Red bars depict the frequency of van der Waals interactions, while the orange bars show the persistence of hydrogen bonds. The bottom panel depicts how van der Waals interactions are affected by the rupturing of the half vault. Blue bars report van der Waals interaction persistence in the primed half vault (blue bars), while the orange line depicts the frequency of these interactions between chains lining the opening. Data are presented as mean values ± SD ($n = 3{,}978$ for lateral interaction frequencies and $n = 408$ for lateral interaction frequencies surrounding the defect area). Source data are provided as a Source Data file.

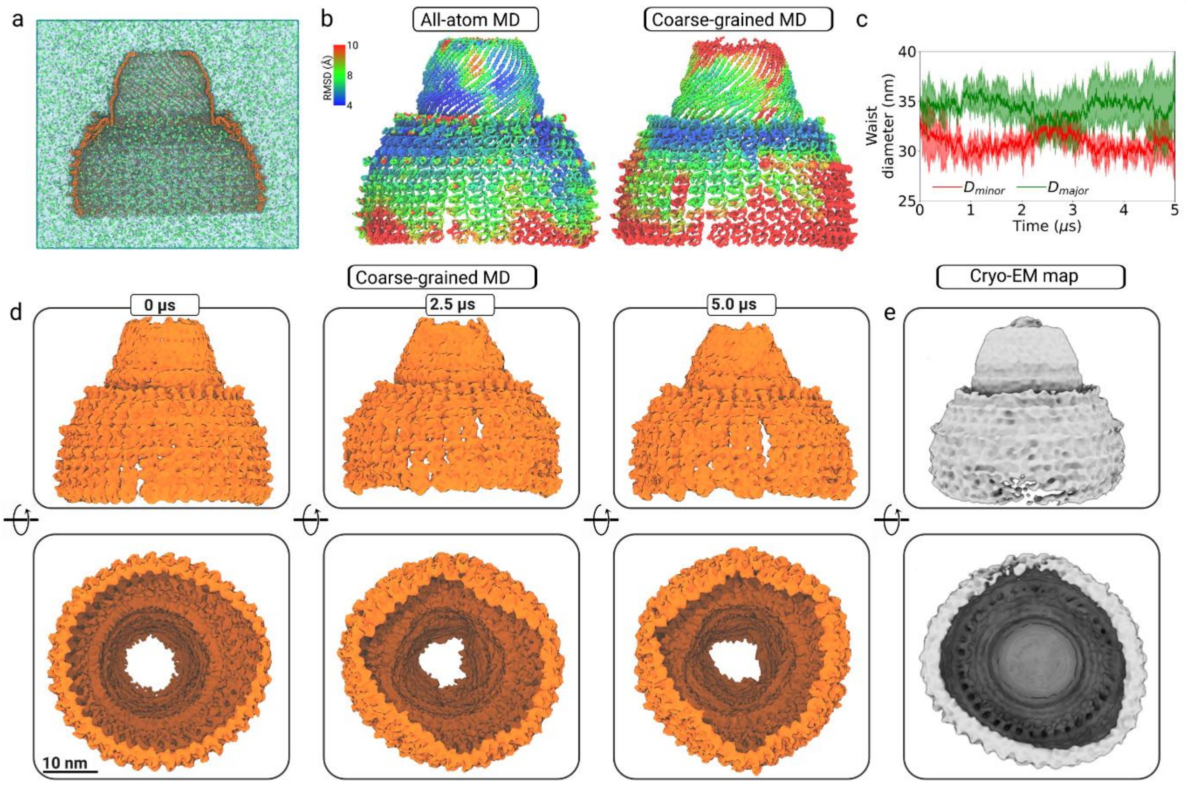

Supplementary Figure 25. **All-atom (a–b) and coarse-grained (c–d) molecular dynamics simulations of the half-vault in the committed conformation**. **a** Cutaway view of the all-atom MD simulation box containing half of the human vault particle in the committed conformation (orange), solvated in water (cyan surface) with Na^+ (purple) and Cl^- (green) ions. **b** Left panel, RMSD over the last 200 ns of simulation, mapped onto the initial atomic structure of the half-vault in the committed conformation. Structures are aligned to the Cα atoms of residues with RMSF <3.5 Å. Right panel, RMSD over the last 4.5 μs of simulation, mapped onto the initial coarse-grained structure of the half-vault in the committed conformation. Structures are aligned to the backbone beads of residues with RMSF <3.5 Å. **c** Time series of the half vault's waist diameter in the committed conformation. Diameters are obtained by fitting an ellipse to the backbone bead positions of residues D39, with major and minor diameters shown in green and red, respectively. Mean values (solid lines) and two standard deviations (shaded regions) from three simulation replicates are plotted. **d** Coarse-grained MD simulations of the half-vault in the committed conformation. Representative snapshots from the MD trajectory are shown from a lateral view (top) and an internal bottom-up view (bottom). The coarse-grained structures are represented as surfaces (contour level 1.3). **e** Lateral (top) and internal bottom-up (bottom) views of the non-uniform cryo-EM refinement of the 39-mer half-vault, reconstructed from 3,069 particles. Source data are provided as a Source Data file.

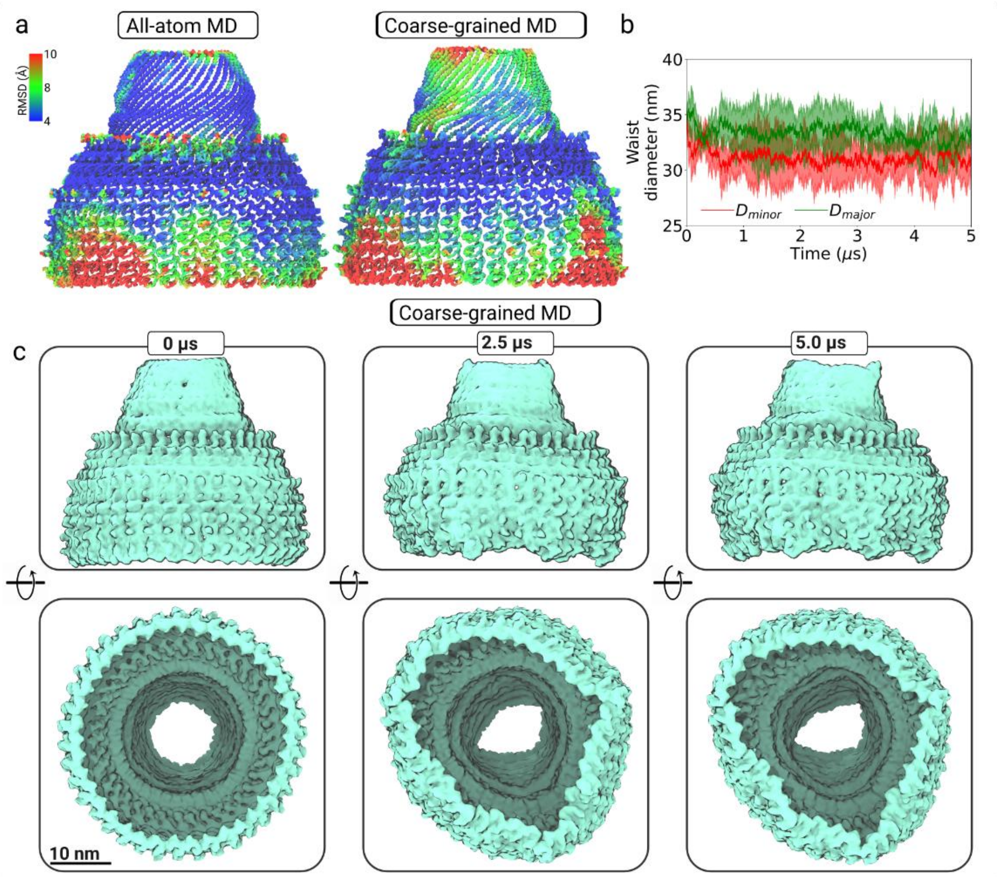

Supplementary Figure 26. **All-atom (a) and coarse-grained (b–c) simulations of the half-vault in the primed conformation. a** Left panel, RMSD over the last 200 ns of simulation, mapped onto the initial atomic structure of the half-vault in the primed conformation. Structures are aligned to the Cα atoms of residues with RMSF <3.5 Å. Right panel, RMSD over the last 4.5 μs of simulation, mapped onto the initial coarse-grained structure of the half-vault in the primed conformation. Structures are aligned to the backbone beads of residues with RMSF <3.5 Å. **b** Time series of the half vault's waist diameter in the primed conformation. Diameters are obtained by fitting an ellipse to the backbone bead positions of residues D39, with major and minor diameters shown in green and red, respectively. Mean values (solid lines) and two standard deviations (shaded regions) from three simulation replicates are plotted. **c** Coarse-grained MD simulations of the half-vault in the primed conformation. Representative snapshots from the MD trajectory are shown from a lateral view (top) and an internal bottom-up view (bottom). The coarse-grained structures are represented as surfaces (contour level 1.3). Source data are provided as a Source Data file.

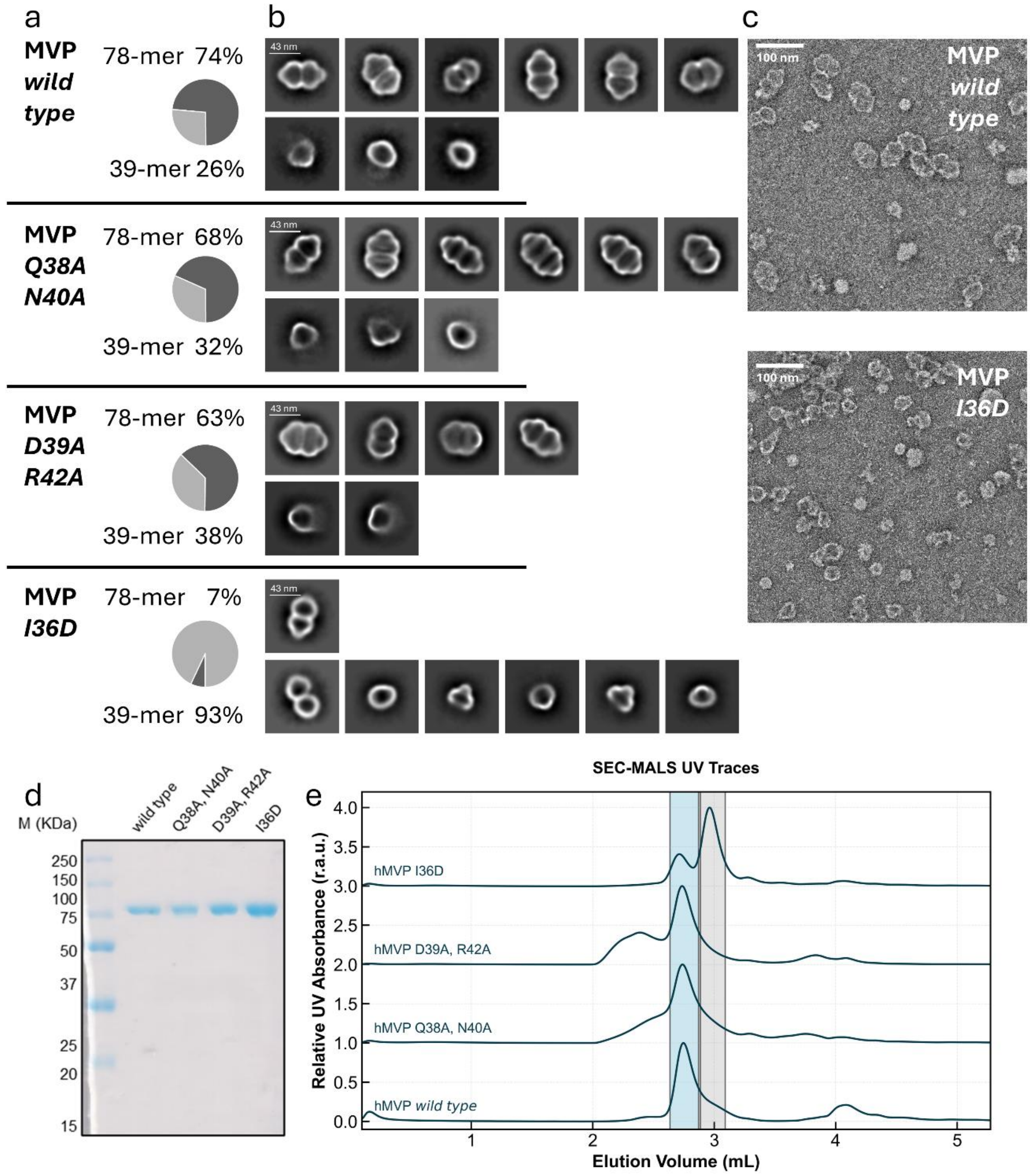

Supplementary Figure 27. **MVP variants. a** Name of the MVP variants and ratio of fully assembled vaults (78-mer) and halves (39-mer) quantified with 2D classification on negative-stain TEM micrographs. **b** Representative 2D class averages for each MVP sample used for the quantification. Scale bar = 43 nm. **c** Representative micrographs (n=750 micrographs) of two samples (MVP wild type and MVP I36D). Scale bar = 100 nm. **d** SDS-PAGE of the samples after purification, markers (M) followed by protein samples. **e** SEC-MALS relative UV traces for each sample. The two sections highlighted in cyan and grey in the chromatographs correspond to the elution volume of the 78-mer and 39-mer, respectively. Source data are provided as a Source Data file.

Supplementary Table 1. **SAXS data collection and analysis.** Model free parameters extracted from collected SAXS curves. Expected Mw from SEC-MALS measurements is 7.66 MDa, while the expected Rg, calculated from the cryo-EM structure of the MVP in the primed conformation is 23 nm. Mw and Rg values obtained by SAXS analysis are in reasonable agreement with expected values, indicating MVP is well folded in solution.

**MVP sample**	**Merged***	**2.6 mg/ml**	**1.3 mg/ml**	**0.65 mg/ml**
**Organism**	*K. phaffii*			
**Source**	Homo sapiens			
**uniprot ID**	MVP_HUMAN (Q14764)			
**Extinction coefficient ε**	5003115 M^{-1} cm^{-1} (280 nm)			
**Calculated Molecular mass**	7,747,506 Da			
**Solvent composition**	25 mM HEPES, 150 mM NaCl, and 1 mM TCEP (pH 7.5)			
**Sample temperature (ºC)**	20 °C			
**Source**	BM29, BioSAXS, ESRF	P12, PETRA-III, DESY		
**Wavelength (Å)**	0.992	1.2397		
**Sample-detector distance (m)**	2.869	3.0		
**Detector**	PILATUS3 2M	Pilatus 6 M		
***q*-measurement range (nm^{-1})**	0.025–6	0.020–4.5		
**Exposure time × n. of exposures**	1 sec × 10	0.1 sec × 20		
**Concentration measured**	0.5 mg/ml and 0.3 mg/ml	2.6 mg/ml	1.3 mg/ml	0.65 mg/ml
**I(0) from Guinier#**	6.0 ± 0.9	6.5 ± 0.5	6.5 ± 0.3	6.5 ± 0.6
**Rg from Guinier (nm)#**	23 ± 5	31 ± 4	30 ± 4	29 ± 5
**Dmax (nm)**	65	73	76	75
**Mw from Porod volume (MDa)**	8.6	9.3	8	8.5
**Porod exponent**	2.9	3.7	3.9	4
**SASDB identifier**	SASDXJ3	/	/	/

*The sample listed as merged was obtained by merging two measurements carried out at concentration of 0.5 and 0.3 mg/ml

Due to the size of the MVP particle, the results of Guinier analysis, i.e. molecular weight (Mw) from scattering at zero angle (I(0)) and radius of gyration (Rg) are associated with a relatively high error, since only a few points (approximately 4-9) are present in the range of scattering angles (q) where the Guinier approximation holds (qRg < 1.3).

Supplementary Table 2 – Cryo-EM data collection, refinement and validation statistics

	Vault primed (EMD-53415) (PDB 9QW9)	Local refin. waist primed vault (EMD-53438)	Vault committed (EMD-53423) (PDB 9QWQ)	Local refin. waist committed vault (EMD-53439)	39-mer half vault (EMD-53440)
**Data collect. and processing**					
Magnification	81,000	81,000	81,000	81,000	81,000
Voltage (kV)	300	300	300	300	300
Electr. expos. (e–/Å^2)	50.15	50.15	50.15	50.15	50.15
Defocus range (μm)	0.55-2.33	0.55-2.33	0.55-2.33	0.55-2.33	0.55-2.33
Pixel size (Å)	0.8416	0.8416	0.8416	0.8416	0.8416
Symmetry imposed	D39	D39	Relaxed D39	C1	C1
Initial particles	63,814	63,814	63,814	63,814	63,814
Final particles	11,172	11,172	23,998	23,998	3,069
Map resolution (Å)	3.09	3.53	4.45	6.06	9.89
FSC threshold	0.143	0.143	0.143	0.143	0.143
Map resolution. range (Å)	26.98 – 2.23	5.29 – 2.64	46.55 – 3.84	12.20 – 4.65	24.7 – 8.69
**Refinement**					
Initial model used (PDB code)	4HL8		4HL8		
Model resolution (Å)	3.1		6.4		
FSC threshold	0.5		0.5		
Model resolution range (Å)	5.67 – 2.68		46.02 – 3.84		
Map sharpening *B* factor (Å^2)	-144.4		-110.9		
Model composition					
Non-hydrogen atoms	482,118		482,118		
Protein residues	60,762		60,762		
B factors (Å^2)					
Protein	47.7/200.6/105.0		169.7/1051.29/386.74		
R.m.s. deviations					
Bond lengths (Å)	0.004 (0)		0.004 (1)		
Bond angles (°)	0.941 (0)		0.958 (11)		
Validation					
MolProbity score	1.92		2.43		
Clashscore	5.31		18.29		
Poor rotamers (%)	1.78		0.00		
Ramachandran plot					
Favored (%)	93.07		84.50		
Allowed (%)	6.85		15.42		
Disallowed (%)	0.08		0.08		

Supplementary Table 3 – Description of the different MD production simulations reported in this work

System	Conformation	Type of MD	# replicates	Duration [ns]	Box size [nm]	# particles
Vault particle	Primed	AA-MD	2	500	78.1x45.5x52.5	18358261
Vault particle	Committed	AA-MD	2	500	78.3x46.0x51.7	18363369
Vault particle	Primed	CG-MD	3	5000	77.9x45.3x52.3	1555766
Vault particle	Committed	CG-MD	3	5000	78.0x45.8x51.5	1560076
Half vault particle	Primed	AA-MD	2	500	52.5x45.5x45.2	10611232
Half vault particle	Committed	AA-MD	2	500	52.8x44.7x46.0	10710501
Half vault particle	Primed	CG-MD	3	5000	52.3x45.3x45.0	893571
Half vault particle	Committed	CG-MD	3	5000	52.5x44.5x45.7	896709

Supplementary Discussion

The map of the committed vault at 4.45 Å GS-FSC resolution shows a prominent loss in local resolution in correspondence with the symmetry-mismatched component at the waist (**Supplementary Figs. 14a** and **14b**). The orientation distribution (**Supplementary Fig. 14c**) and the Sampling Compensation Factor (SCF*) of 0.906 (**Supplementary Fig. 14f**) suggested that Fourier space is well-sampled[59,60]. In contrast, the conical FSC Area Ratio (cFAR),which probes the directional anisotropy[61,62], revealed direction-dependent loss of signal (**Supplementary Fig. 14d**). Taken together, this indicated that the anisotropy could have arisen from the heterogeneity of the sample or from poorly aligned particles. Furthermore, when expanding the cone half-angle from the standard 20° to a broader range of directions of 40°, we observed an improvement in cFAR (from 0.40 to 0.56) (**Supplementary Fig. 14e**). This resulted from averaging out localized drops in signal intensity and suggested that the loss of signal is localized and not systematic, also in line with the relative signal amount plot, which shows a decrease in FSC for the orientations in correspondence with the symmetry breaking element of the map (**Supplementary Fig. 14g**). Overall, this is consistent with the presence of a region at lower local resolution, as observed in the map, and it is in line with the localized flexibility found with 3DVA, and PCA of the particles, which showed heterogeneity localized in the low-resolution symmetry-breaking region (**Supplementary Movie 1** and **Supplementary Figs. 5** and **6**).
