# Supplementary material for: Structural flexibility of the human vault particle revealed by high-resolution cryo-EM and molecular dynamics simulations
Source: Nat Commun. 2026 May 2;17:6033. doi: 10.1038/s41467-026-72674-4 (PMC13347060; doi:10.1038/s41467-026-72674-4)
Supplement: Supplementary file 2 — Description of Additional Supplementary Files [file 41467_2026_72674_MOESM2_ESM.pdf]

Description of Additional Supplementary Files

File name: Supplementary Movie 1
Description: 3D Variability Analysis of the initial stack of 35,429 vault particles. The different trajectories (modes) in 3D volumes show where there is significant variability in the dataset.

File name: Supplementary Movie 2
Description: Representative trajectory of the CG-MD simulations. Vault in primed (left) and committed (right) conformations. The coarse-grained structures are shown as surfaces (contour level 1.3).

File name: Supplementary Movie 3
Description: Principal-component analysis (PCA) of all-atom and coarse-grained MD trajectories for the full vault particle Vault in primed (left) and committed (right) conformations. Structural projections onto the two leading PCs. PCA was performed on the centre-of-mass (CoM) coordinates of every fifth residue along each MVP chain over the entire simulation. The structures are shown as surfaces (contour level 3.2).
