# Supplementary material for: Structural flexibility of the human vault particle revealed by high-resolution cryo-EM and molecular dynamics simulations
Source: Nat Commun. 2026 May 2;17:6033. doi: 10.1038/s41467-026-72674-4 (PMC13347060; doi:10.1038/s41467-026-72674-4)
Supplement: Supplementary file 6 — Reporting Summary [file 41467_2026_72674_MOESM6_ESM.pdf]

nature portfolio

Corresponding author(s): Fabio Lapenta

Last updated by author(s): 24/3/2026

Reporting Summary

Nature Portfolio wishes to improve the reproducibility of the work that we publish. This form provides structure for consistency and transparency in reporting. For further information on Nature Portfolio policies, see our Editorial Policies and the Editorial Policy Checklist.

Statistics

For all statistical analyses, confirm that the following items are present in the figure legend, table legend, main text, or Methods section.

n/a	Confirmed	
☐	☒	The exact sample size (*n*) for each experimental group/condition, given as a discrete number and unit of measurement
☐	☒	A statement on whether measurements were taken from distinct samples or whether the same sample was measured repeatedly
☒	☐	The statistical test(s) used AND whether they are one- or two-sided<br>*Only common tests should be described solely by name; describe more complex techniques in the Methods section.*
☒	☐	A description of all covariates tested
☒	☐	A description of any assumptions or corrections, such as tests of normality and adjustment for multiple comparisons
☐	☒	A full description of the statistical parameters including central tendency (e.g. means) or other basic estimates (e.g. regression coefficient) AND variation (e.g. standard deviation) or associated estimates of uncertainty (e.g. confidence intervals)
☒	☐	For null hypothesis testing, the test statistic (e.g. *F*, *t*, *r*) with confidence intervals, effect sizes, degrees of freedom and *P* value noted<br>*Give P values as exact values whenever suitable.*
☒	☐	For Bayesian analysis, information on the choice of priors and Markov chain Monte Carlo settings
☒	☐	For hierarchical and complex designs, identification of the appropriate level for tests and full reporting of outcomes
☒	☐	Estimates of effect sizes (e.g. Cohen's *d*, Pearson's *r*), indicating how they were calculated

Our web collection on statistics for biologists contains articles on many of the points above.

Software and code

Policy information about availability of computer code

Data collection	Unicorn v7.0 (Cytiva) FPLC - UV data recording<br>RELION v3.1 for cryo-EM data acquisition<br>GROMACS v2024 for molecular dynamics simulations
Data analysis	ASTRA v7 (Wyatt) for MALS analysis<br>ATSAS v 3.0.4 and PepsiSAXS v3.0 for SAXS analysis<br>Crystal lattice subtraction in Fourier space, program available at: https://github.com/NilsMarechal/SAGsub.<br>cryoSPARC v4.5.3 for cryo-EM data analysis<br>MODELLER v10.5 for homology modelling<br>Coot v0.8.9 for model building<br>Phenix (v1.21) for real-space refinement and symmetry expansion of the model<br>ChimeraX (v1.8) with ISOLDE plugin installed for MDFF<br>VMD (v1.9.4) for MD trajectory visualization<br>MDAnalysis v2.1.0 for MD trajectory analysis<br>Python3.7 for MD trajectory analysis<br>prolif 2.0.3 for analysis of the fequency of residue-residue interactions<br>cryoDRGN (version 3.4.1)

For manuscripts utilizing custom algorithms or software that are central to the research but not yet described in published literature, software must be made available to editors and reviewers. We strongly encourage code deposition in a community repository (e.g. GitHub). See the Nature Portfolio guidelines for submitting code & software for further information.

Data

Policy information about availability of data

All manuscripts must include a data availability statement. This statement should provide the following information, where applicable:

- Accession codes, unique identifiers, or web links for publicly available datasets
- A description of any restrictions on data availability
- For clinical datasets or third party data, please ensure that the statement adheres to our policy

Cryo-EM maps are deposited in the Electron Microscopy Data Bank (EMDB) with the following accession codes EMD-53415 [https://www.ebi.ac.uk/emdb/EMD-53415], EMD-53423 [https://www.ebi.ac.uk/emdb/EMD-53423] and EMD-53440 [https://www.ebi.ac.uk/emdb/EMD-53440], for the vault in primed conformation, committed conformation and for the 39-mer half vault, respectively. Local refinement cryo-EM maps of the vault's waist are deposited on EMDB with the following accession codes EMD-53438 [https://www.ebi.ac.uk/emdb/EMD-53438] and EMD-53439 [https://www.ebi.ac.uk/emdb/EMD-53439] for the vault in primed and committed conformation, respectively.
Atomic structures are deposited in the Protein Data Bank (PDB) with the following accession codes 9QW9 [https://www.rcsb.org/structure/9QW9] and 9QWQ [https://www.rcsb.org/structure/9QWQ] for the human vault in primed and in committed conformation, respectively.
SAXS results are deposited in the Small Angle Scattering Biological Data Bank (SASDB) with accession code SASDXJ3 [https://www.sasbdb.org/data/SASDXJ3].
All-atom and coarse-grained MD trajectories for each replicate and initial configuration have been deposited in the Zenodo entry 19145360 [1https://zenodo.org/records/19145360].
Source data are provided with this paper.

Research involving human participants, their data, or biological material

Policy information about studies with human participants or human data. See also policy information about sex, gender (identity/presentation), and sexual orientation and race, ethnicity and racism.

Reporting on sex and gender	not applicable for this study
Reporting on race, ethnicity, or other socially relevant groupings	not applicable for this study
Population characteristics	not applicable for this study
Recruitment	not applicable for this study
Ethics oversight	not applicable for this study

Note that full information on the approval of the study protocol must also be provided in the manuscript.

Field-specific reporting

Please select the one below that is the best fit for your research. If you are not sure, read the appropriate sections before making your selection.

☒ Life sciences ☐ Behavioural & social sciences ☐ Ecological, evolutionary & environmental sciences

For a reference copy of the document with all sections, see nature.com/documents/nr-reporting-summary-flat.pdf

Life sciences study design

All studies must disclose on these points even when the disclosure is negative.

Sample size	This study involved the measurements of one protein sample with different techniques. SEC-MALS and SAXS required the analysis of one sample (n=1), each data-point has an error that derives from the quality of the sample and the state of the optical equipment, the error translates into a standard deviation that affects the final interpolation. Structural analysis by cryo-EM was performed by averaging 2D projections of thousands particles (n indicated in the reporting table for each volume).
Data exclusions	No collected data was excluded in the analysis. The particles that were not used in the final cryo-EM reconstructions were selected by standard 2D and 3D classification steps implemented in cryoSPARC.
Replication	Biophysical characterization including SEC-MALS and SAXS analysis were repeated at least twice. The reported results are representative of the collected data.<br>Two cryo-EM data-sets were collected in similar conditions, the results were comparable and only the data-set that led to higher resolution reconstructions is reported in this study.<br>Two replicates of all atom MD simulations for each initial conformation were performed<br>Three replicates of coarse grained MD simulations for each initial conformation were performed

	All attempts at replication were successful.
Randomization	According to current practices in structural biology, no randomization was applied to the study.
Blinding	According to current practices in structural biology, no blinding was applied to the study.

Reporting for specific materials, systems and methods

We require information from authors about some types of materials, experimental systems and methods used in many studies. Here, indicate whether each material, system or method listed is relevant to your study. If you are not sure if a list item applies to your research, read the appropriate section before selecting a response.

Materials & experimental systems

n/a	Involved in the study	
☐	☒	Antibodies
☒	☐	Eukaryotic cell lines
☒	☐	Palaeontology and archaeology
☒	☐	Animals and other organisms
☒	☐	Clinical data
☒	☐	Dual use research of concern
☒	☐	Plants

Methods

n/a	Involved in the study	
☒	☐	ChIP-seq
☒	☐	Flow cytometry
☒	☐	MRI-based neuroimaging

Antibodies

Antibodies used	Anti-MVP antibody produced in rabbit - SIGMA - SAB5700906 (1:500)<br>Goat Anti-Rabbit IgG H&L (HRP) abcam - ab6721 (1:2000)
Validation	The antibody was used for western blots, the specificity was evaluated on denaturing SDS-PAGE with a secondary anti-rabbit antibody conjugated to HRP visible in Supplementary Figure 1a.

Plants

Seed stocks	not applicable for this study
Novel plant genotypes	not applicable for this study
Authentication	not applicable for this study
