# Supplementary material for: Structural flexibility of the human vault particle revealed by high-resolution cryo-EM and molecular dynamics simulations
Source: Nat Commun. 2026 May 2;17:6033. doi: 10.1038/s41467-026-72674-4 (PMC13347060; doi:10.1038/s41467-026-72674-4)
Supplement: Supplementary file 7 — Transparent Peer Review file [file 41467_2026_72674_MOESM7_ESM.pdf]

nature portfolio

Peer Review File

Structural flexibility of the human vault particle revealed by high-resolution cryo-EM and molecular dynamics simulations

Corresponding Author: Dr Fabio Lapenta

Editorial Note: Parts of this Peer Review File have been redacted as indicated to remove third party material where no permission to publish were obtained.

This file contains all reviewer reports in order by version, followed by all author rebuttals in order by version

Version 0:

Reviewer comments:

Reviewer #1

(Remarks to the Author)
The authors used cryo-EM on isolated human vault particles generated in the yeast Komagataella phaffii that does not possess vault-related genes, such as the major vault protein (MVP) or vault RNAs (vtRNAs). They solved the structures to resolutions between 3.09 - 4.45 A. The were able to identify two alternative conformational states of the human vault complex, dubbed primed (symmetric) and committed (asymmetric). The primed particles showed asymmetric structures mainly around the waist of the particle and molecular dynamics simulations suggest increased structural flexibility of the primed complex. The authors conclude that the primed structure of the complex is likely an on-pathway intermediate of the vault complex opening into halves.

Vault particles are mysterious and super large RNA-protein complexes whose cellular biology is poorly understood. Thus, research on this fascinating molecular assembly is important and relevant. Having said that, it has not escaped our attention that the gain of novel insights into vault complex physiology is quite limited in this submission.

1. It remains unclear to what extend the obtained structures are biological relevant. They used a heterologous system by expressing the human major vault protein (MVP) in a yeast that does not encode MVP, or the other two proteinaceous members (TEP1, vPARP), or the vault RNAs of the complex. While it has been shown decades ago that expression MVP alone is sufficient for vault complex formation, it is everything else but clear if and how any of the other genuine vault components might influence the confirmational dynamics of this complex. And in the end, this is the main goal of thus submission, insight into vault particle dynamics.
2. In the title 'human vault protein' is misleading since the authors do not study the MVP protein in isolation, but in the vault complex. Thus, it would be better to change to 'human vault particle'.
3. The term 'ubiquitous' (line 44) and the phrase 'nearly identical morphology across eukaryotes' (line 60) are at least misleading, because there are no vaults in some important eukaryotes including yeast, C. elegans, etc.
4. In line 68, the term 'vault-specific' may not be appropriate. vtRNAs are also found outside of the vault particle, and vault-independent vtRNA is thought to be the most abundant form I cells, performing key functional roles.
5. In the first paragraph of the result section, it would be good to mention that MVP alone is sufficient for vault particle formation, along with adding a reference #2 (Stephen, A. G. et al.).
6. If the authors can provide any comment on the factors that might influence the relative proportion of primed and committed states (even a brief discussion) shown in figure 1E, would strengthen the manuscript. It is not evident from the text or the data why the authors assume that the committed state of the particle is indeed a non-path intermediate to vault RNA halves.
7. Colors in figure 5A and 6A are not easily distinguishable, particularly purple for Na+. In addition, these panels (5A and 6A) do not seem essential to the main findings and could be moved to the supplementary figure.
8. In line 406, (Figure 6A) should be in bold.
9. Vault particles contain other proteins besides MVP, and vtRNAs. How does the presence of these components influence conformational changes or the overall structure? Indeed, vPARP can enter pre-assembled vaults via the INT domain (as the authors noted in the introduction-line 73), and other components of the vault particle including TEP1 and vtRNA may also contribute to the conformational state. From this perspective, it would be helpful to perform the structural analysis while adding these components one by one to better understand a conformational state of a more complete vault.
10. Lines 451-453: that is interesting! What kind of external stimuli have the authors in mind that could trigger the transition from the primed to the committed confirmation? Experimental evidence for such external stimuli would greatly enhance the significance of their findings.

Reviewer #2

(Remarks to the Author)
I co-reviewed this manuscript with one of the reviewers who provided the listed reports. This is part of the Nature Communications initiative to facilitate training in peer review and to provide appropriate recognition for Early Career Researchers who co-review manuscripts.

Reviewer #3

(Remarks to the Author)
NCOMMS-25-28919

Summary:

The manuscript by Lapenta et al. proposes the large vault ribonucleoprotein complex relies on MVP conformational changes for assembly and disassembly. Earlier structural work has revealed conformational changes within the N-terminal waist region; however the low resolution has prevented a detailed understanding of how the vault opens. Using cryoEM, the authors uncover both a symmetric and asymmetric state of the vault assembly. Moreover, the authors leverage molecular dynamics simulations to propose that the asymmetric state is committed to disassembly. This manuscript is very well written. While the results presented in this work are important to the field, there is key information missing and it is not clear whether this analysis is sufficiently noteworthy from earlier work (e.g. Guerra et al. (2022)).

Please find specific comments below.

1. The authors should explain the discrepancy in molecular mass. Their SEC-MALS experiment suggests the vault complex is predominantly assembled whereas their interpretation of the SAXS experiment is a near even distribution between assembled and disassembled complexes. The assumption that the SAXS sample is a mixture of assembled and disassembled vault RNP complexes does improve the overall fit (chi square value from 2.2 to 1.8); however, it does not fully explain the data. This is particularly evident in the lower q range. Is this SAXS scattering profile representative across protein concentrations? The authors should provide additional quality metrics for their SAXS data (e.g. scattering plots and their respective Guinier plots for three protein concentrations).

2. Supplementary figures 5 , 10, and 11 should include an orientation plot to demonstrate the diversity of orientations in their data.

3. Authors list hydrophobic and polar MVP residues involved in maintaining the primed state however it is not apparent that there is sufficient resolution. This is particularly important for the histidine rich interfacial contact where they claim there is a hydrogen bonding network. The authors should state the local resolution at these interfaces and take care to ensure there is sufficient resolution for their claims.

4. The authors should target key MVP interface residues observed in their cryoEM reconstruction for mutagenesis and assess whether disruption of these surfaces disfavor vault assembly in solution.

5. The current discussion is limited. The authors should provide an in depth analysis on how their findings advance our understanding of vault regulation and why they observed committed states and half shells by cryoEM, but no other intermediate states.

Reviewer #4

(Remarks to the Author)
This manuscript, “Structural flexibility of the human vault protein revealed by high resolution cryo-EM and molecular dynamics simulations”, is directed at an important problem in cell biology. The vault particle is an extremely stable, nano-scale particle, composed of a protein shell entirely composed of 78 copies of a single ~100 Kd protein (the major vault protein, MVP). However, fully assembled vaults have been shown, biochemically, to be very dynamic as they can internalize very large proteins (like PARP4 and TEP1) and even large particles (like 10 nm nano-discs and adeno associated virus).

The authors use cryo-EM and MD simulations in an attempt to reveal the structural factors that impart flexibility on the human vault. They describe two cryo-EM conformational states of the vault and MD simulations of these structures. They conclude that their data suggests a possible mechanism for the dynamic assembly and disassembly of the vault.

This review includes comments from an expert in molecular dynamics simulations who was included as an early career researcher (ECR) who co-reviewed this manuscript as part of the Nature Communications initiative to facilitate training in peer review and to provide appropriate recognition for Early Career Researchers who co-review manuscripts.

In general, this is an ambitious manuscript with a number of significant problems and some important minor issues as well.

First the vaults used in the analysis, which are expressed from human MVP in yeast, are only partially purified. Over 40% of

the particles that they obtain are half vaults. As half-vaults are (almost) never seen in in situ images of vaults in cells and rarely seen in highly purified vaults from eukaryotic tissues, it is likely that these "half" vaults are products of vault degradation or denaturation. How can we be assured that the second cryo-EM structure, the "committed" vaults, might just represent a class of damaged particles as well?

The MD simulations are quite disappointing, revealing minor structural variations that do not explain the enormous openings of the particle that must be occurring to explain the biochemical dynamics previously reported.
One minor, but significant point is the underlying incorrect assumption by the authors that the vault particles self-assemble. It was shown over ten years ago that vaults do not self-assemble, rather vaults are assembled on polyribosomes. It is likely that free MVP chains do not exist in cells and the notion that vaults in cells come apart into halves and can exchange halves has not been rigorously established.

With regard to the cryo-EM analysis, in lines 136–138, the authors state that they performed an unsupervised 3D classification of the particles and identified two major sub-populations, distinguished by the presence or absence of D39 point-group symmetry. However, the manuscript does not specify the relative proportions of particles assigned to each class. The authors should provide the percentage or particle counts for these two sub-populations. This information is important for evaluating the structural heterogeneity and the robustness of the classification.
The observation of both symmetric and asymmetric conformations suggests potential biological relevance beyond mere structural variability. While the symmetric state may reflect the most stable conformation of the vault particle, the manuscript would benefit from further discussion on the possible functional or mechanistic implications of the asymmetric state. Any relevant hypotheses or comparisons with prior studies would strengthen the interpretation.

A more detailed analysis of the MD simulations follows:

First, the authors should be commended for undertaking all-atom molecular dynamics simulations on such a large and complex system - this is indeed a significant technical effort and apparently, a first vault simulation in the field. However, the depth and scope of the results do not adequately support the conclusions drawn, especially considering the scale and ambition of the simulations. Here are a few major problems associated with the computational part of the paper.

1. Simulation time reporting and adequacy: The manuscript states: "We performed two replicates of AA-MD simulations for each initial conformation, each running for 250 ns, yielding a cumulative simulation time of 1 μs." This method of summing replicate durations to report total simulation time is misleading. Each individual trajectory spans only 250 ns, which is insufficient for capturing the full range of structural dynamics in systems of this size and complexity. At a minimum, simulations of at least 500 ns per replica would be expected to ensure adequate sampling of conformational space at all-atom scales since they do CG simulations as well. (https://www.nature.com/articles/s41586-024-07150-4)

2. Interpretation and strength of conclusions: Several statements in the manuscript appear speculative or overly interpretive relative to the data shown. For example:
"These fluctuations suggested the presence of a general breathing motion."
"Both the cap and helix regions displayed smaller diameter fluctuations than the waist, supporting the hypothesis that the vault's greater flexibility at the waist is closely linked to its opening mechanism."
While these are interesting hypotheses, they would be more compelling if supported by more experimental data or prior simulation studies demonstrating vault opening behavior. In the absence of such evidence, the claims should be clearly framed as speculative or proposed interpretations. Additionally, if the proposed breathing motion is functionally meaningful, one might expect to see correlated solvent or ion exchange dynamics—features that are not currently analyzed. Drawing parallels with well-characterized systems like nucleosomes (which also exhibit breathing) may offer useful context but should be supported by analogous data. Overall, these interpretations should be tempered and more clearly distinguished between observations and hypotheses.

3. Underwhelming analysis of all-atom simulations: The manuscript presents only RMSD as an output from the all-atom simulations, which is a surprisingly limited use of what should be a rich dataset. All-atom MD provides access to a wide range of structural, dynamic, and thermodynamic observables - far beyond RMSD. Analyses such as ion/water exchange, hydrogen bonding patterns, local flexibility profiles, and correlated motions could provide valuable insights, especially for a system as complex as the vault. Additionally, equilibration of such systems is typically evaluated through stabilization of water/ion exchange or other internal metrics, not merely through RMSD plateaus. The authors should look into details of those simulations and show results indicative of breathing. The authors are encouraged to incorporate more robust metrics and comparative analyses, as demonstrated in the following studies:
https://pubs.acs.org/doi/full/10.1021/acs.jctc.3c00846,
https://www.nature.com/articles/s41586-024-07150-4,
https://www.science.org/doi/10.1126/science.adq7597
These works highlight best practices in leveraging all-atom simulations to gain mechanistic insight, and the current manuscript would benefit from adopting similar approaches.

4. The manuscript states: "In the MD simulation trajectories, the vault underwent significant breathing motions in solution, yet the particle did not transition between the two conformations, nor did the two halves initiate the opening in the absence of external stimuli." After careful review of the trajectory data, including extended viewing of the provided simulation videos at multiple playback speeds, supplementary, this claim of "significant breathing motions" does not appear to be convincingly supported. While some local fluctuations are expected in such large systems, the overall structural changes depicted in the simulation videos appear subtle and well within the range of thermal noise. Without quantitative metrics - such as internal

volume fluctuations, inter-subunit distance variation, or principal component analysis - it is difficult to substantiate the use of the term "significant." The authors are encouraged to provide more rigorous, quantifiable evidence to support this observation to better reflect the modest level of motion observed in the simulations.

5. The manuscript includes statements such as: "These pores could serve as a mechanism for pressure release and passive diffusion of ions and small molecules.", "The vault's attractiveness as self-assembling human nanoparticles... emphasizes the importance of understanding their dynamics."
While these are intriguing hypotheses, the authors should revisit the relevant literature and more accurately contextualize these claims. For instance, the term "self-assembling human nanoparticles" is wrong. Furthermore, their use in applications such as AAV (virus) trapping is ongoing research and should be framed accordingly (https://www.biorxiv.org/content/10.1101/2023.11.29.569229v4). These claims require clearer language and proper experimental support. The authors should revisit the literature thoroughly—this is not optional.

6. The methods section refers to multiple pH values across different experimental steps, yet it does not specify the pH at which the protein was protonated for the all-atom MD simulations. Was it pH 7, 7.5, or 8? Given the pH sensitivity of protein protonation states and their influence on dynamics and electrostatics, this omission is critical and should be addressed explicitly.

Reviewer #5

(Remarks to the Author)
This manuscript focuses on the vault ribonucleoprotein complexes, reporting cryo-EM structures of both primed and committed conformations (of complete vault and half-vault particles), as well as all-atom and coarse-grain simulations of these systems. Although previous work has reported cryo-EM reconstructions of these conformational states of vault, this appears to be the first time that all-atom simulations of the intact vault complex have been reported. Given that the explicitly solvated system contains 18.4 million atoms, these simulations represent a serious investment of supercomputer time and an extremely valuable dataset to investigate the detailed dynamics of the complex vault assembly. However, the only analyses performed on the all-atom trajectory are root-mean-squared-deviation (RMSD), root-mean-squared fluctuation (RMSF), and ion permeability – this is an serious lost opportunity.

Since the cryo-EM structure analysis focus on per-residue contacts, it would be very interesting to conduct a similar analysis on the all-atom MD trajectory, to see how many of the contacts captured by cryo-EM persist during MD and with what frequency. Protein dynamics are essential to function. Clearly the vault complex is a highly dynamic system, such that it is best described by ensembles of conformers that define its conformational states. MD provides insights into these ensembles not available by other structural biology techniques. It would be a shame not to extract this type of data from the simulation trajectory given the notable effort and computational expense of obtaining it. The simulations of intact vault are relatively short (2 x 250 ns each), but data from symmetric copies can be combined to obtain a large amount of cumulative sampling for these local dynamics (e.g. 39 copies of MVP x 2 x 250 ns = 19.5 microseconds).

Beyond residue contact frequencies, MD simulations also allow characterization of the collective motions and emergent dynamics of the assembly that are also relevant for function. The authors refer several times to "breathing motions" but do not characterize the collective motions that truly constitute "breathing'. They could use PCA or related dimensionality-reduction approaches to characterize distinct modes of vault motion, or other correlated emergent dynamics such as acoustic modes. For example, see Perilla & Schulten (https://www.nature.com/articles/ncomms15959) for examples of such analyses applied to large, intact protein shells. Examining collective motions from all-atom MD trajectories can provide mechanistic insights, and this aspect is not exploited in the manuscript in its current form.

Why not also do the ellipse-diameter analysis on the all-atom trajectory? Changes in not only elliptical diameter along the vault waist but also cross-sectional area of the ellipse could be correlated with changes in per-residue contacts along the waist to provide valuable information regarding the molecular determinants of this interface that represents the seam between the two vault halves, which apparently separate to encapsulate cargo.

The authors conclude, based on larger RMSF values for the committed conformation relative to the primed conformation, that the committed conformation is less stable. However, there is something of a chicken versus egg problem here. The authors attribute lower resolution of the committed conformation map to the larger fluctuations in dynamics observed with MD. But Singharoy & Schulten (e.g. https://elifesciences.org/articles/16105) have previously shown that structural instability detected by RMSF can be a product of the low-resolution model used as initial coordinates for the MD. In this way, large overall RMSF of a structure can be indicative of the quality of the fit of the model to the EM map it was derived from, rather than validation of the dynamics of the ensemble of conformers averaged into the reconstruction. There may be no way to know based on the available data which is true here (if the structure is very flexible in MD because the map is low resolution, or if the map is low resolution because the real-life structure of the assembly is flexible), but the authors could be careful to at least caveat this rather than presenting it as a firm conclusion. Finally, flexibility does not necessarily mean instability.

Overall, this is excellent work but appears rather incomplete because very little analysis was performed on the most unique dataset presented – the MD simulations. The authors should do a bit more with these MD trajectories to bring the work full circle, relating MD to the cryo-EM data in a more detailed and precise way and seeing what can then be inferred mechanistically from these details that are relevant to the opening/closing mechanism of vault. The authors should keep in

mind that the EM maps/structures will be deposited and are available to researchers to analyze in detail on their own for follow-up studies. However, MD simulations particularly of this size are typically not released and most structural biology researchers lack the computing skills to conduct analysis; as such, readers rely entirely on the authors to distill the value from this remarkable large-scale trajectory. There is no double at incredible amount of valuable information in these MDs that must be brought to light to learn novel insights about vault not accessible to experimental methods.

Reviewer #6

(Remarks to the Author)
I co-reviewed this manuscript with one of the reviewers who provided the listed reports. This is part of the Nature Communications initiative to facilitate training in peer review and to provide appropriate recognition for Early Career Researchers who co-review manuscripts.

Version 1:

Reviewer comments:

Reviewer #1

(Remarks to the Author)
The revised version submitted by Lapenta et al. has addressed most of our points raised during the first review round. In particular, all suggested textual changes for improving the clarity of the manuscript have been adequately conducted. The most central point of critique by us (and as it seems also by the other referees), namely to convince the reader that indeed physiologically relevant vault complex structures were studied, has unfortunately only been partially addressed. We particularly appreciate the conducted MVP mutants located between the vault halves region and their subsequent structural analyses by TEM (Suppl. Fig. 27). All other suggested experiments that would improve the biological relevance of the structures (e.g. (i) studying also the remaining vault complex components and their contribution to the proposed conformational dynamics; (ii) quantifying the ratio of primed and committed states under different stresses such as temperature, salt concentration or pH) were considered “beyond the scope of the present revision”. While this is a fair and justified argument, consequently however, the physiological relevance of the presented vault structures has, in essence, not improved much in the revised version of the manuscript.

Reviewer #2

(Remarks to the Author)
I co-reviewed this manuscript with one of the reviewers who provided the listed reports. This is part of the Nature Communications initiative to facilitate training in peer review and to provide appropriate recognition for Early Career Researchers who co-review manuscripts.

Reviewer #3

(Remarks to the Author)
NCOMMS-25-28919A-Z

The authors have revised the manuscript by Lapenta et al. to address the concerns and suggestions raised during the review. This revised manuscript now reports new SAXS data and analysis, resolving the earlier discrepancy between their SAXS and SEC-MALS studies. Authors provide additional insight into the global/local quality and limitations of their 3D reconstructions by offering additional figures and text revisions. The authors now perform targeted site directed mutagenesis, validating their structural data and offering important insight into the role of discrete residues in vault assembly. I believe the additional data and updated discussion have greatly improved this study. I support publication of this work.

Reviewer #4

(Remarks to the Author)
The authors have done an adequate job in revising this manuscript and I have no further criticisms.

Reviewer #5

(Remarks to the Author)
The authors have addressed my previous concerns.

Reviewer #6

(Remarks to the Author)
I co-reviewed this manuscript with one of the reviewers who provided the listed reports. This is part of the Nature Communications initiative to facilitate training in peer review and to provide appropriate recognition for Early Career Researchers who co-review manuscripts.

Open Access This Peer Review File is licensed under a Creative Commons Attribution 4.0 International License, which permits use, sharing, adaptation, distribution and reproduction in any medium or format, as long as you give appropriate credit to the original author(s) and the source, provide a link to the Creative Commons license, and indicate if changes were made.
In cases where reviewers are anonymous, credit should be given to 'Anonymous Referee' and the source.
The images or other third party material in this Peer Review File are included in the article's Creative Commons license, unless indicated otherwise in a credit line to the material. If material is not included in the article's Creative Commons license and your intended use is not permitted by statutory regulation or exceeds the permitted use, you will need to obtain permission directly from the copyright holder.
To view a copy of this license, visit https://creativecommons.org/licenses/by/4.0/

We sincerely thank all reviewers for handling our manuscript and for the insightful, constructive, and thorough feedback, which has greatly strengthened this manuscript. The reviewers consistently emphasized the need to fully exploit the rich cryo-EM and molecular dynamics datasets, particularly in validating the biological relevance of the observed conformational states and strengthening the mechanistic interpretation of vault dynamics. Key requests included: (1) enhanced analysis of the cryo-EM data; (2) re-evaluation of SAXS data with additional quality metrics; (3) deeper integration of MD simulations with structural observations, including PCA to characterize collective motions, residue-level contact persistence, and solvent dynamics; and (4) experimental validation of structural observations through targeted mutagenesis of key interfacial residues.

In response, we have implemented comprehensive revisions:
(1) Performed PCA and orientation analysis on cryo-EM data, confirming the structural integrity and non-artifactual nature of the committed state (new Supplementary Figures 6-8, 14-16).
(2) Re-analyzed SAXS data with additional quality metrics and concentration-dependent validation, resolving discrepancies in the assembly state distribution (new Supplementary Figure 2 and new Supplementary Table 1).
(3) Expanded MD analysis with per-residue contact mapping, solvent exchange, and flexibility profiling (updated Figure 5, new Supplementary Figures 19-24 and new Movie 3).
(4) Conducted MD-guided mutagenesis experiments targeting key interfacial residues pinpointed by MD simulations (e.g., I36D, D39A/R42A), demonstrating their role in vault stability (Supplementary Figure 27).

These changes ensure a more rigorous, transparent, and biologically grounded interpretation of the vault's dynamic architecture.

Below, we have repeated the comments verbatim and our responses in regular font. Changes to the manuscript are discussed in *underlined italic text*.

Reviewers' comments:

Reviewer #1 (Remarks to the Author):

The authors used cryo-EM on isolated human vault particles generated in the yeast Komagataella phaffii that does not possess vault-related genes, such as the major vault protein (MVP) or vault RNAs (vtRNAs). They solved the structures to resolutions between 3.09 - 4.45 A. The were able to identify two alternative conformational states of the human vault complex, dubbed primed (symmetric) and committed (asymmetric). The primed particles showed asymmetric structures mainly around the waist of the particle and molecular dynamics simulations suggest increased structural flexibility of the primed complex. The authors

conclude that the primed structure of the complex is likely an on-pathway intermediate of the vault complex opening into halves.

Vault particles are mysterious and super large RNA-protein complexes whose cellular biology is poorly understood. Thus, research on this fascinating molecular assembly is important and relevant. Having said that, it has not escaped our attention that the gain of novel insights into vault complex physiology is quite limited in this submission.

1. It remains unclear to what extend the obtained structures are biological relevant. They used a heterologous system by expressing the human major vault protein (MVP) in a yeast that does not encode MVP, or the other two proteinaceous members (TEP1, vPARP), or the vault RNAs of the complex. While it has been shown decades ago that expression MVP alone is sufficient for vault complex formation, it is everything else but clear if and how any of the other genuine vault components might influence the confirmational dynamics of this complex. And in the end, this is the main goal of thus submission, insight into vault particle dynamics.

Reply: We agree with the reviewer that the presence of other molecular partners (TEP1, vPARP, and vtRNAs) might alter the conformational dynamics of the vault in vivo. For this reason, our study was intentionally designed to isolate the structural plasticity of the MVP shell itself. As the reviewer mentioned, MVP alone is sufficient to form stable vaults and used in synthetic biology for cargo encapsulation and delivery has been explored (Kickhoefer et al., 2005; Han et al., 2011; Kar et al., 2012; Collins et al., 2024; Chao et al., 2026; among others). We therefore interpret our results as an MVP-intrinsic baseline that disentangles the intrinsic properties of the MVP-based vault from those potentially introduced by additional components.

Importantly, we do not claim that the MVP-only conformational landscape fully captures native vault behavior in cells. We have revised the Discussion to state this limitation explicitly and to frame our conclusions as an MVP-intrinsic baseline. At the same time, this baseline enables direct mechanistic tests (see new mutagenesis and MD analyses below) that would be more difficult to interpret unambiguously in a heterogeneous reconstituted system or cellular context.

Future studies reconstituting the complete complex, including TEP1, vPARP, and vault RNAs, will be essential to obtain a comprehensive understanding of vault dynamics under physiological conditions. We have revised the manuscript to clarify this point, explicitly acknowledge the limitations of our system, and cite a recent publication describing the co-expression of the vault with vPARP (doi: 10.1038/s41467-025-61981-x).

New text included in the Discussion section of the amended manuscript at line 518:

> *pp. 13-14: "…it is important to note that while MVP alone constitutes the core molecular architecture of the vault particle, the vault's additional components (TEP1, vPARP, and vault RNAs) may significantly affect its conformational landscape in vivo. Accordingly, the conformational heterogeneity and dynamics quantified here should be interpreted as an MVP-intrinsic baseline rather than a complete description of native vault behaviour. Future work reconstituting the complete complex will be critical to fully capture vault dynamics under physiological conditions."*

2. In the title 'human vault protein' is misleading since the authors do not study the MVP protein in isolation, but in the vault complex. Thus, it would be better to change to 'human vault particle'.

Reply: We thank the reviewer for the suggestion and changed the title of the manuscript that now reads:

> _p 1: "Structural flexibility of the human vault particle revealed by high-resolution cryo-EM and molecular dynamics simulations"_

3. The term 'ubiquitous' (line 44) and the phrase 'nearly identical morphology across eukaryotes' (line 60) are at least misleading, because there are no vaults in some important eukaryotes including yeast, C. elegans, etc.

Reply: We thank the reviewer for pointing this out. We have revised the manuscript to remove the term "ubiquitous" and to rephrase the description of vault distribution in eukaryotes. The revised text clarifies that vaults are present in many, but not all, eukaryotes, and explicitly notes reported absences in certain eukaryotes:

> _p.2: "With notable exceptions (including fungi, insects and likely plants), vault's phylogenetic distribution is widespread across the major eukaryotic supergroups and possibly present already in the last eukaryote common ancestor (LECA)[13,14]"_

4. In line 68, the term 'vault-specific' may not be appropriate. vtRNAs are also found outside of the vault particle, and vault-independent vtRNA is thought to be the most abundant form I cells, performing key functional roles.

Reply: We have amended the manuscript to avoid the term "vault-specific" when referring to vtRNAs. The revised text now acknowledges that vtRNAs also exist outside of the vault particle and that vault-independent vtRNA likely represents the predominant state (line 54):

> _p.2: "… Vaults are associated with short non-coding vault RNAs (vtRNA), which are not restricted to the vault and are also found in the cytosol[31,25]."_

5. In the first paragraph of the result section, it would be good to mention that MVP alone is sufficient for vault particle formation, along with adding a reference #2 (Stephen, A. G. et al.).

Reply: We revised the text in the first paragraph of the Results, that now reads:

> _p.3: "Assembly of MVP on the polyribosome has been observed[50] and MVP alone is sufficient for vault particle formation[2]."_

6. If the authors can provide any comment on the factors that might influence the relative proportion of primed and committed states (even a brief discussion) shown in figure 1E, would strengthen the manuscript. It is not evident from the text or the data why the authors assume that the committed state of the particle is indeed a non-path intermediate to vault RNA halves.

Reply: We thank the reviewer for raising this point. To address it, we have included a brief discussion of factors that may influence the relative proportion of primed and committed states in the revised manuscript at line 524.

> *p. 17: "The proportion of primed and committed conformations may be influenced by the particle's intrinsic dynamics and environmental factors. The vault shell undergoes continuous breathing motions that enable sampling of multiple conformations and likely favour the more flexible committed state. The committed conformation was previously proposed to represent an on-path intermediate in the disassembly of the full vault into half vaults due to prominent structural alterations in some R1-R1 interactions. However, our mutational analysis indicated that disrupting specific hydrophobic interactions at the waist was required to destabilize the full vault and dramatically drive the formation of half vaults. Further studies will be required to fully elucidate the factors controlling the conformational space of vaults. In particular, it may be interesting to explore how the ratio of the primed and committed states is influenced by environmental factors such as temperature, salt concentration and pH, which were previously noted to have a profound effect on the stability of the vault[37,39]."*

Regarding the possibility that the committed form represents an on-path intermediate toward vault opening, we share the reviewer's caution. While we do not claim to confirm the mechanism proposed by Guerra et al. (2022), we highlight that the most significant structural differences between the primed and committed states are localized at the waist, the region responsible for inter-halves stability. Disruption of interactions in this region due to point mutations lead to vault opening, suggesting this region is a functional hotspot. See point 4 in Reviewer 3's comments for details.

7. Colors in figure 5A and 6A are not easily distinguishable, particularly purple for Na+. In addition, these panels (5A and 6A) do not seem essential to the main findings and could be moved to the supplementary figure.

Reply: We agree with the reviewer. To improve clarity and focus, we have removed panel 5A from the main text. We have also added new simulation data and analysis (see below) and moved the previous Figure 6 to the supplementary information and split the original Figure 5 into two new figures: Figure 5 and Figure 6.

8. In line 406, (Figure 6A) should be in bold.

Reply: The revised manuscript now includes this correction.

9. Vault particles contain other proteins besides MVP, and vtRNAs. How does the presence of these components influence conformational changes or the overall structure? Indeed, vPARP can enter pre-assembled vaults via the INT domain (as the authors noted in the introduction-line 73), and other components of the vault particle including TEP1 and vtRNA may also contribute to the conformational state. From this perspective, it would be helpful to perform the structural analysis while adding these components one by one to better understand a conformational state of a more complete vault.

Reply: We agree with the reviewer that stepwise reconstitution (adding vPARP, TEP1, and vtRNAs one-by-one) would be an excellent strategy to quantify how each component reshapes the vault's conformational equilibrium, and we view our work as an important first step in that direction. However, performing such structural analyses is beyond the scope of the present revision, because it requires establishing reproducible co-expression or reconstitution protocols with controlled stoichiometry and occupancy, and generating sufficiently homogeneous samples to support robust 3D classification across conditions and extensive simulation data.

Our study therefore focuses on MVP-only assemblies to establish a well-defined reference conformational ensemble (see reply to Reviewer 1, comment 1). We have added this explicitly in our discussion:

> p 14: "*Future work reconstituting the complete complex will be critical to fully capture vault dynamics under physiological conditions with bound cargo.*"

10. Lines 451-453: that is interesting! What kind of external stimuli have the authors in mind that could trigger the transition from the primed to the committed confirmation? Experimental evidence for such external stimuli would greatly enhance the significance of their findings.

Reply: We thank the reviewer for this question. Prior work has shown that chemical and mechanical stimuli can destabilize the vault shell and induce local fractures/opening including low-pH and AFM tip-probing (Llauró et al., 2016). This has been also confirmed by our own work (see Supplementary Figure 27 and reply to Reviewer 3, point 4). These observations suggest that vault conformational equilibria can be shifted by environmental conditions and/or applied forces, but it remains unclear whether these perturbations correspond specifically to the primed to committed transition observed here, and whether such stimuli occur in vivo.

We have further clarified this point in the manuscript at line 531:

> *p 14: "… further studies will be required to fully elucidate the disassembly. In particular, it may be interesting to explore how the ratio of the primed and committed states is influenced by environmental factors such as temperature, salt concentration and pH, which were previously noted to have a profound effect on the stability of the vault[37,39]."*

Reviewer #2 (Remarks to the Author):

I co-reviewed this manuscript with one of the reviewers who provided the listed reports. This is part of the Nature Communications initiative to facilitate training in peer review and to provide appropriate recognition for Early Career Researchers who co-review manuscripts.

Reply: We thank the reviewer for taking the time to revise the manuscript.

Reviewer #3 (Remarks to the Author):

Summary:

The manuscript by Lapenta et al. proposes the large vault ribonucleoprotein complex relies on MVP conformational changes for assembly and disassembly. Earlier structural work has revealed conformational changes within the N-terminal waist region; however the low resolution has prevented a detailed understanding of how the vault opens. Using cryoEM, the authors uncover both a symmetric and asymmetric state of the vault assembly. Moreover, the authors leverage molecular dynamics simulations to propose that the asymmetric state is committed to disassembly. This manuscript is very well written. While the results presented in this work are important to the field, there is key information missing and it is not clear whether this analysis is sufficiently noteworthy from earlier work (e.g. Guerra et al. (2022)).

Please find specific comments below.

1. The authors should explain the discrepancy in molecular mass. Their SEC-MALS experiment suggests the vault complex is predominantly assembled whereas their interpretation of the SAXS experiment is a near even distribution between assembled and disassembled complexes. The assumption that the SAXS sample is a mixture of assembled and disassembled vault RNP complexes does improve the overall fit (chi square value from 2.2 to 1.8); however, it does not fully explain the data. This is particularly evident in the lower q range. Is this SAXS scattering profile representative across protein concentrations? The authors should provide additional quality metrics for their SAXS data (e.g. scattering plots and their respective Guinier plots for three protein concentrations).

Reply: We thank the reviewer for pointing out the inconsistency between our SEC-MALS results and our initial SAXS interpretation. We agree that the previous SAXS-based estimate of a substantial full-/half-vault mixture was under-constrained and sensitive to modeling assumptions.

To resolve this, we performed additional SAXS measurements across a concentration series (0.65–2.6 mg/mL) and now provide the requested SAXS quality metrics, including scattering profiles and corresponding Guinier plots for three concentrations (new Supplementary Fig. 2). We observe no detectable concentration-dependent effects over this range.

We then re-estimated the molecular weight (Mw) using model-independent approaches that are better suited for very large particles (Rambo & Tainer, *Biopolymers,* 2011). Because the Guinier region for MVP is narrow (qRg < 1.3) and contains only a small number of points, Mw estimates from Guinier analysis alone carry relatively large uncertainty (Korasick, et al., *Protein Science,* 2018). We therefore determined Mw primarily from the Porod volume, which is appropriate for large macromolecular assemblies and typically yields <10% uncertainty (Rambo & Tainer, *Biopolymers,* 2011), and we used Guinier-based Mw as an orthogonal cross-check. The Mw values derived from the Porod volume are consistent across the concentration series and agree with the SEC-MALS mass (new Supplementary Table 1), indicating that half-vaults are present in solution in a small amount under our conditions.

Finally, we revisited our earlier two-component SAXS fitting. In light of the concentration-series and model-independent Mw estimates, we conclude that the previously improved χ^2 upon including half-vault scattering likely reflected model flexibility/overfitting, particularly in the low-q region where small systematic deviations can dominate the fit. We have updated the main text and supplementary material accordingly and now report the concentration-series SAXS analysis and quality controls in the new Supplementary Fig. 2 and new Supplementary Table 1.

*p.3: Batch small angle X-ray scattering (SAXS) analysis confirmed the assembled 78-mer assumes the expected shape in solution (**Supplementary Figures 2 and 3**). Moreover, estimation of molecular weight from SAXS data confirmed a small amount of half vaults (**Supplementary Table 1**).*

Supplementary Table 1. SAXS data analysis. *Model free parameters extracted from collected SAXS curves. Expected Mw from SEC-MALS measurements is 7.66 MDa, while the expected Rg, calculated from the cryo-EM structure of the MVP in the primed conformation is 23 nm. Mw and Rg values obtained by SAXS analysis are in reasonable agreement with expected values, indicating MVP is well folded in solution.*

**MVP sample***	**I(0) from Guinier#**	**Rg from Guinier# (nm)**	**Dmax (nm)**	**Mw from Porod volume (MDa)**	**Porod exponent**
merged	6.0 ± 0.9	23 ± 5	65	8.6	2.9
2.6 mg/ml	6.5 ± 0.5	31 ± 4	73	9.3	3.7
1.3 mg/ml	6.5 ± 0.3	30 ± 4	76	8.0	3.9
0.65 mg/ml	6.5 ± 0.6	29 ± 5	75	8.5	4.0

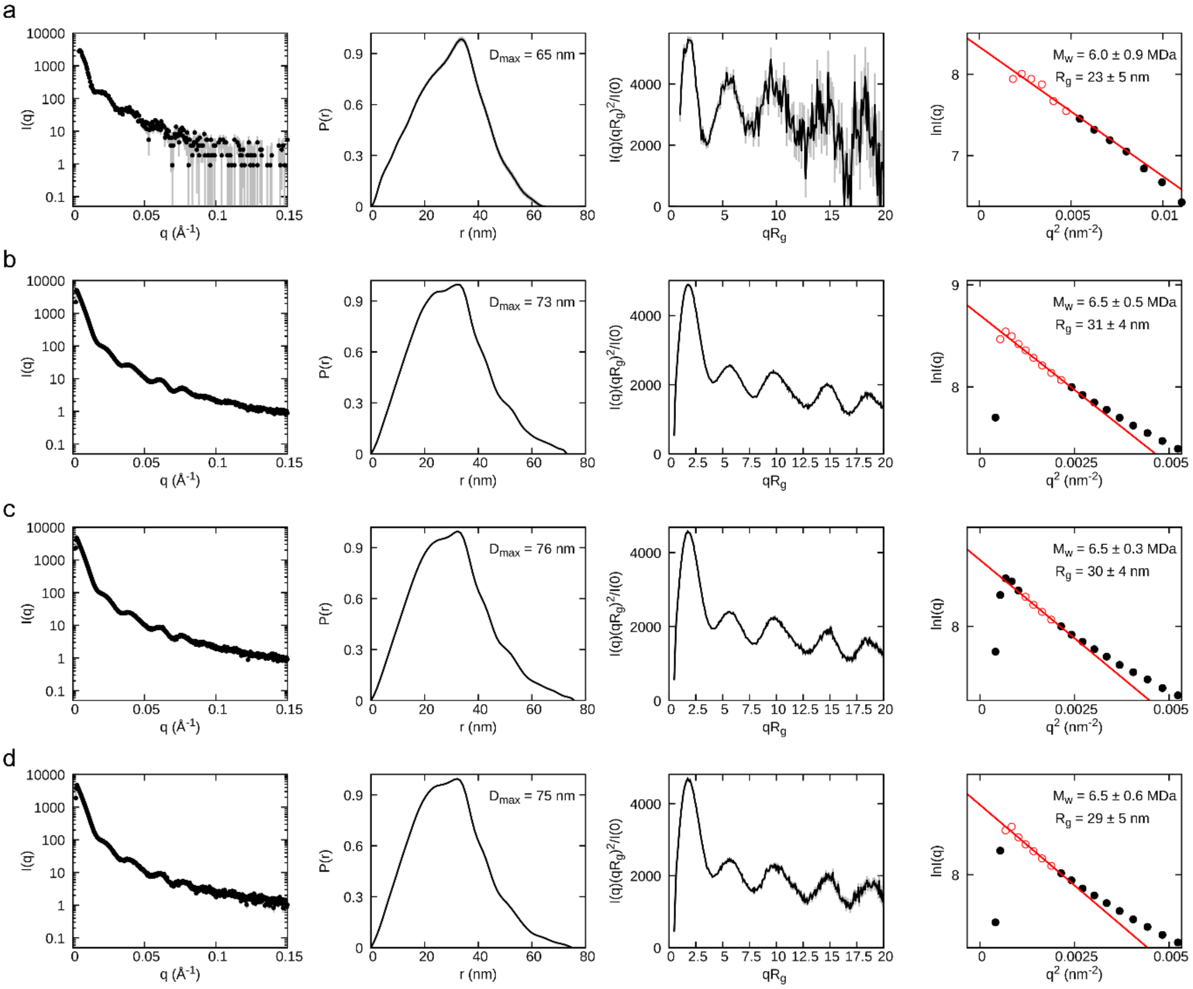

Supplementary Figure 2 – SAXS analysis of MVP particles in solution. *From left to right scattering curves, pair-distance distribution functions, Kratky plots and Guinier plots are shown for two independent SAXS samples. (a) Plots generated from a scattering curve obtained by merging two measurements carried out at concentration of 0.5 and 0.3 mg/ml at the BM29 BioSAXS beamline at ESRF, EMBL Grenoble. (b-d) Plots obtained by analyzing a concentration series ((b) 2.6 mg/ml, (c) 1.3 mg/ml and (d) 0.65 mg/ml) measured at the P12 beamline at DESY, EMBL Hamburg.*

2. Supplementary figures 5 , 10, and 11 should include an orientation plot to demonstrate the diversity of orientations in their data.

Reply: We thank the reviewer for suggesting the addition of orientation plots to evaluate the diversity of orientations in the dataset. The revised manuscript now includes viewing-direction/orientation distribution plots for the relevant reconstructions, demonstrating the diversity of particle orientations and sampling coverage (Supplementary Figures 7, 8, 14-16) as follows:

i) Direction distribution plot

ii) Fourier sampling plot (Sampling Compensation Factor)

iii) Particle scale factor vs. viewing direction

iv) Relative signal amount total and relative signal in elevation regions (from toroidal FSC)
v) Conical FSC at two different cone angle degrees

This more thorough analysis corroborated that the quality of the map for the vault in primed conformation is significantly better resolved than the committed. We attributed this difference to the presence of a significant degree of heterogeneity in the specific region of the committed vault at lower local resolution already described in the manuscript. Accordingly, we limited our claims on the atomic reconstruction of committed conformation, especially when describing the region at lower resolution at the waist. Similar conclusions were inferred for the map of the half vault. A detailed discussion of the new analysis was included in a Supplementary Discussion.

3. Authors list hydrophobic and polar MVP residues involved in maintaining the primed state however it is not apparent that there is sufficient resolution. This is particularly important for the histidine rich interfacial contact where they claim there is a hydrogen bonding network. The authors should state the local resolution at these interfaces and take care to ensure there is sufficient resolution for their claims.

Reply: In the revised manuscript we included a figure illustrating the local resolution and the distance of the interacting residues at the lateral MVP:MVP interface and the antiparallel interface, especially where we propose a hydrogen bonding network. We revised the main text and added a new Supplementary Figure 13 to ease the understanding:

> p.5: "*Analysis of lateral contacts identified several ionizable residues involved in MVP:MVP interaction in three clusters along the repeat domains at R1 (Arg9:Gln21), R5-6 (Asn234:Glu257:Thr245, Gln298:Glu305) and R8-9 (Asp383:Arg474, Arg461:Glu492, Lys476:Glu485), at the shoulder domain (Asp530:His534:His592, Arg536:Glu644) and in the helix cap (Glu700:Ser713, Arg729:Glu736, Arg766:Glu769). Taken together with the antiparallel interactions between Asp39:Arg42 and the triad Asn22:Gln38:Asn40, protonation-induced changes in this network of polar and ionic interactions is a possible cause of the pH-dependent destabilization of the vault observed in other works[37,38] (***Supplementary Figure 13***).*"

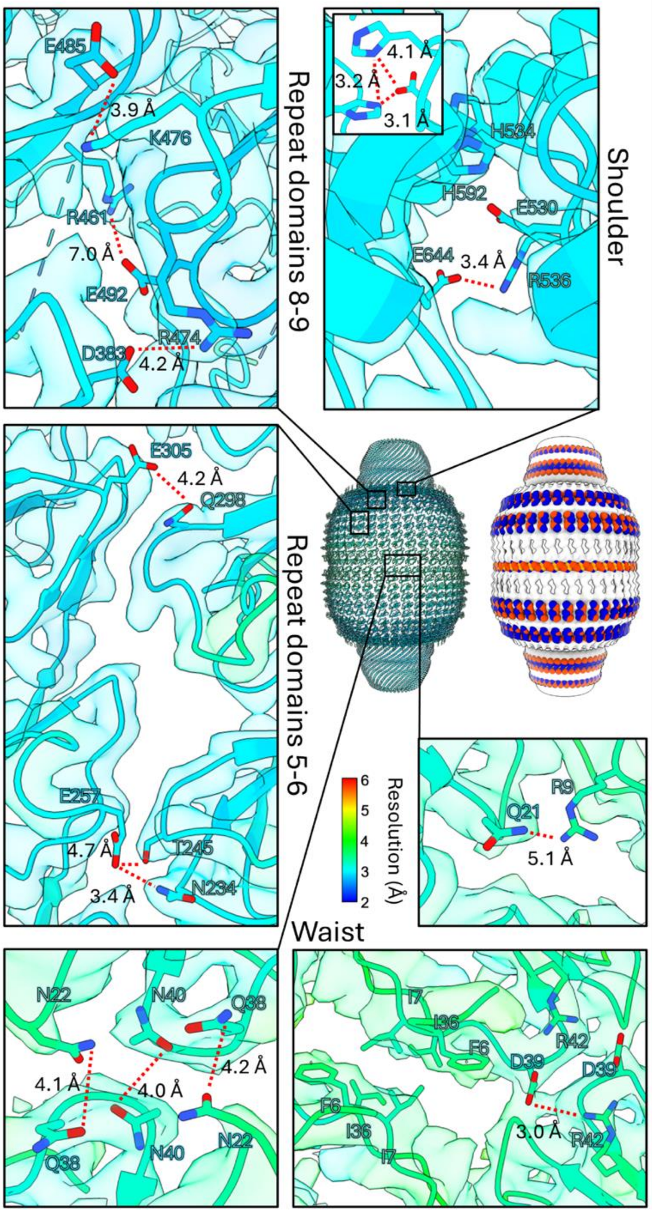

Supplementary Figure 13 – MVP:MVP contacts. Regions of contacts between MVP monomers are displayed in the figure with whole atomic model next to the cryo-EM density map (downscaled) as reference. The positions of the amino acids involved in the main MVP:MVP contacts are displayed on the density map as spheres colored either in red and blue or orange for polar interactions and hydrophobic, respectively. Inserts display the atomic model of the vault in primed conformation superimposed to the cryo-EM sharpened map (contour level between 0.7 and 1.2) with resolution shown in color (rainbow scale), with labels for the main residues taken into consideration and lines that measure the interatomic distance.

Furthermore, analysis of persistent residue-residue contacts during MD simulations supports the presence of such hydrogen bonding network:

> *p.9: "Analysis of persistent residue-residue contacts during MD simulations (**Figure 5C**) confirmed the stability of key interaction networks in both conformations. Long-lived contacts were conserved at the horizontal waist (inter-half contacts) and lateral MVP interfaces (intra-half contacts), validating the cryo-EM-derived atomic models. At the waist, the inter-half interface maintained stable polar interactions (N22:Q38, N40:Q38, D39:R42 salt bridge) and a dominant hydrophobic interaction (I36:I36). In lateral regions, MVP:MVP polar contacts predominated (e.g., R9:Q21, N234:E257, D383:R474), with additional contacts summarized in **Supplementary Figures 22** (lateral) and **23** (horizontal)."*

4. The authors should target observed in their cryoEM reconstruction for mutagenesis and assess whether disruption of these surfaces disfavor vault assembly in solution.

Reply: We thank the reviewer for this excellent suggestion. In response, we designed and tested a set of targeted mutations at interfaces that our cryo-EM models and MD analyses identified as candidate “hot spots” for stabilizing vault assembly in solution. Specifically, our **simulation contact analysis highlighted** a small set of **long-lived inter-half interactions at the waist**.

We generated MVP(Q38A,N40A) and MVP(D39A,R42A), designed to disrupt the N22/Q38/N40 network and the D39–R42 salt bridge. To challenge the hydrophobic patch, we generated MVP(I36D), introducing a charged residue into the **I36–I36 contact that showed a high persistence in the simulations** (see updated Fig. 5). **Strikingly**, among these variants, the **I36D mutation** produced the most pronounced effect on the vault assembly: it strongly **destabilized inter-half** association and **led to** a marked **enrichment of half-vault species in solution**, directly supporting the waist interface as a key energetic bottleneck for vault opening/assembly (New Supplementary Fig. 27).

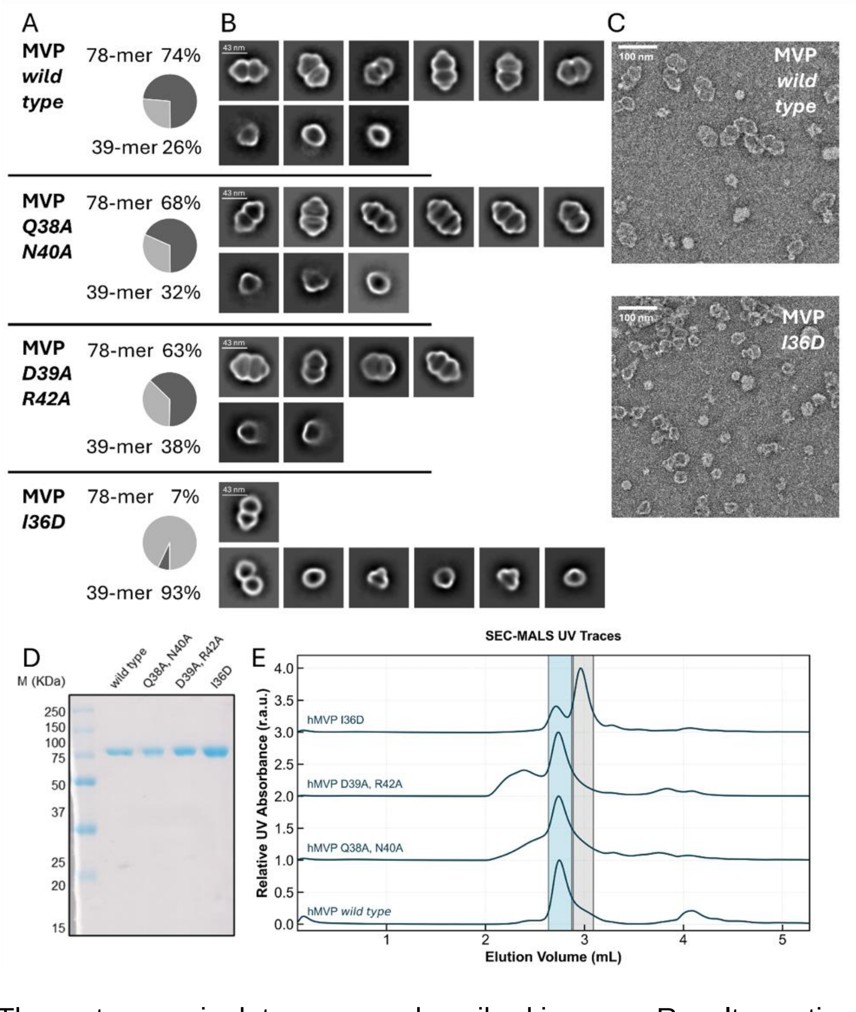

Supplementary Figure 27. MVP variants. A) Name of the MVP variants and ratio of fully assembled vaults (78-mer) and halves (39-mer) quantified with 2D classification on negative-stain TEM micrographs. B) Representative 2D class averages for each MVP sample used for the quantification. C) Representative micrographs for three samples (name indicated in the figure). D) SDS-PAGE of the samples after purification (the arrow indicates the band corresponding to MVP). E) SEC-MALS relative UV traces for each sample. The two sections highlighted in cyan and grey in the chromatographs correspond to 78-mer and 39-mer, respectively.

The mutagenesis data are now described in a new Results section (page 13), with supporting analyses provided in Supplementary Figure 27. We believe this addition substantially

strengthens the manuscript by linking the structural and simulation-derived interface map to a direct experimental test of residue-level determinants of vault stability.

5. The current discussion is limited. The authors should provide an in depth analysis on how their findings advance our understanding of vault regulation and why they observed committed states and half shells by cryoEM, but no other intermediate states.

Reply: We thank the reviewer for the constructive comment. In response, we have expanded the discussion to clarify how our findings advance the understanding of vault regulation. Our integrated cryo-EM and MD analysis, now including Principal Component Analysis (PCA) (Supplementary Figure 6 and 20), reveals that the "committed" state is a heterogeneous ensemble encompassing multiple asymmetric arrangements rather than a single discrete conformation. This inherent flexibility explains the observed lower resolution and implies that the state represents a broad conformational ensemble, not a structural endpoint.

We emphasize that in biotechnological applications, where the vault is used as a standalone shell, this intrinsic flexibility is a key feature. The ability to adopt multiple conformations enables the shell to encapsulate diverse cargoes, possibly respond to environmental cues, and function as a dynamic delivery platform. Our findings thus provide a molecular basis for the vault's applications in biotech, including targeted delivery, transcriptomic devices, and encapsulation systems.

Future studies will be needed to probe how external stimuli and interactions with partners (e.g., TEP1, vPARP) modulate the vault dynamics in cells (see comment 1 and 9, Reviewer 1). Finally, we also note that 3D classification may favor the most populated states, potentially missing rare or transient intermediates. These points are now clearly discussed in the revised manuscript.

Reviewer #4 (Remarks to the Author):

This manuscript, "Structural flexibility of the human vault protein revealed by high resolution cryo-EM and molecular dynamics simulations", is directed at an important problem in cell biology. The vault particle is an extremely stable, nano-scale particle, composed of a protein shell entirely composed of 78 copies of a single ~100 Kd protein (the major vault protein, MVP). However, fully assembled vaults have been shown, biochemically, to be very dynamic as they can internalize very large proteins (like PARP4 and TEP1) and even large particles (like 10 nm nano-discs and adeno associated virus).

The authors use cryo-EM and MD simulations in an attempt to reveal the structural factors that impart flexibility on the human vault. They describe two cryo-EM conformational states of the vault and MD simulations of these structures. They conclude that their data suggests a possible mechanism for the dynamic assembly and disassembly of the vault.

This review includes comments from an expert in molecular dynamics simulations who was included as an early career researcher (ECR) who co-reviewed this manuscript as part of the Nature Communications initiative to facilitate training in peer review and to provide appropriate recognition for Early Career Researchers who co-review manuscripts.

In general, this is an ambitious manuscript with a number of significant problems and some important minor issues as well.

First the vaults used in the analysis, which are expressed from human MVP in yeast, are only partially purified. Over 40% of the particles that they obtain are half vaults. As half-vaults are (almost) never seen in in situ images of vaults in cells and rarely seen in highly purified vaults from eukaryotic tissues, it is likely that these “half” vaults are products of vault degradation or denaturation.

Reply: We thank the reviewer for pointing this out. The presence of a sizeable portion of half vaults in the preparation was initially deduced from SAXS analysis; However, both SEC-MALS and cryo-EM consistently indicated that half vaults represent only a minor fraction in the sample (< 10 %).

Inspired by this comment, as well as the comment 1 of Reviewer 3, we performed additional SAXS measurements and a model-independent analysis to determine oligomerization states. Specifically, we collected SAXS data across a concentration series and estimated molecular weights primarily from the Porod volume, which is better suited for large particles and avoids dependence on two-component model fitting. Across all concentrations, the SAXS-derived molecular weights are consistent with predominantly intact vaults and agree with SEC-MALS, indicating that half-vaults are not present in solution to a significant degree under our conditions. We therefore conclude that our earlier two-component SAXS fit overestimated the half-vault fraction due to under constrained modeling (see also reply to Reviewer #3, point 1, and the new Supplementary Figure 2 and Supplementary Table 1).

We note that half-vaults have been found (Guerra et al., 2022), and evidence of exchange of half vaults in cell has also been reported (Yang et al., 2010). In our hands, half-vaults become more abundant when lowering pH or mutating key residues (see new Supplementary Figure 27). Therefore, we interpret half-vaults as condition-dependent, minor population. We have moved the half-vault data to the supplementary information.

How can we be assured that the second cryo-EM structure, the “committed” vaults, might just represent a class of damaged particles as well?

Reply: The reviewer raises a very interesting point, namely how to distinguish between conformational heterogeneity and particle damage in cryoEM datasets. While no single criterion is definitive, our state-of-the-art good practices as well as several independent and orthogonal observations argue that our committed map represents a genuine conformational sub-ensemble rather than a collection of globally denatured particles.

(i) Data-quality controls and particle curation. To minimize air-water interface damage, we prepared grids using affinity grids, which reduce particle exposure and were not used in Guerra et al. (2022). We curated micrographs to exclude poor CTF fits and regions with thick/heterogeneous ice, and we removed low-quality particles through iterative 2D classification, ab initio reconstruction, and low-resolution 3D classification with manual inspection of class averages. Under these filters, the committed class is not enriched for obvious "broken-shell" projections or poor-alignment classes. We cannot exclude that a small fraction of damaged particles remains, but they would contribute only a minority of projections.

(ii) The distortion is localized and consistent with flexibility, not global collapse. The committed reconstruction retains a well-defined shell architecture over most of the particle, with the principal **differences localized to the waist region**. We added map-diagnostic analyses (new Supplementary Fig. 14; Supplementary Discussion) showing that the committed map's reduced quality is associated with local anisotropy/heterogeneity at the waist rather than a global failure of sampling or reconstruction geometry (e.g., preferred orientation or missing views). Consistent with this, we now explicitly limit atomic-level interpretation in the lowest local-resolution region and restrict our claims there to domain-level rearrangements (see also reply to Reviewer 3, comment 2).

(iii) Continuous heterogeneity in the raw particle set supports an ensemble interpretation. We performed additional heterogeneity analyses (3DVA and PCA on the full particle stack; new Supplementary Fig. 6), which reveal a broad conformational landscape with dominant deviations from ideal symmetry that align with the features separating primed and committed classes. This argues that the committed class is best understood as a stable "projection" of a continuous manifold captured by classification, rather than a discrete set of damaged outliers.

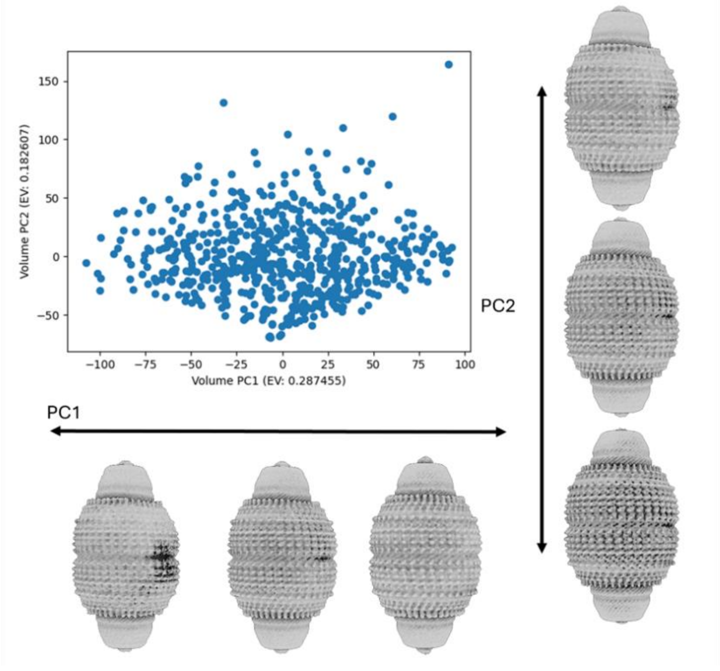

Supplementary Figure 6 - Principal Component Analysis (PCA) of the whole vault particle cryo-EM data set. *Principal Component Analysis (PCA) obtained with CryoDRGN after training an 8-dimensional latent model on the main dataset of 35,429 particles (prior to 3D classification). Representative 3D density maps are shown along the principal components (PC) of the latent space. The landscape represents the ensemble of conformations reconstructed from single-particle images. PCA on this space identified two dominant modes of conformational variability: PC1 and PC2, with explained variance (EV) ratio of 0.29 and 0.18, respectively, which indicates how much of the total conformational heterogeneity is described by each PC (or mode).*

(iv) Simulations show waist flexibility. All-atom MD simulations initiated from both conformations preserve the overall vault architecture (e.g., stable global dimensions and internal volume; Supplementary Fig. 17) and exhibit substantial collective motions and elevated flexibility in regions that differ between the reconstructions (PCA of trajectories; RMSF; updated Figure 5; new Supplementary Figures 19–20; Movie 3). We interpret these results as supporting the plausibility of the committed conformation as an intrinsically accessible asymmetric ensemble.

v) Independent observations in the literature are consistent with waist heterogeneity. A distorted/asymmetric vault-like conformation was also identified by 3D classification in an independent dataset (Guerra et al., 2022). Moreover, localized heterogeneity at the waist has been noted in recent structural work (e.g., "*Lower local resolution … suggests unresolved structural heterogeneity at the waist,"* Lövestam and Scheres, *2025*). Finally, in situ cryo-ET studies report vaults with midsection indentations/waist opening in cells (Woodward et al., 2015, see Figure R1 [REDACTED]), and subtomogram averaging further underscores shell flexibility in the cellular context (Geißler et al., 2025). These observations support the broader conclusion that vaults can populate asymmetric, locally distorted conformations.

Figure R1: Asymmetric vault distortions in simulations and in situ cryoET. Representative snapshots from simulations of primed (green/blue) and committed (yellow/orange) vault conformations are shown alongside corresponding in situ cryo-electron tomography views of cytosolic vaults from Woodward et al. (2015), highlighting comparable midsection/waist indentations (arrowheads).

Taken together, we conclude that the committed reconstruction reflects a genuine conformational sub-ensemble with localized waist heterogeneity.

The MD simulations are quite disappointing, revealing minor structural variations that do not explain the enormous openings of the particle that must be occurring to explain the biochemical dynamics previously reported.

Reply: We respectfully disagree with the reviewer's comment. As mentioned by the reviewer (see comment 1, Reviewer 4), the vault is indeed an exceptionally stable assembly under standard conditions ('half-vaults are (almost) never seen in in situ images'); Accordingly, large disruptions reported previously require strong perturbations (e.g., low pH, mechanical probing,

or targeted destabilization). Therefore, they not expected to occur in the observed timescales accessible to conventional MD. Capturing such rare, large-amplitude transitions in silico require enhanced sampling methods and different approaches.

Instead, the MD simulations serve a mechanistic purpose: they quantify the vault's intrinsic mechanical compliance and identify inter-subunit 'hot spots' at the waist and lateral interfaces. We have significantly improved the analysis of the MD trajectories to highlight key dynamic features, including long-lived interactions, transient interfacial disruptions, residue-specific flexibility, and correlated motions, which were not clearly presented in the initial version (see also comments below for detailed explanations and updated Figure 5 and new Supplementary Figures 19-24). These additions help to rationalize how interface interactions can enable large-scale opening once an external perturbation lowers the barrier.

Importantly, in the revised manuscript, we use these MD-derived contact networks to guide mutagenesis. A single point mutation at the waist (I36D), targeting the most persistent hydrophobic inter-half contact in the MD simulations, shifts the equilibrium strongly towards half-vault formation (see Reviewer 3, comment 4 and new Supplementary Figure 27). This experimental result supports the central utility of the simulations: they pinpoint specific interactions that control vault stability and reveal concrete molecular "entry points" through which perturbations can trigger opening.

One minor, but significant point is the underlying incorrect assumption by the authors that the vault particles self-assemble. It was shown over ten years ago that vaults do not self-assemble, rather vaults are assembled on polyribosomes. It is likely that free MVP chains do not exist in cells and the notion that vaults in cells come apart into halves and can exchange halves has not been rigorously established.

Reply: We thank the reviewer for this comment. We included in the revised manuscript the reference to the study showing the assembly of the vault on the polyribosome (Mrazek et al., 2014) and removed any mention of self-assembly. Additionally, we remarked the speculative nature of the model describing half-vault exchange and only referenced the publication mentioned by the reviewer (Yang et al., 2010) in the Introduction.

With regard to the cryo-EM analysis, in lines 136–138, the authors state that they performed an unsupervised 3D classification of the particles and identified two major sub-populations, distinguished by the presence or absence of D39 point-group symmetry. However, the manuscript does not specify the relative proportions of particles assigned to each class. The authors should provide the percentage or particle counts for these two sub-populations. This information is important for evaluating the structural heterogeneity and the robustness of the classification.

Reply: In the main text we now included the ratio between the particles assigned to each conformation requested by the reviewer (at line 130), which was previously shown only in figures:

> *p.3: "We identified two major subpopulations, distinguished by presence or absence of the D39 point-group symmetry that is a characteristic feature of the vault. These subpopulations comprise 32% and 68% of the particles, respectively."*

Moreover, in the revised manuscript we added a PCA of the whole particle stack (new Supplementary Figure 6), showing that the major differences found in the particle populations are both recapitulated in the committed conformation, which further supports the clustering of the 3D classification.

The observation of both symmetric and asymmetric conformations suggests potential biological relevance beyond mere structural variability. While the symmetric state may reflect the most stable conformation of the vault particle, the manuscript would benefit from further discussion on the possible functional or mechanistic implications of the asymmetric state. Any relevant hypotheses or comparisons with prior studies would strengthen the interpretation.

Reply: We agree that the observation of both symmetric and asymmetric conformations goes beyond structural variability and likely holds functional significance. In the revised manuscript, we have expanded our discussion (starting at page 13) to emphasize a key conclusion: the vault particle exhibits remarkable flexibility, enabling it to adopt multiple conformations while maintaining overall structural integrity. This dynamic behavior may facilitate essential physiological functions, such as the controlled exchange of water and ions with the surrounding environment. This exchange is similar for both conformations (see updated Figure 5 in the main text), which is consistent with the particle's proposed role as a molecular shuttle or protective compartment. This view is supported by prior studies: Woodward et al. (2015) reported distinct vault conformations in situ; Lodwick et al. (2025) observed lower local resolution at the waist, suggesting inherent flexibility in this region; Geißler et al. (2025) only obtained a low-resolution map from cryo-ET; and Llauró et al. (2014, 2016) demonstrated the particle's capacity for self-healing, further underscoring its dynamic nature.

Regarding the opening mechanism proposed by Guerra et al. (2022), which suggests a committed conformation as an on-pathway intermediate, we acknowledge its appeal. However, our current data do not provide sufficient evidence to confirm this specific pathway. Instead, we now include a dedicated section (Section *"Frontal interactions between R1 domains regulate the equilibrium between full and half vaults"*) in the revised manuscript that integrates insights from MD simulations and mutagenesis to identify the key interactions at the waist responsible for stabilizing the particle. By combining long-lived contact analysis with point mutations, we highlight residues critical for maintaining interface integrity (see also the new Supplementary Figure 27). These additions strengthen the interpretation of the

asymmetric states as biologically relevant, and position the vault as a dynamic, adaptable nanocage.

A more detailed analysis of the MD simulations follows:

First, the authors should be commended for undertaking all-atom molecular dynamics simulations on such a large and complex system - this is indeed a significant technical effort and apparently, a first vault simulation in the field.

Reply: We thank the reviewer for the kind comment and the appreciation of our work. In particular for acknowledging the technical challenges of performing all-atom molecular dynamics (MD) simulations on such a large and complex system.

However, the depth and scope of the results do not adequately support the conclusions drawn, especially considering the scale and ambition of the simulations. Here are a few major problems associated with the computational part of the paper.

1. Simulation time reporting and adequacy: The manuscript states: “We performed two replicates of AA-MD simulations for each initial conformation, each running for 250 ns, yielding a cumulative simulation time of 1 μs.” This method of summing replicate durations to report total simulation time is misleading. Each individual trajectory spans only 250 ns, which is insufficient for capturing the full range of structural dynamics in systems of this size and complexity. At a minimum, simulations of at least 500 ns per replica would be expected to ensure adequate sampling of conformational space at all-atom scales since they do CG simulations as well. (https://www.nature.com/articles/s41586-024-07150-4)

Reply: In response to the concern about simulation time, we have revised the manuscript to clearly report the length of each individual replicate.

Following, the reviewer’s advice, we have also extended the **all-atom simulations** to **500 ns per replicate**, doubling the original simulation length. Importantly, this extension did not alter the key findings reported in the manuscript. The structural flexibility, interfacial interactions, and conformational behavior observed remain consistent. These updates strengthen the reliability of our conclusions and ensure that the results are based on a more robust sampling of conformational space. We believe the revised data now better support the interpretations presented (see updated Figure 5 and Methods).

2. Interpretation and strength of conclusions: Several statements in the manuscript appear speculative or overly interpretive relative to the data shown. For example: “These fluctuations suggested the presence of a general breathing motion.” “Both the cap and helix regions

displayed smaller diameter fluctuations than the waist, supporting the hypothesis that the vault's greater flexibility at the waist is closely linked to its opening mechanism."

While these are interesting hypotheses, they would be more compelling if supported by more experimental data or prior simulation studies demonstrating vault opening behavior. In the absence of such evidence, the claims should be clearly framed as speculative or proposed interpretations. Additionally, if the proposed breathing motion is functionally meaningful, one might expect to see correlated solvent or ion exchange dynamics features that are not currently analyzed. Drawing parallels with well-characterized systems like nucleosomes (which also exhibit breathing) may offer useful context but should be supported by analogous data. Overall, these interpretations should be tempered and more clearly distinguished between observations and hypotheses.

Reply: We have extended the analysis of the MD simulations by performing Principal Component Analysis (PCA), which reveals a clear breathing motion in both symmetric and asymmetric conformations of the vault particle. These results are now presented in the new Supplementary Figure 20 and Supplementary Movie 3:

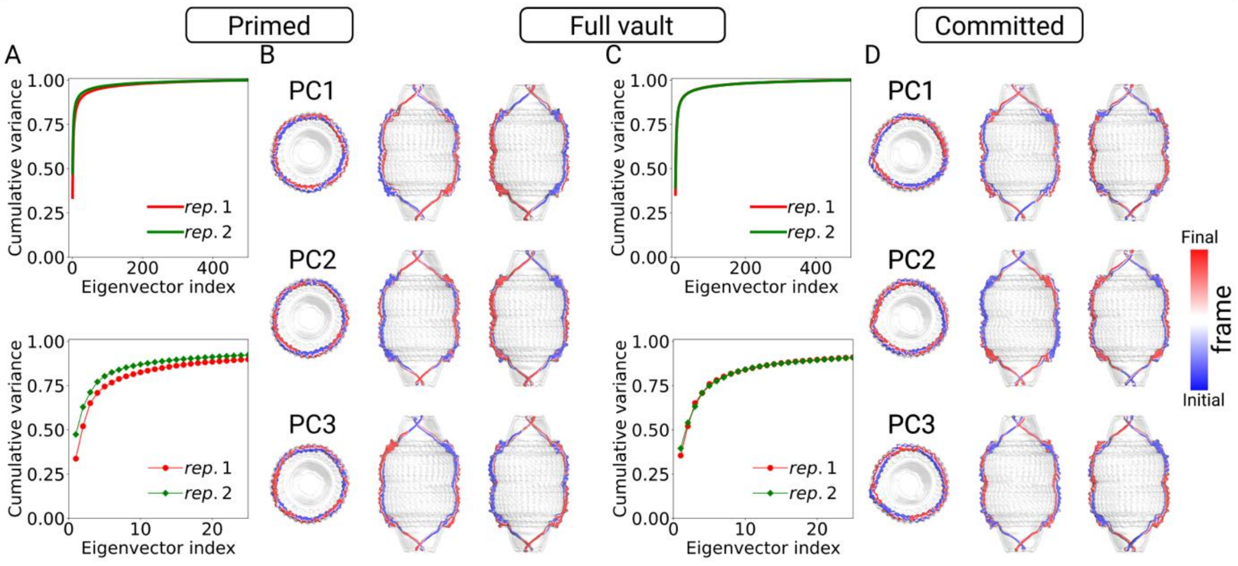

Supplementary Figure 20 – Principal-component analysis (PCA) of all-atom MD trajectories for the full vault particle. A, C) Cumulative variance. PCA was performed on the centre-of-mass (CoM) coordinates of every fifth residue along each MVP chain over the entire simulation. Top: Fraction of total variance captured as a function of the number of PCs (ordered by decreasing eigenvalue). Bottom: Same plot restricted to the first 25 PCs for the primed (A) and committed (C) conformations. B, D) Structural projections onto the three leading PCs. For each conformation, the first (PC1), second (PC2) and third (PC3) eigenvectors are visualised as colour-mapped deformations of single MVP chains; the average structure is shown as a transparent surface. Three orthogonal views are displayed for each PC (left, centre, right). Left: view from the bottom of a half-vault. Centre: view of two chains located on opposite sides of the particle. Right: view of two chains positioned 90° around the vault relative to the centre view. The colour scale runs from blue (minimum projection) to red (maximum projection), highlighting the regions that contribute most to each principal motion.

We also add it to the main text.

> *p. 10: "Additionally, we observed breathing-like motion in the trajectory. These motions were captured with PCA and projected onto the top 3 principal components (PCs), which account for about 70% of the variance (**Supplementary Figure 20**). The collective motions show asymmetric deformation of the vault particle and torsional motions with global fluctuations of the entire particle (see **Supplementary Movie 3**)."*

Furthermore, we added calculations on the internal volume of the particle, water exchange, and ion exchange (see updated Figure 5). However, we did not observe correlation between the breathing motion and ion exchange dynamics. The rates of ion and water exchange between the vault inside and outside, and the vault luminal volume remained steady throughout the simulations. This suggests that the observed fluctuations are not directly coupled to solvent or ion permeation under the current conditions.

We have revised the manuscript to clearly distinguish between observed structural dynamics and interpretive claims. All references to the opening mechanism proposed by Guerra et al. (2022) are now explicitly framed as speculative or hypothetical, in line with the data. We believe these changes strengthen the accuracy of our interpretation.

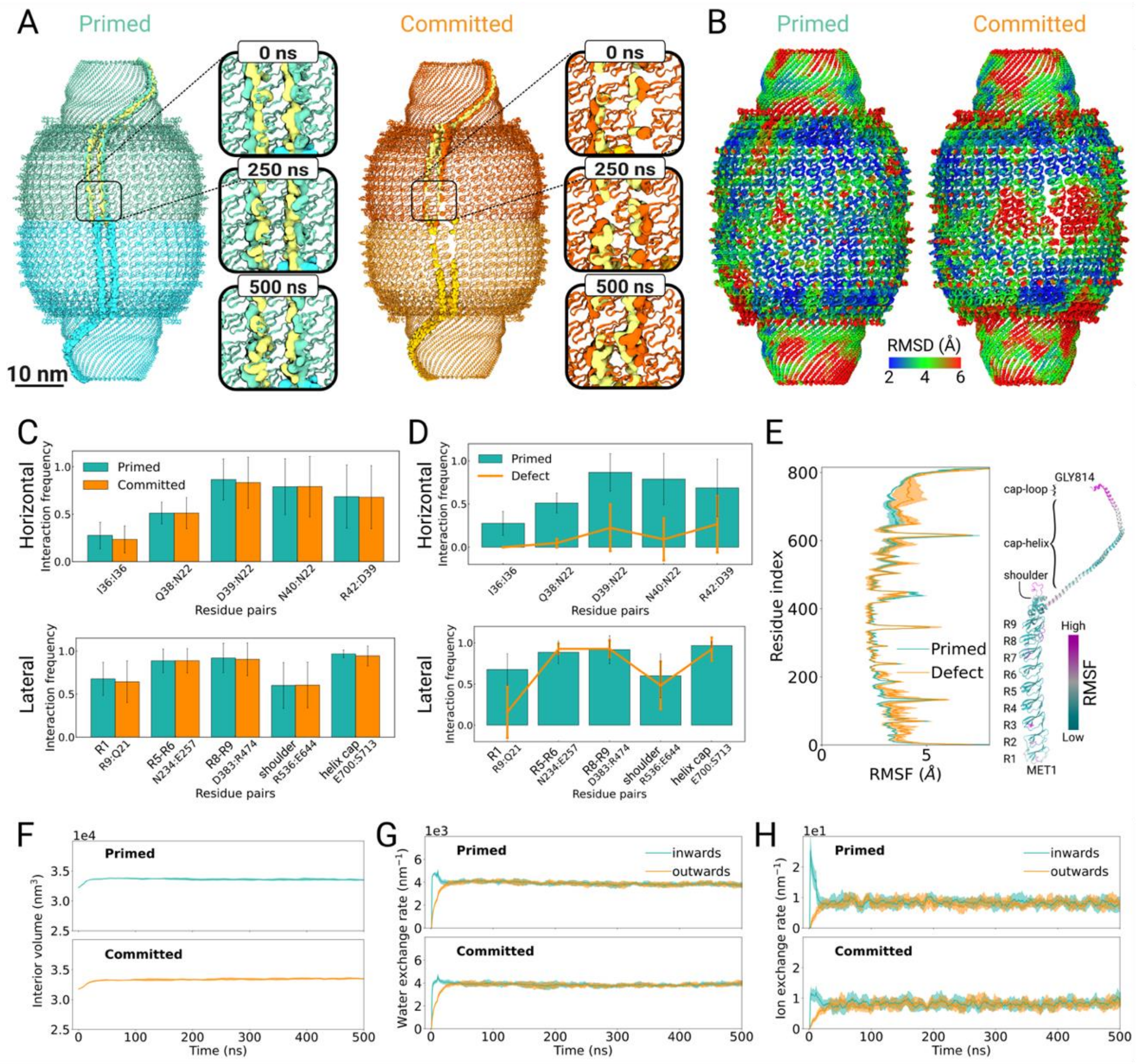

Figure 5 –All-atom MD simulations of the vault in primed and committed conformations. A) Initial atomic models (cartoon) of the vault in the primed (left) and committed (right) conformations. Surface renderings highlight inter-MVP contacts; inset panels show representative snapshots from the MD trajectories. B) RMSD over the last 450 ns of simulation, mapped onto the initial atomic structures of the vault in primed (left) and committed (right) conformations. Structures are aligned to the Cα atoms of residues with RMSF <3.5 Å. C) Persistent inter-MVP contacts identified in the simulations. Top: Waist region – most frequent contacts per interaction type (hydrophobic I36:I36, polar Q38:N22, D39:N22, N40:N22, and salt-bridge R42:D39). Bottom: Lateral interfaces – dominant contacts in regions R1 (R9:Q21), R5-R6 (N234:E257), R8-R9 (D383:R474), shoulder (R536:E644), and helix-cap (E700:S713). Bars correspond to van der Waals (vdW) contacts frequency in the primed (blue) and the committed (orange) conformation. D) Effect of vault rupture on vdW interactions. Top panel: Waist contacts – blue bars (vdW persistence in primed vault); orange line (vdW frequency between chains bordering the opening in committed conformation). Bottom panel: Lateral contacts – same colour scheme. The most pronounced loss of vdW interactions occurs at the N-terminal R1 domain in the waist region. E) Relative mean-square fluctuation (RMSF) per residue for the full trajectory. Left: RMSF vs. residue index. Average over all chains in two primed replicates (blue) and chains adjacent to the rupture/defect in the committed conformation (orange). Right: RMSF values colour-mapped onto a single MVP monomer. F) Interior volume of the vault particle as a function of time over the MD trajectory in primed (top) and committed conformation (bottom). G) Water and H) ions exchange across the vault shell. The exchange rates inwards (blue) and outwards (orange) were quantified by counting the number of events in which water/ions crossed from the exterior to the defined interior volume of the vault in the primed (top) and committed (bottom) conformations.

3. Underwhelming analysis of all-atom simulations: The manuscript presents only RMSD as an output from the all-atom simulations, which is a surprisingly limited use of what should be a rich dataset. All-atom MD provides access to a wide range of structural, dynamic, and thermodynamic observables - far beyond RMSD. Analyses such as ion/water exchange, hydrogen bonding patterns, local flexibility profiles, and correlated motions could provide valuable insights, especially for a system as complex as the vault.

Reply: We thank the reviewer for the valuable suggestions. In response, we have significantly expanded the analysis of the all-atom MD simulations to fully leverage the richness of the data. We now include:

- Identification of key residues involved in hydrogen bonding and van der Waals interactions stabilizing lateral and frontal MVP interfaces (main text Figure 5, panels C and D; Supplementary Figures 22-24).
- Per-residue backbone mean square fluctuations to compare local flexibility in both conformations (main text Figure 5, panel E; Supplementary Figure 19).
- Quantification of internal vault volume and measurement of water and ion exchange rates (main text Figure 5, panels F–H).
- Quantification of the average ion density at the surface of the vault particle (Supplementary Figure 21).
- Principal component analysis to reveal correlated motions (Supplementary Figure 20, Supplementary Movie 3).

These new analyses provide a comprehensive view of the system's dynamics and directly address the reviewer's concerns.

Additionally, equilibration of such systems is typically evaluated through stabilization of water/ion exchange or other internal metrics, not merely through RMSD plateaus. The authors should look into details of those simulations and show results indicative of breathing. The authors are encouraged to incorporate more robust metrics and comparative analyses, as demonstrated in the following studies:
https://pubs.acs.org/doi/full/10.1021/acs.jctc.3c00846,
https://www.nature.com/articles/s41586-024-07150-4,
https://www.science.org/doi/10.1126/science.adq7597
These works highlight best practices in leveraging all-atom simulations to gain mechanistic insight, and the current manuscript would benefit from adopting similar approaches.

Reply: We have analyzed the internal volume and water/ion exchange dynamics, which show stabilization after approximately ~50 ns (now shown in updated Figure 5 panels F-H and described in the main text). This indicates equilibration of the system. We also note that our simulation protocol includes a considerable pre-production phase for equilibration of the

system. Unlike closed viral capsids, the vault's particle is open at the caps with transient pores at the barrel, enabling comparably rapid solvent and ion exchange. This intrinsic permeability explains the faster equilibration observed (see updated Figure 5).

4. The manuscript states: “In the MD simulation trajectories, the vault underwent significant breathing motions in solution, yet the particle did not transition between the two conformations, nor did the two halves initiate the opening in the absence of external stimuli.” After careful review of the trajectory data, including extended viewing of the provided simulation videos at multiple playback speeds, supplementary, this claim of “significant breathing motions” does not appear to be convincingly supported. While some local fluctuations are expected in such large systems, the overall structural changes depicted in the simulation videos appear subtle and well within the range of thermal noise. Without quantitative metrics - such as internal volume fluctuations, inter-subunit distance variation, or principal component analysis - it is difficult to substantiate the use of the term “significant.” The authors are encouraged to provide more rigorous, quantifiable evidence to support this observation to better reflect the modest level of motion observed in the simulations.

Reply: Principal component analysis shown in Supplementary Figure 20 and Supplementary Movie 3, clearly reveals collective, large-scale motions consistent with a breathing-like behavior. Per-residue RMSF analysis further confirms considerable flexibility (Figure 5E and Supplementary Figure 19), particularly in the waist and cap regions, which correlates with the observed dynamics in the PCA. Although internal volume and ion/water exchange remain stable throughout the simulations, indicating no net permeability change, the presence of these concerted motions demonstrates that the vault undergoes dynamic fluctuations without transitioning to an open state. These findings, now supported by multiple complementary analyses, confirm that the observed motions describe the conformational dynamics.

We clarified the findings in the Results section:

> *p.10: “… We checked if the breathing-like movements were related to the particle's ability to exchange solvent with the environment. We measured the internal volume of the vault particle during the simulation and the water and ion exchange rates over time. Water exchange reached equilibrium at ~400 molecules/ns (**Figure 5G**), a value comparable to that reported for a bacteriophage particle[66]. Sodium and chloride ions achieved equilibrium exchange rates of ~10 ns^{-1} after 50 ns (**Figure 5H**), further confirming vault permeability to water and ions. Sodium ions localized to specific hotspots, e.g., D39:R42 salt bridges, R3–R4/R6–R7 interfaces, shoulder, and helix-cap regions (**Supplementary Figure 21**). Exchange rates stabilized after ~50 ns, indicating simulation equilibration.”*

5. The manuscript includes statements such as: “These pores could serve as a mechanism for pressure release and passive diffusion of ions and small molecules.”, “The vault's

attractiveness as self-assembling human nanoparticles... emphasizes the importance of understanding their dynamics."

While these are intriguing hypotheses, the authors should revisit the relevant literature and more accurately contextualize these claims. For instance, the term "self-assembling human nanoparticles" is wrong. Furthermore, their use in applications such as AAV (virus) trapping is ongoing research and should be framed accordingly (https://www.biorxiv.org/content/10.1101/2023.11.29.569229v4). These claims require clearer language and proper experimental support. The authors should revisit the literature thoroughly—this is not optional.

Reply: We revised the manuscript in accordance with the reviewer's comment, revisited the literature (Kickhoefer et al., 2005; Champion et al., 2009; Kickhoefer et al., 2009; Han et al., 2011; Kar et al., 2012; Doll et al., 2013; Muñoz-Juan et al., 2019; Tomaino et al., 2024; Collins et al., 2024, Chao et al. 2026) and revised the introduction (from line 59) to accurately contextualize prior and emerging applications of engineered vaults. We removed the term self-assembling human nanoparticles.

In addition, in the revised Discussion of the manuscript (line 536) we described the potential functional implications of the permeability of vault particle, especially in the light of a recent publication describing the presence of a binding site for NAD+, a substrate of vPARP, in the inner lumen of the vault.

> *p 14: "While the physiological relevance of such transient openings remains to be established, they suggest a plausible route for limited solvent exchange of small molecules between the lumen and the surrounding solution. Interestingly, a recent structure of the human vault in complex with vPARP has shown the presence of NAD+, a substrate of vPARP, bound within the vault lumen, consistent with the idea that vault-associated components may require solvent access for activity[70]."*

6. The methods section refers to multiple pH values across different experimental steps, yet it does not specify the pH at which the protein was protonated for the all-atom MD simulations. Was it pH 7, 7.5, or 8? Given the pH sensitivity of protein protonation states and their influence on dynamics and electrostatics, this omission is critical and should be addressed explicitly.

Reply: We agree with the reviewer. We have now explicitly stated in the Methods (line 741) that the all-atom MD simulations were performed with the proteins protonated at pH 7. This choice is in close correspondence with the experimental conditions used for cryo-EM data collection, which were carried out at pH 7.5.

Reviewer #5 (Remarks to the Author):

This manuscript focuses on the vault ribonucleoprotein complexes, reporting cryo-EM structures of both primed and committed conformations (of complete vault and half-vault particles), as well as all-atom and coarse-grain simulations of these systems. Although previous work has reported cryo-EM reconstructions of these conformational states of vault, this appears to be the first time that all-atom simulations of the intact vault complex have been reported. Given that the explicitly solvated system contains 18.4 million atoms, these simulations represent a serious investment of supercomputer time and an extremely valuable dataset to investigate the detailed dynamics of the complex vault assembly. However, the only analyses performed on the all-atom trajectory are root-mean-squared-deviation (RMSD), root-mean-squared fluctuation (RMSF), and ion permeability – this is an serious lost opportunity.

Since the cryo-EM structure analysis focus on per-residue contacts, it would be very interesting to conduct a similar analysis on the all-atom MD trajectory, to see how many of the contacts captured by cryo-EM persist during MD and with what frequency. Protein dynamics are essential to function. Clearly the vault complex is a highly dynamic system, such that it is best described by ensembles of conformers that define its conformational states. MD provides insights into these ensembles not available by other structural biology techniques. It would be a shame not to extract this type of data from the simulation trajectory given the notable effort and computational expense of obtaining it. The simulations of intact vault are relatively short (2 x 250 ns each), but data from symmetric copies can be combined to obtain a large amount of cumulative sampling for these local dynamics (e.g. 39 copies of MVP x 2 x 250 ns = 19.5 microseconds).

Reply: We thank the reviewer for the insightful comments and fully agree that a deeper analysis of the MD trajectories would significantly enhance the value of this work. In response, we have performed a per-residue contact analysis to assess the stability of key interactions observed in the cryo-EM structures. (Figure 5 and Supplementary Figures 22-24). We identified residues critical for lateral and frontal MVP interactions through hydrogen bonding and van der Waals contacts (Figure 5, panels C–D; Supplementary Figure 22-24). This analysis confirmed the persistence of key features at the waist, such as the polar network (N22, Q38, N40) and the D39–R42 salt bridge, throughout the simulations.

Notably, I36 emerged as the most consistently interacting residue within the hydrophobic patch. Combined with mutagenesis data, we demonstrated that disrupting this interaction impairs full vault assembly. These findings, discussed in the main text (starting at line 468, see also the reply to Reviewer 3, point 4), highlight how MD reveals dynamic stability of functionally critical contacts. We believe this analysis substantially strengthens the link

between structural observations and functional relevance, making full use of the rich simulation dataset.

Beyond residue contact frequencies, MD simulations also allow characterization of the collective motions and emergent dynamics of the assembly that are also relevant for function. The authors refer several times to “breathing motions” but do not characterize the collective motions that truly constitute “breathing’. They could use PCA or related dimensionality-reduction approaches to characterize distinct modes of vault motion, or other correlated emergent dynamics such as acoustic modes. For example, see Perilla & Schulten (https://www.nature.com/articles/ncomms15959) for examples of such analyses applied to large, intact protein shells. Examining collective motions from all-atom MD trajectories can provide mechanistic insights, and this aspect is not exploited in the manuscript in its current form.

Reply: As recommended, we have performed PCA on the all-atom MD trajectories, now shown in Supplementary Figure 20 and Supplementary Movie 3. The analysis reveals clear collective motions consistent with a breathing-like behavior, which we describe in the results section (line 432) and more in general in the Discussion.

Why not also do the ellipse-diameter analysis on the all-atom trajectory? Changes in not only elliptical diameter along the vault waist but also cross-sectional area of the ellipse could be correlated with changes in per-residue contacts along the waist to provide valuable information regarding the molecular determinants of this interface that represents the seam between the two vault halves, which apparently separate to encapsulate cargo.

Reply: We thank the reviewer for raising this insightful point. The ellipse-diameter analysis on the all-atom trajectory was already included in Supplementary Figure 17. We have now explicitly mentioned this analysis in the main text (line 364):

> *p. 10: “… the AA-MD simulations accurately captured the general shape of the vault particle with remarkable agreement with the experimental structures, as shown by particle diameter measurements at the waist, helix, and cap regions (**Supplementary Figure 17**).”*

The measured diameters align well with the cryo-EM structures. While the committed state shows slightly larger diameter fluctuations, both conformations remain stable throughout the simulation, with no signs of opening. We emphasize that these fluctuations reflect the vault’s intrinsic flexibility rather than an opening mechanism, a point now expanded in the revised manuscript.

The reviewer's suggestion to correlate diameter changes with contact loss at the waist is valid and highlights a key mechanistic question. However, because diameter is derived from a global elliptical fit and may obscure local molecular details, we focused instead on residue-level contact persistence and flexibility. Our analysis reveals a loss of interfacial contacts at the waist, particularly in the defect region of the committed conformation, correlated with increased RMSF compared to the primed conformation (Figure 5, panels D-E). These results point to contact loss and local deformation as key molecular determinants of vault asymmetry. Further studies, potentially using enhanced sampling, will be needed to explore the pathway to full opening (see also Reviewer 3's comment after Figure R1).

The authors conclude, based on larger RMSF values for the committed conformation relative to the primed conformation, that the committed conformation is less stable. However, there is something of a chicken versus egg problem here. The authors attribute lower resolution of the committed conformation map to the larger fluctuations in dynamics observed with MD. But Singharoy & Schulten (e.g. https://elifesciences.org/articles/16105) have previously shown that structural instability detected by RMSF can be a product of the low-resolution model used as initial coordinates for the MD. In this way, large overall RMSF of a structure can be indicative of the quality of the fit of the model to the EM map it was derived from, rather than validation of the dynamics of the ensemble of conformers averaged into the reconstruction. There may be no way to know based on the available data which is true here (if the structure is very flexible in MD because the map is low resolution, or if the map is low resolution because the real-life structure of the assembly is flexible), but the authors could be careful to at least caveat this rather than presenting it as a firm conclusion. Finally, flexibility does not necessarily mean instability.

Reply: We agree with the reviewer. To address the reviewer's concerns, we have performed a detailed analysis of per residue backbone RMSFs. Specifically, from all-atom MD simulations RMSF values were averaged over MVP chains of the i) full primed vault, ii) full committed vault, and iii) MVP chains lining the opening in the committed vault. We did not observe any noticeable difference in RMSF values between the full primed and committed vaults (Supplementary Figure 19), although we observed large fluctuations for both conformations even at those places where resolution is pretty good, e.g., at the helix and cap. Interestingly, chains around the MVP opening displayed a significantly increased fluctuations of N-terminal residues (particularly the first 50 residues), in line with reduced chain-to-chain interactions (Figure 5, panels D-E).

> *p. 10: "We further quantified flexibility by calculating average RMSF across all MVP chains for both conformations (**Figure 5E**, **Supplementary Figure 19**). RMSF confirmed the waist and cap regions as primary flexible sites. Mapping RMSF onto a single MVP chain (**Figure 5E**, right panel) revealed high flexibility in waist loops, R domains loops,*

*shoulder, and the cap loop. We note that the flexibility at the cap could be the result of not including the full cap domain in the simulations, the structure of which has recently been reported[11,12]. Interestingly, comparison of primed conformation RMSF with committed conformation RMSF at the symmetry-breaking interface (**Figure 5E**, left panel) shows increased flexibility at the rupture particularly at the waist and cap regions. This increased flexibility, particularly at the waist was consistent with the lower resolution of the cryo-EM model and the stretching-compression motions observed in the 3DVA."*

We have revised the manuscript to carefully rephrase our conclusions, emphasizing that observed flexibility does not equate to instability. The discussion now reflects the nuanced interpretation that dynamic behavior may reflect intrinsic conformational plasticity rather than structural instability, particularly in the context of low-resolution reconstructions.

p. 9 "Notably, a high flexibility region in the committed state is found near the symmetry-breaking interface, coinciding with the low-resolution region in the cryo-EM map. However, high-resolution regions (e.g., small parts of the primed conformation waist and both conformations around the shoulder and cap regions) also exhibited significant flexibility, confirming that vault dynamics are intrinsic and not merely an artifact of cryo-EM resolution. These observations collectively indicate that the vault particle maintains inherent structural flexibility across all conformations."

Overall, this is excellent work but appears rather incomplete because very little analysis was performed on the most unique dataset presented – the MD simulations. The authors should do a bit more with these MD trajectories to bring the work full circle, relating MD to the cryo-EM data in a more detailed and precise way and seeing what can then be inferred mechanistically from these details that are relevant to the opening/closing mechanism of vault. The authors should keep in mind that the EM maps/structures will be deposited and are available to researchers to analyze in detail on their own for follow-up studies. However, MD simulations particularly of this size are typically not released and most structural biology researchers lack the computing skills to conduct analysis; as such, readers rely entirely on the authors to distill the value from this remarkable large-scale trajectory. There is no double at incredible amount of valuable information in these MDs that must be brought to light to learn novel insights about vault not accessible to experimental methods.

Reply: We fully agree with the reviewer and thank them for emphasizing the importance of extracting maximal insight from this unique MD dataset. In response, we have significantly expanded the analysis to better integrate MD results with cryo-EM data, including contact persistence (Figure 5, Supplementary Figures 22-24), residue flexibility (Figure 5, Supplementary Figures 19), collective motions (Supplementary Figure 20 and Supplementary Video 3), volume measurements (Figure 5), ion/water exchange (Figure 5), and interface

dynamics guiding mutagenesis studies (Supplementary Figure 27), providing mechanistic insights into vault flexibility and stability.

We also confirm that the simulation trajectories will be deposited in Zenodo (prior to the manuscript publications) to support future studies. We believe these additions ensure the data's full value is realized by the broader community.

Reviewer #6 (Remarks to the Author):

I co-reviewed this manuscript with one of the reviewers who provided the listed reports. This is part of the Nature Communications initiative to facilitate training in peer review and to provide appropriate recognition for Early Career Researchers who co-review manuscripts.

Reply: We thank the reviewer reviewing our manuscript.
